# *Trigonella foenum-graecum* L. protects against renal function decline in a mouse model of type 2 diabetic nephropathy by modulating the PI3K-Akt-ERK signaling pathway

**DOI:** 10.3389/fphar.2025.1566723

**Published:** 2025-03-18

**Authors:** Yang Niu, Hongjuan Niu, Luxuan Chi, Peihang Li, Jiyang Du, Xiaoqian Wang, Xu He, Binan Lu, Zongran Pang

**Affiliations:** ^1^ Key Laboratory of Ethnic Medicine in Ministry of Education, School of Pharmacy in Minzu University of China, Beijing, China; ^2^ State Key Laboratory of Female Fertility Promotion, Center for Reproductive Medicine, Department of Obstetrics and Gynecology, Peking University Third Hospital, Beijing, China; ^3^ Pharmacy Department, People’s Hospital of Dali Bai Autonomous Prefecture, Dali, China

**Keywords:** diabetic nephropathy, *Trigonella foenum-graecum* L., network pharmacology, PI3K-Akt-ERK pathway, untargeted metabolomics

## Abstract

**Objectives:**

*Trigonella foenum-graecum* L. (HLB) exhibits promising pharmacological properties for the treatment of type 2 diabetic nephropathy (DN). This study aims to enhance the understanding of HLB’s pharmacodynamic effects and elucidate the mechanisms underlying its therapeutic potential in DN.

**Methods:**

The pharmacodynamic effects of HLB were initially evaluated in a murine DN model through the oral administration of an aqueous extract of HLB. The primary bioactive constituents were subsequently identified using ultra-high-performance liquid chromatography coupled with high-resolution mass spectrometry (UHPLC-HRMS). Network pharmacology analysis was integrated with these data to uncover potential molecular targets of HLB in DN. Key renal metabolites were profiled using untargeted metabolomics, followed by metabolic pathway enrichment analysis conducted with the MetaboAnalyst 6.0 platform, which facilitated the identification of relevant metabolic pathways through which HLB modulates DN. Finally, quantitative real-time polymerase chain reaction (QRT-PCR) and Western blot (WB) techniques were employed to validate the expression levels of key genes and proteins, thereby confirming the molecular mechanisms underlying the effects of HLB in DN.

**Results:**

Animal experiments indicated that HLB significantly improved blood glucose regulation and renal function while reducing oxidative stress and abnormalities in lipid metabolism in diabetic mice. A total of 34 compounds and 159 potential therapeutic targets were identified as key active components of HLB. The untargeted metabolomics analysis revealed 61 critical metabolites, among which the PI3K-Akt-ERK signaling pathway—known to be involved in diabetes—was highlighted as a crucial pathway. QRT-PCR and WB analyses demonstrated that HLB upregulated the expression of MAPK1, MAPK3, AKT1, and PI3K.

**Conclusion:**

These results suggest that HLB may alleviate DN by modulating oxidative stress and lipid metabolism. Its effects are likely mediated through the PI3K-Akt-ERK signaling pathway, along with the upregulation of MAPK1, MAPK3, AKT1, and PI3K expression. This study lays the groundwork for further investigations into the molecular mechanisms underlying HLB’s action in DN.

## 1 Introduction

Diabetes Mellitus (DM) is a chronic metabolic disorder primarily characterized by dysregulated glucose metabolism (Xu et al., 2024), frequently accompanied by a range of complications, with DN being a prevalent microvascular complication (Alsharidah, 2022). DN represents the leading cause of end-stage renal disease (ESRD), initially presenting with hyperfiltration and proteinuria, followed by a progressive decline in renal function. The pathogenesis of DN is complex, involving various mechanisms, including metabolic disturbances (Samsu, 2021), oxidative stress (Jin et al., 2023; Wang et al., 2024b), inflammatory responses (Huang et al., 2024), and cytokine activities (Araujo et al., 2020; Guo et al., 2023). The all-cause mortality rate in patients with DN is approximately 30 times higher than in diabetic patients without nephropathy (Sagoo and Gnudi, 2020), significantly elevating healthcare costs and mortality rates, thus posing a global healthcare challenge. Despite the severe impact of DN, therapeutic strategies remain underdeveloped, underscoring the necessity for in-depth studies on its pathogenesis to facilitate the development of more effective treatments and medications. The existing treatment regimens for DN mainly include approaches such as a reasonable diet, blood glucose control, and inhibition of the progression of kidney disease (Schork and Artunc, 2022). Metformin is the first-line drug for lowering blood glucose, but it can cause gastrointestinal adverse reactions (Sanchez-Rangel and Inzucchi, 2017), and moreover, it cannot be used in cases of severe renal insufficiency in the state of DN (Boddepalli et al., 2022). In addition, SGLT2 inhibitors are also commonly used drugs for lowering blood glucose, yet they may lead to urinary tract infections and hypoglycemia (Dholariya et al., 2023). In contrast, HLB has a long history of use (Nagulapalli Venkata et al., 2017), and thus has relatively high safety. Furthermore, HLB has a significant hypoglycemic effect (Yadav and Baquer, 2013; Robert et al., 2014; Rizvi and Mishra, 2013; Gupta et al., 2024), and also plays an active role in kidney protection (Mbarki et al., 2017; Hadi et al., 2020), providing new ideas for the prevention and treatment of DN.

HLB is a widely used natural herb. According to “Jiayou Bencao”, HLB is attributed to the kidney meridian. In modern research, HLB is commonly used in the management of DM(Arooj et al., 2024; Kumar et al., 2021). Some studies have demonstrated that fenugreek alkaloids exert protective effects on proximal tubular epithelial cells, mitigating epithelial-mesenchymal transition and renal fibrosis in diabetic kidneys (Gong et al., 2023). Additionally, extracts of HLB have been found to improve diabetes by stimulating the insulin signaling pathway (Vijayakumar et al., 2005) and suppressing the inflammatory response (Nagamma et al., 2021). Preclinical animal studies have indicated that HLB or its combination with other therapeutic agents can ameliorate DN (Jin et al., 2014; Zhou et al., 2013). However, the underlying mechanisms remain incompletely understood. Therefore, investigating the pharmacodynamics of HLB in treating DN and elucidating its mechanisms of action have become areas of significant research interest. Network pharmacology, a field rooted in systems biology, provides a comprehensive approach for analyzing biological systems and selecting specific signaling nodes for the design of multi-target drug molecules, playing a pivotal role in elucidating the pharmacological mechanisms of traditional Chinese medicine (TCM) herbal formulas (Zhao et al., 2023a). Metabolomics, a central research method in systems biology, advances the modernization of Chinese medicine and the development of personalized medicine, with a particularly prominent role in Type 2 Diabetes Mellitus (T2DM) research (Li et al., 2023). The integration of network pharmacology and metabolomics offers an enhanced strategy for understanding drug mechanisms and pharmacological actions. For example, Pan et al. (Pan et al., 2020) utilized this combined approach to elucidate the therapeutic mechanism of Huanglian Tang in T2DM.

In recent years, studies have found that traditional Chinese medicines can regulate the PI3K - AKT signaling pathway by inhibiting apoptosis, regulating glucose and lipid metabolism, suppressing inflammatory responses, and modulating cell proliferation and fibrosis, thereby inhibiting the progression of DN (Fu et al., 2022). To clearly investigate the efficacy and mechanism of action of HLB on DN, we plan to study how HLB regulates the signaling pathway and exerts its effect in improving DN from aspects such as glucose and lipid metabolism regulation. In this study, a multi-faceted approach integrating network pharmacology and metabolomics was employed to investigate the mechanisms underlying HLB’s therapeutic effects on DN. Initially, the therapeutic efficacy of HLB was verified in a DN animal model. Subsequently, the composition of HLB aqueous extracts was analyzed using UHPLC-HRMS, and network pharmacology was applied to identify the potential therapeutic targets of HLB in DN. Untargeted metabolomics was then utilized to profile key therapeutic metabolites, and pathway enrichment analysis combined with network pharmacology results was employed to identify the signaling pathways modulated by HLB in DN. Finally, QRT-PCR and WB techniques were applied to validate the expression of central targets and pathways. This study presents a comprehensive approach, offering valuable insights into the pharmacodynamic effects and mechanisms of action of HLB in the treatment of DN.

## 2 Materials and methods

### 2.1 Material reagents

#### 2.1.1 Instruments and reagents

Mass spectrometry-grade acetonitrile and methanol were procured from Fisher Chemical, while mass spectrometry-grade formic acid was obtained from Honeywell. The following instruments were employed in the study: ultra-high-performance liquid chromatography (UHPLC Vanquish, Thermo), mass spectrometer (Thermo Q-Exactive HFX, Thermo), cryogenic high-speed centrifuge (Centrifuge 5430 R, Eppendorf), electrophoresis apparatus (Mini Protein Tetra System 1658001), and cryogenic freeze dryer (D-37520).

Reagents used for RNA extraction and cDNA synthesis included the Animal Tissue Total RNA Extraction Kit, FastKing cDNA First Strand Synthesis Kit, and SuperReal Fluorescence Quantitative Premix Reagent Enhanced, all sourced from TIANGEN Biotech (Beijing) Co., Ltd. Lipid metabolism-related assay kits for total superoxide dismutase (T-SOD), malondialdehyde (MDA), glutathione (GSH), total triglycerides (TG), total cholesterol (TC), high-density lipoprotein-cholesterol (HDL-C), low-density lipoprotein-cholesterol (LDL-C), and oxidized low-density lipoprotein (OxLDL) were obtained from Nanjing Jiancheng Bioengineering Institute. Kits for nonesterified fatty acid (NEFA), urinary microalbumin (mALB), blood urea nitrogen (BUN), creatinine (CRE), uric acid (UA), and other renal function markers were also sourced from Nanjing Jiancheng Bioengineering Institute. The BCA protein quantification kit was purchased from Thermo Fisher Scientific, and the ELISA kits for insulin (INS) and glycated hemoglobin (HbA1c) were obtained from Jianglai Biology.

The following primary antibodies were used in this study: ERK1/2 (A4782), β-actin, and horseradish peroxidase-conjugated goat anti-rabbit HRP (AS014) from ABclonal Technology; PI3K (12,721) from Cell Signaling; and AKT (Ab80588) from Abcam.

#### 2.1.2 Experimental animals

SPF grade C57BL/6J male mice (7 weeks old, weighing 22.0 ± 0.8 g) were obtained from SPF (Beijing) Biotechnology Co., Ltd. (Laboratory Animal Production License No. SCXK (Beijing) 2019–0010). Mice were housed under controlled conditions, with a temperature of 22°C ± 2°C, humidity between 50% and 60%, and a 12-h light/dark cycle. Mice were acclimatized for 1 week prior to experimentation. All animal procedures were approved by the Biological and Medical Ethics Review Committee of Minzu University of China (Review No. ECMUC2021002AO), and experiments adhered to the Guidelines for the Management and Use of Laboratory Animals.

#### 2.1.3 Sample preparation

The experiment strictly adhered to the ConPhyMP guidelines (Heinrich et al., 2022). The seeds of HLB were collected from Wushi Town, Huzhu County, Haidong City, Qinghai Province. They were identified as the dried mature seeds of *Trigonella foenum-graecum* L. (a leguminous plant) by Professor Chunlin Long from Minzu University of China. The samples were stored in the laboratory at Minzu University of China.

To prepare the HLB extract, 500 g of HLB seeds were soaked in 10 times the amount of water for 30 min, boiled for 30 min, and the first filtrate was collected. The process was repeated with 8 times the amount of water, and the second filtrate was collected. Both filtrates were combined, concentrated under reduced pressure at 70°C to a specific volume, and freeze-dried into powder for storage and use in UHPLC-HRMS compositional analysis. The resulting extract was then utilized for animal experiments.

### 2.2 Animal experiments

#### 2.2.1 Replication and grouping of animal models

After 1 week of acclimation, mice were randomly assigned to normal and model groups based on their body weight. The normal group received a low-fat diet (LFD; model: D12450B, 10% kcal; SPF (Beijing) Biotechnology Co., Ltd.), while the model group was fed a high-fat diet (HFD; model: D12492, 60% kcal; SPF (Beijing) Biotechnology Co., Ltd.) for 12 weeks. Following this period, mice in the model group were intraperitoneally administered 50 mg/kg STZ for three consecutive days (Shao et al., 2022), whereas mice in the normal group were injected with an equivalent dose of citric acid-sodium citrate buffer. One week later, if the blood glucose level was ≥16.7 mmol/L, the urine output was >150% of the original urine volume, and the urinary protein was >30mg/(kg·24h), the mouse model of DN was successfully replicated (Noshahr et al., 2020; Kölling et al., 2017). Upon successful model establishment, the normal group continued on LFD, and the model group remained on HFD during the subsequent 4 weeks. A total of fifty mice were randomly divided into five groups, each containing 10 mice: Normal group (Control, Con), Model group (Model, Mod), Metformin positive control group (Metformin, Met, 0.25 g/kg) (Meng et al., 2020; Zhao et al., 2023b), HLB high-dose group (HLB-H, 0.52 g/kg), and HLB low-dose group (HLB-L, 0.26 g/kg). The dosage administered to mice was calculated in accordance with the requirements of the Pharmacopoeia of the People’s Republic of China (Volume I, 2020 Edition), using the equivalent - dose conversion coefficient between humans and mice. In this experiment, the clinical dose was set as the low - dose, and the high - dose was twice the amount of the low - dose.

#### 2.2.2 Sample collection and preservation

An oral glucose tolerance test (OGTT) was performed on all mice prior to the conclusion of the experiment. The mice were placed in metabolic cages for urine collection to assess various indices. Following the final gavage, the mice were fasted for 10 h. Blood was obtained from the orbital vein after anesthesia and weighing, and the serum was separated *via* centrifugation for subsequent biochemical analysis. The left kidney was fixed in 4% paraformaldehyde for pathological examination, while the right kidney was stored at −80°C for further assays.

#### 2.2.3 Testing of blood markers

Serum samples were analyzed according to the instructions of the serum biochemical assay kits, with a specific focus on glucose metabolism-related markers (INS and HbA1c). The insulin resistance index (HOMA-IR) and insulin sensitivity index (ISI) were calculated for each mouse using the following formulas:

$$HOMA-IR=\left(FPG\times INS\right)÷22.5$$

$$ISI=\ln\left[1÷\left(FRG\times INS\right)\right]$$

#### 2.2.4 Testing of urine markers

Urine samples from each group were centrifuged, and the supernatant was diluted with saline for the measurement of renal function-related indices (BUN, CRE, UA) using the respective assay kits.

#### 2.2.5 Kidney oxidative stress indicator assay

For biochemical analysis, mice kidney tissue was homogenized in saline at a 1:9 ratio to prepare a 10% kidney tissue homogenate. The homogenate was centrifuged at 12,000 rpm for 10 min at 4°C, and the supernatant was carefully collected. Following the kit instructions, the supernatant was appropriately diluted and analyzed for SOD, MDA, and GSH levels.

#### 2.2.6 Kidney histopathology

The kidney tissues fixed in 4% paraformaldehyde were subjected to ethanol gradient dehydration, xylene infiltration, and paraffin embedding, followed by sectioning into 5 µm thick slices.

These sections underwent pre-treatment, including baking, dewaxing, and ethanol gradient rinsing, before being stained with hematoxylin and eosin (HE), periodic acid-Schiff (PAS), and Masson’s trichrome staining. Pathological alterations were assessed by examining the degree of glomerulosclerosis and tubulointerstitial damage across different experimental groups.

### 2.3 UHPLC-HRMS composition characterization of HLB

#### 2.3.1 Chromatographic conditions and mass spectrometry parameters

HLB extracts were analyzed using a Thermo UHPLC Vanquish Ultra High-Performance Liquid Chromatograph coupled with a Thermo Q-Exactive HFX Mass Spectrometer. Chromatographic separation was achieved on an ACQUITY UPLC HSS T3 column (2.1 mm × 100 mm, 1.8 µm). The mobile phases consisted of (A) 0.1% formic acid aqueous solution and (B) 0.1% formic acid acetonitrile solution, with a flow rate of 0.3 mL/min. The column temperature was maintained at 35°C. The electrospray ionization (ESI) source operated in both positive and negative ionization modes, with parameters set as follows: ion spray voltage at +3,800/-3800 V, and a clustering potential of 40 V. Ion source gases (gas 1 and gas 2) were nitrogen, set at 45 psi, and the curtain gas pressure was set to 20 psi. The capillary temperature was set to 320°C, and the probe heater temperature was maintained at 370°C. The mass range was scanned from 90 to 1,300 m/z.

#### 2.3.2 Sample solution preparation

For sample preparation, 600 μL of the HLB extract was mixed with 400 μL of methanol and vortexed thoroughly. An aliquot of 100 μL of the mixture was then added to 700 μL of a 40% methanol aqueous solution for dilution, vortexed, and centrifuged at 16,000 rpm for 15 min at 4°C. The supernatant was collected for analysis.

### 2.4 Network construction and analysis

#### 2.4.1 Screening of pharmacodynamic components

HLB components identified *via* UHPLC-HRMS were further analyzed using several databases, including TCMSP (https://old.tcmsp-e.com/tcmsp.php) (Ru et al., 2014), ETCM (http://www.tcmip.cn/ETCM/index.php/Home/Index/index.html) (Xu et al., 2019), PharmMapper (http://www.lilab-ecust.cn/pharmmapper/) (Wang et al., 2017b; Liu et al., 2010), and SwissTargetPrediction (http://www.swisstargetprediction.ch/?) (Daina et al., 2019), to predict the associated biological targets. The above are the commonly used databases for target prediction. Among them, TCMSP and, ETCM focus on traditional Chinese medicine components and validated bioactive compounds; PharmMapper and SwissTargetPrediction predict targets through reverse docking. The strategy of using multiple databases in combination can reduce bias and improve the reliability of prediction. The screening criteria for pharmacodynamic components were as follows: in TCMSP, the oral bioavailability (OB) was required to be ≥30% and the drug - likeness (DL) ≥ 0.18 (Gu et al., 2024; Xiang et al., 2022); in PharmMapper, the z - score was set to be ≥0.9 (Fan et al., 2023); in SwissTargetPrediction, the probability was required to be >0 (Xie et al., 2022).

#### 2.4.2 Screening for disease-related targets

To identify relevant genes in DN, the terms “diabetic nephropathy” and “diabetic kidney disease” were searched in OMIM (http://www.OMIM.org/), DisGeNET (http://www.dissenet.org/), GeneCards (https://www.genecards.org/), and DrugBank (https://go.drugbank.com/) databases. The aim of using multiple commonly - used databases in combination is to enhance the reliability of the experiment (Wu et al., 2024; Knox et al., 2024). These targets were normalized using the Uniprot database, and duplicates were removed. The drug and disease targets were then intersected using the Venny 2.1.0 online platform (https://bioinfogp.cnb.csic.es/tools/venny/index.html) to identify potential targets for HLB in the treatment of DN.

#### 2.4.3 Network construction and core gene screening

Protein-protein interactions (PPI) among the common targets were analyzed using the STRING 11.0 (https://string-db.org/) online platform, with an interaction confidence score threshold of 0.9 (Wang et al., 2024a). Core genes were selected based on two topological parameters: degree and closeness. Cytoscape 3.9.1 software was used to construct a predicted chemical-target-disease (CTD) network.

#### 2.4.4 Annotation of GO KEGG biological processes

To investigate the mechanism by which HLB affects DN, Gene Ontology (GO) bioinformatics enrichment analysis was performed on the common targets using R software. Additionally, the KEGG database was utilized to identify the major biochemical and metabolic pathways in which these protein targets are involved, with a significance level set at p < 0.05. Visualization of the results was carried out using R software.

### 2.5 Untargeted metabolomic analysis

#### 2.5.1 Collection and processing of metabolic samples

Mouse kidney tissue (50 mg) was homogenized with 400 µL of extraction solution (methanol:water = 4:1, V:V) containing the internal standard (L-2-chlorophenylalanine at 0.02 mg/mL). The mixture was ground at −10°C for 6 min at 50 Hz, followed by ultrasonic extraction at 5°C and 40 kHz for 30 min. After resting at −20°C for 30 min, the samples were centrifuged at 13,000 rpm for 15 min at 4°C, and the supernatant was collected for analysis.

#### 2.5.2 Chromatographic conditions

Chromatographic separation was achieved using an ACQUITY UPLC HSS T3 (100 mm × 2.1 mm, 1.8 µm; Waters, Milford, United States) column, with mobile phase A consisting of 95% water and 5% acetonitrile (containing 0.1% formic acid) and mobile phase B comprising 47.5% acetonitrile, 47.5% isopropanol, and 5% water (containing 0.1% formic acid). The injection volume was 3 μL, and the column temperature was maintained at 40°C.

#### 2.5.3 Mass spectrometry conditions

Ionization was performed *via* ESI, with mass spectral data collected in both positive and negative ionization modes. Specific parameters are outlined in Supplementary Table S1.

#### 2.5.4 Differential metabolite screening and untargeted metabolic pathway enrichment

We selected the Con group (n = 8), Mod group (n = 8), HLB group (n = 8), and Quality Control (QC) group (n = 3) for analysis. For the pre - processing of raw data and screening of differential metabolites, we adopted the common methods on the Majorbio Cloud Platform (Duan et al., 2024; Chen et al., 2022). Raw data were processed using Progenesis QI (Waters Corporation, Milford, United States) software, with metabolites identified and annotated through the HMDB (http://www.hmdb.ca/) and Metlin (https://metlin.scripps.edu/) databases. Labeled metabolites were then uploaded to the Majorbio cloud platform (www.majorbio.com) for further data processing.

Principal component analysis (PCA) was performed using the R package ropls (version 1.6.2), and partial least squares discriminant analysis (PLS-DA) was employed to predict sample categories. Differential metabolites were identified based on VIP >1 and P < 0.05. KEGG pathway enrichment analysis was carried out to identify key metabolic pathways.

#### 2.5.5 Correlation analysis

To investigate the mechanism of HLB in DN, correlations between identified metabolites and potential targets were explored. Key metabolites, along with network pharmacology-derived targets and metabolomics pathways, were subjected to combined pathway analysis in MetaboAnalyst 6.0 (https://www.metaboanalyst.ca/).

### 2.6 Validation of key targets and signaling pathways

#### 2.6.1 Detection of lipid metabolism indicators

Lipid metabolism-related indices, including TG, TC, HDL-C, LDL-C, OxLDL, and NEFA, were assessed based on the KEGG enrichment analysis pathway.

#### 2.6.2 Detection of mRNA expression by QRT-PCR method

Total RNA (10–20 mg) extracted from mice kidney tissue was reverse transcribed to synthesize cDNA, which was subsequently used for QRT-PCR. The QRT-PCR procedure followed a two-step method: 95°C for 30 s for pre-denaturation, followed by 95°C for 10 s for denaturation, 60°C for annealing, and a total of 40 cycles. Data were analyzed using the 2^−ΔΔCT^ method, and primer sequences for QRT-PCR are provided in Supplementary Table S2.

#### 2.6.3 WB detection of protein expression

Approximately 50 mg of kidney tissue was weighed, homogenized in pre-cooled RIPA buffer containing protease and phosphatase inhibitors (1:10), and then centrifuged to obtain the supernatant, which was used for total kidney tissue protein extraction. Protein concentrations were quantified using the BCA method. The samples were heated in a boiling water bath at 100°C for 10 min to ensure complete protein denaturation prior to gel electrophoresis. Proteins were transferred to a PVDF membrane, which was blocked with 5% skimmed milk powder. Primary antibodies against PI3K (1:1,000), AKT (1:1,000), ERK1/2 (1:1,000), and β-actin (1:5,000) were applied and incubated overnight at 4°C. After washing the membrane with TBST (15 min, 3 times), the HRP-conjugated secondary antibody (1:2000) was incubated at room temperature for 1 h. Protein bands were visualized using ECL luminescence, and protein expression was quantified using ImageJ software.

### 2.7 Statistical methods

Metabolomics data were processed using the Majorbio Cloud Platform. Other data were statistically analyzed and plotted using GraphPad Prism 9.5.0 software. The data are presented as mean ± standard deviation (mean ± SD). Depending on the specific data analysis, various statistical methods were applied, such as unpaired two - tailed t - test, Kruskal - Wallis H test, one - way analysis of variance test, and non - parametric test. A p - value <0.05 was considered statistically significant.

## 3 Results

### 3.1 Evaluation of pharmacodynamic effects of HLB intervention in DN mice

#### 3.1.1 Effect of HLB on glucose metabolism

**FIGURE 1:**
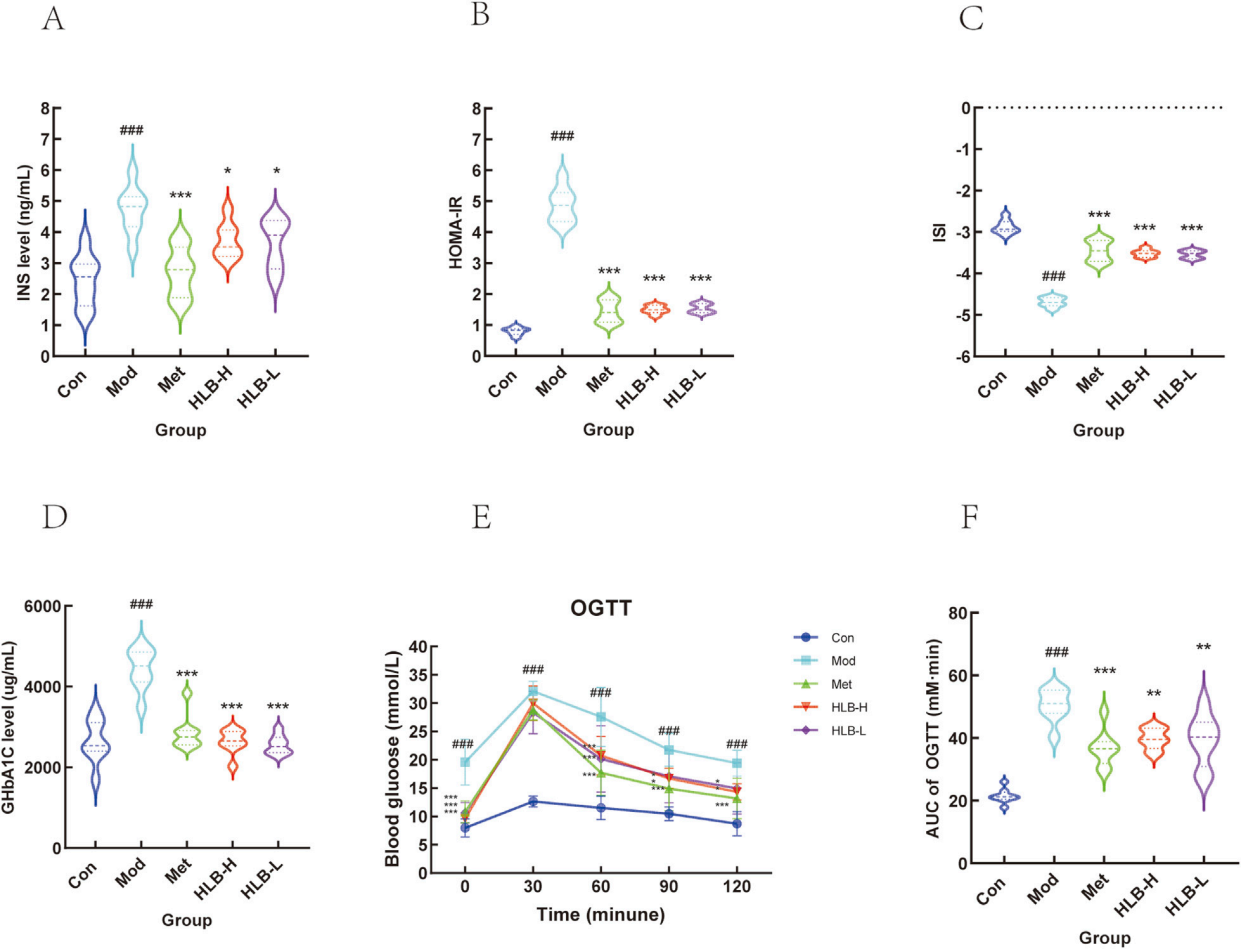
Effect of HLB on insulin levels in mice with oral glucose tolerance experiment **(A)** Serum insulin level. **(B)** Insulin resistance index. **(C)** Insulin sensitivity index. **(D)** Glycosylated haemoglobin level. **(E)** HLB on oral glucose tolerance experiment. **(F)** Area under the blood glucose curve of each group of mice.

Compared to the Con group, mice in the Mod group exhibited significantly elevated INS and IR (p < 0.001), along with significantly reduced ISI, indicating the onset of insulin resistance and diminished insulin sensitivity in the model mice. In contrast, the Met, HLB-H, and HLB-L treatment groups showed significantly lower INS and IR levels and significantly higher ISI values (p < 0.001 for Met, p < 0.05 and p < 0.001 for HLB-H, p < 0.05 and p < 0.001 for HLB-L), suggesting that HLB effectively enhances insulin sensitivity and ameliorates insulin resistance in the context of T2DM (Figures 1A–C). Additionally, serum glycated hemoglobin (HbA1c) levels were markedly elevated in the Mod group compared to the Con group (p < 0.001). However, treatment with Met, HLB-H, and HLB-L significantly reduced HbA1c levels (p < 0.001), indicating that HLB can mitigate the elevation of HbA1c in the T2DM model (Figure 1D). The results of the OGTT and AUC analysis revealed impaired glucose tolerance in the Mod group (Figures 1E, F), with a significantly higher AUC for OGTT blood glucose fluctuations in the Mod group compared to the Con group (p < 0.001). Following treatment, the AUC was significantly reduced in the Met, HLB-H, and HLB-L groups (p < 0.001, p < 0.01, p < 0.01), demonstrating that HLB treatment led to marked improvements in both glucose metabolism and insulin resistance after 4 weeks.

#### 3.1.2 Urine indicator test results

**FIGURE 2:**
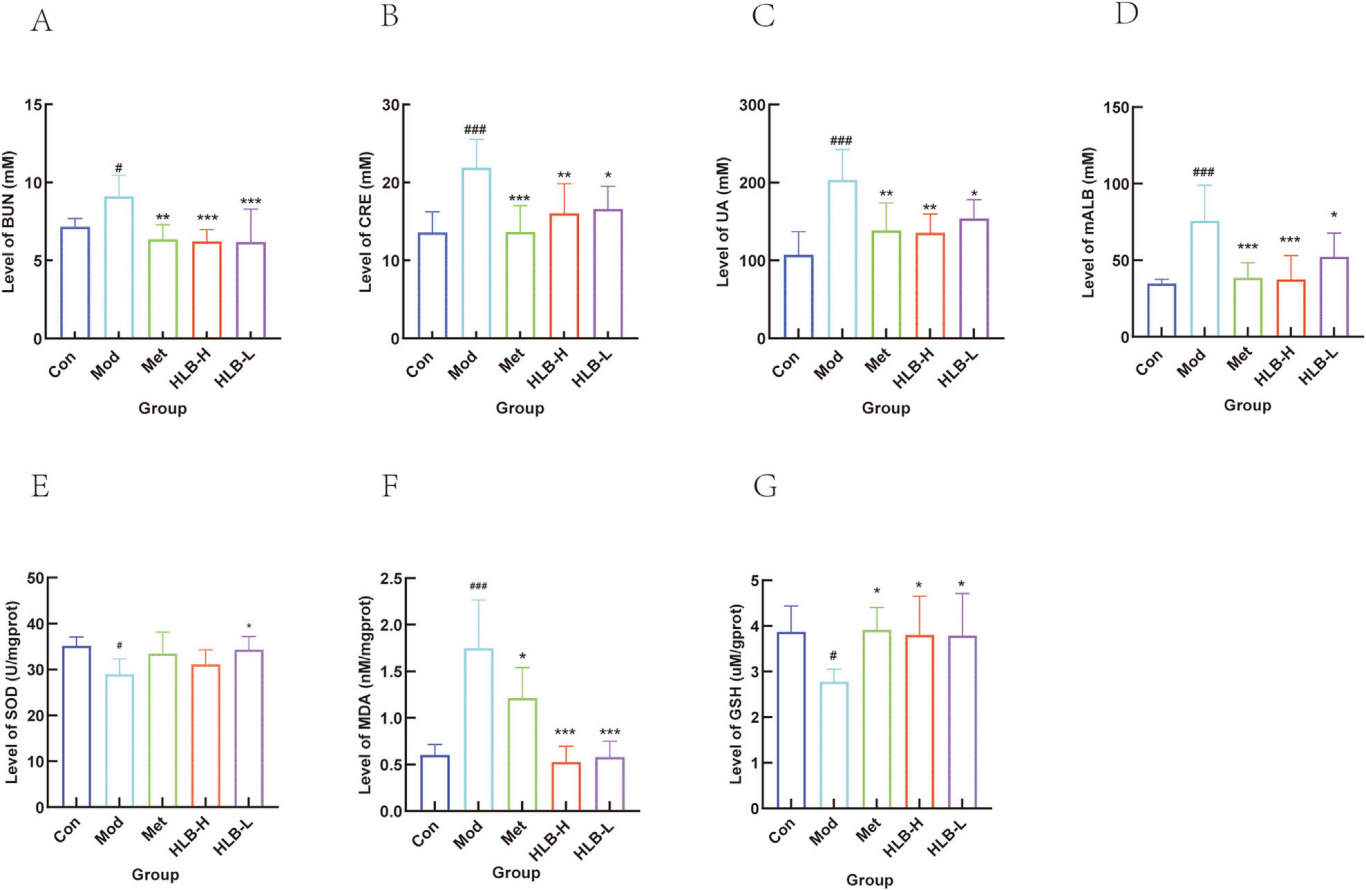
Effect of HLB on serum renal function indices in mice. **(A)** Serum BUN level. **(B)** Serum CRE level. **(C)** Serum UA level. **(D)** Serum mALB level. **(E)** Level of SOD. **(F)** Level of MDA. **(G)** Level of GSH.

Regarding renal function, the levels of BUN, CRE, UA, and mALB were significantly elevated in the Mod group compared to the Con group (p < 0.05, p < 0.001, p < 0.001, p < 0.001, p < 0.001), indicating renal impairment in the model mice. Treatment with Met resulted in significant reductions in BUN, CRE, UA, and mALB levels (p < 0.01, p < 0.001, p < 0.01, p < 0.001) (Figures 2A–D). Similarly, significant improvements were observed in the HLB-treated groups compared to the Mod group. The HLB-H group notably reduced serum BUN, CRE, UA, and mALB levels (p < 0.001, p < 0.01, p < 0.01, p < 0.001), while the HLB-L group also demonstrated significant reductions in these markers (p < 0.001, p < 0.05, p < 0.05, p < 0.05). These results suggest that HLB administration effectively alleviates renal dysfunction in a diabetic state, as indicated by improvements in BUN, CRE, UA, and mALB levels when compared to the Mod group. Compared with the Met group (p < 0.01), the downregulation of BUN levels in the HLB - H and HLB - L groups was more significant. The significance of CRE levels in the Met group (p < 0.001) was more obvious than that in the HLB - H group (p < 0.01) and the HLB - L group (p < 0.05). Regarding the levels of UA and mALB, the significance in the Met group and the HLB - H group was more obvious compared with the HLB - L group (p < 0.05), with p < 0.01 and p < 0.001 respectively. The above - mentioned indicators suggest that HLB can, to a certain extent, improve renal function impairment in a diabetic state.

#### 3.1.3 Results of oxidative stress indicators in renal tissue

SOD (antioxidant or active substance), MDA (free radical reaction product), and GSH (low molecular scavenger) serve as indicators of oxidative stress levels within an organism. In the Mod group, both SOD and GSH levels were significantly reduced (p < 0.05), while MDA levels were significantly elevated (p < 0.001) compared to the Con group, indicating the presence of oxidative stress in the model mice. Treatment with Met resulted in a significant increase in GSH levels (p < 0.05), an increase in SOD activity (without a statistically significant difference), and a marked reduction in MDA levels (p < 0.05). HLB administration also enhanced SOD activity (HLB-H, no significant difference; HLB-L, p < 0.05), increased GSH levels (p < 0.05), and significantly reduced MDA levels (p < 0.001). These results suggest that HLB may mitigate oxidative stress in DN by upregulating the antioxidant defense system, reducing free radical byproducts, and alleviating oxidative damage (Figures 2E–G).

#### 3.1.4 Histopathological examination of the kidney

**FIGURE 3:**
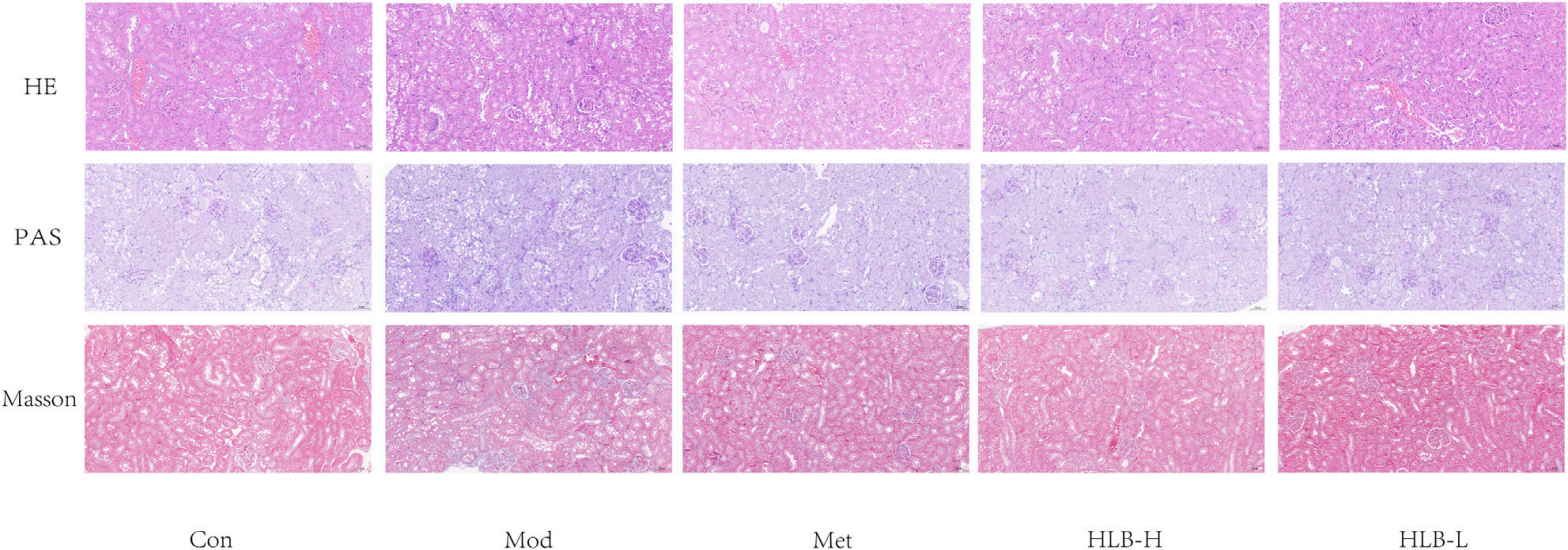
HLB ameliorated the pathological abnormalities of renal tissue as observed by light microscopy (HE, PAS and Masson staining, 20×).

Figure 3 presents the histopathological findings from HE, PAS, and Masson staining of renal tissue.

HE staining revealed that the renal tissue structure in the Con group was well-preserved and intact, with no observable abnormalities. In contrast, the Mod group exhibited significant pathological changes, including increased glomerular volume, dilation of the renal capsule, and hypertrophy of renal tubular epithelial cells, which were accompanied by vacuolar degeneration. Following treatment, the Met, HLB-H, and HLB-L groups showed notable reductions in glomerular damage and a slowing of renal tubular dilation, suggesting that these treatments mitigated the structural damage caused by the diabetic condition.

PAS staining results demonstrated that, in the Con group, renal tissue maintained normal morphology. However, in the Mod group, the kidneys exhibited thickening of the glomerular basement membrane, hyperplasia of the mesangial matrix, and glomerulosclerosis. Treatment with Met, HLB-H, and HLB-L slowed the proliferation of the glomerular basement membrane and mesangial matrix, as well as reduced glomerulosclerosis, indicating that these interventions helped attenuate glomerular injury in the DN model.

Masson staining results showed that in the Con group, there was minimal collagen fiber deposition in the renal tissue, which appeared as loose connective tissue, with no signs of renal tubule fibrosis. In contrast, the Mod group exhibited significant atrophy of renal tubular epithelial cells, pronounced vacuolar degeneration, and a marked increase in interstitial collagen fibers, reflecting severe renal fibrosis. In the treatment groups (Met, HLB-H, and HLB-L), renal tubular vacuolar degeneration was reduced, and the extent of renal fibrosis was significantly alleviated, demonstrating that these treatments effectively reduced fibrosis and tubular damage.

Collectively, the histopathological results indicate that both Met and HLB interventions improved renal morphology in DN model mice, with reduced tubular atrophy, glycogen accumulation, and collagen fiber proliferation. These results suggest that Met and HLB can ameliorate renal dysfunction and structural damage in diabetic nephropathy.

### 3.2 Chemical composition characterization

**FIGURE 4:**
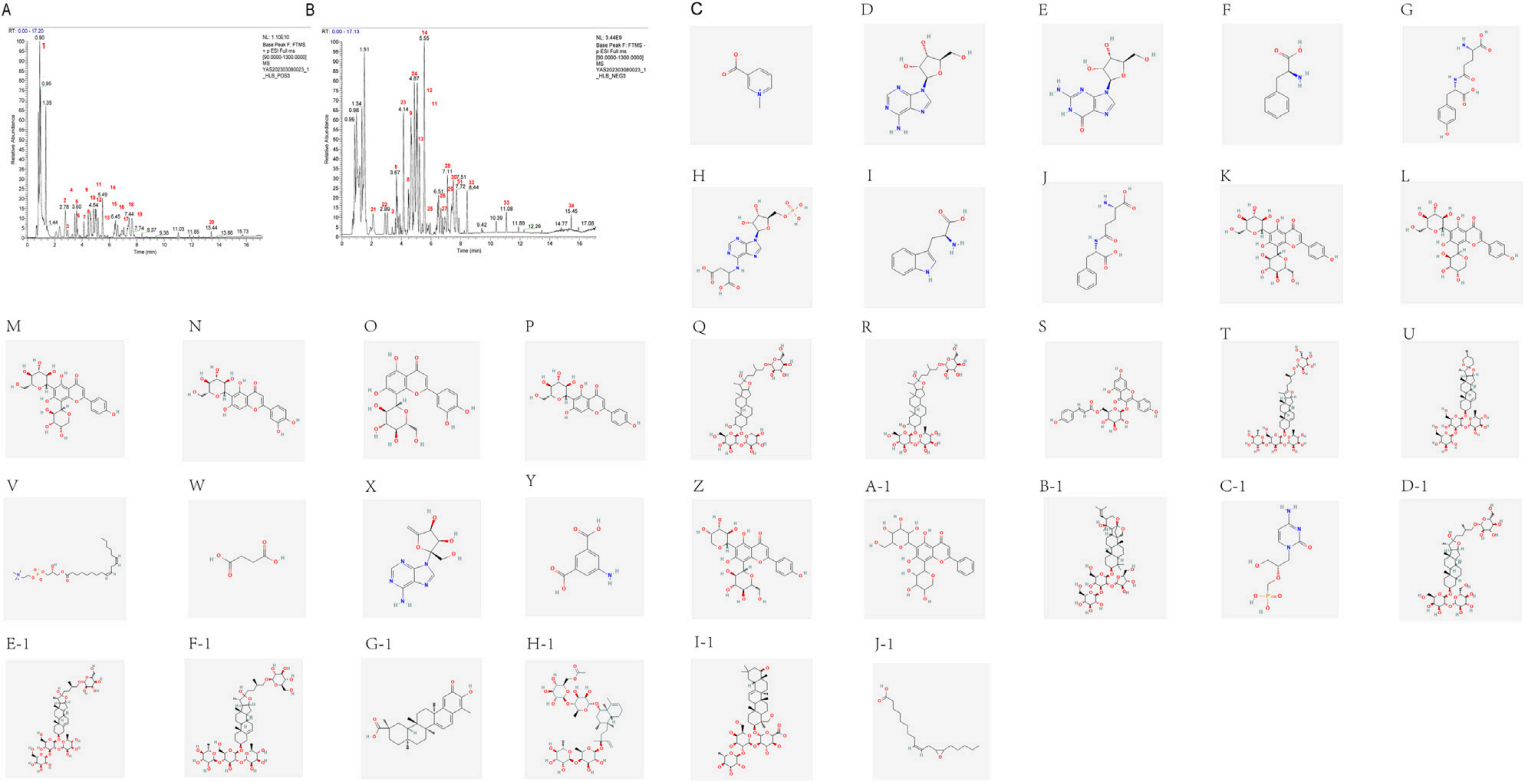
UHPLC-HRMS total ion curent chromatogram of HLB **(A)** BPC plot-labelled peaks in HLB positive ion mode. **(B)** BPC plot-labelled peaks in HLB negative ion mode. Numbers represent the following compounds: 1. Trigonelline, 2. Adenosine, 3. Guanosine, 4. Phenylalanine, 5. gamma-Glutamyltyrosine, 6. Adenylsuccinic acid, 7. L-Tryptophan, 8. gamma-Glutamylphenylalanine, 9. Vicenin-2, 10. Neoschaftoside, 11. Schaftoside, 12. Isoorientin, 13. Orientin, 14. Isovitexin, 15. NCGC00385394-01, 16. Trigoneoside Xb, 17. Tiliroside, 18. Pseudoprotodioscin, 19. Gracillin, 20.1-Linoleoyl-sn-glycero-3-phosphorylcholine, 21. Succinic acid, 22. Decoyinine, 23.5-Aminoisophthalic acid, 24. Isoschaftoside, 25.5,7-dihydroxy-2-phenyl-6-[3,4,5-trihydroxy-6-(hydroxymethyl)oxan-2-yl]-8-(3,4,5-trihydroxyoxan-2-yl)-4H-chromen-4-one, 26. Bacopaside II, 27. Cidofovir, 28. Timosaponin b ii, 29. Protogracillin, 30. Protodioscin, 31. (3S)-5-((1S,2R,4S,4aR, 8aR)-4-((4-O-(6-O-Acetyl-.beta.-D-glucopyranosyl)-6-deoxy-.alpha.-L-mannopyranosyl)oxy)-1,2,4a,5-tetramethyl-1,2,3,4,4a,7,8,8a-octahydronaphthalen-1-yl)-3-methylpent-1-en-3-yl 6-deoxy-4-O-(6-deoxy-.alpha.-L-mannopyranosyl)-.beta.-D-galactopyranoside, 32. Celastrol, 33. MCULE-7407861812, 34.12 (13)-EpOME.

The chemical components in the aqueous extract of HLB were identified via UHPLC-HRMS, revealing a total of 34 compounds. The base peak chromatograms (BPC) in both positive and negative ion modes, along with the structural diagrams of the 34 components, are shown in Figure 4. The detailed information of each compound is presented in Supplementary Table S3.

### 3.3 Network pharmacology results

#### 3.3.1 Target screening

**FIGURE 5:**
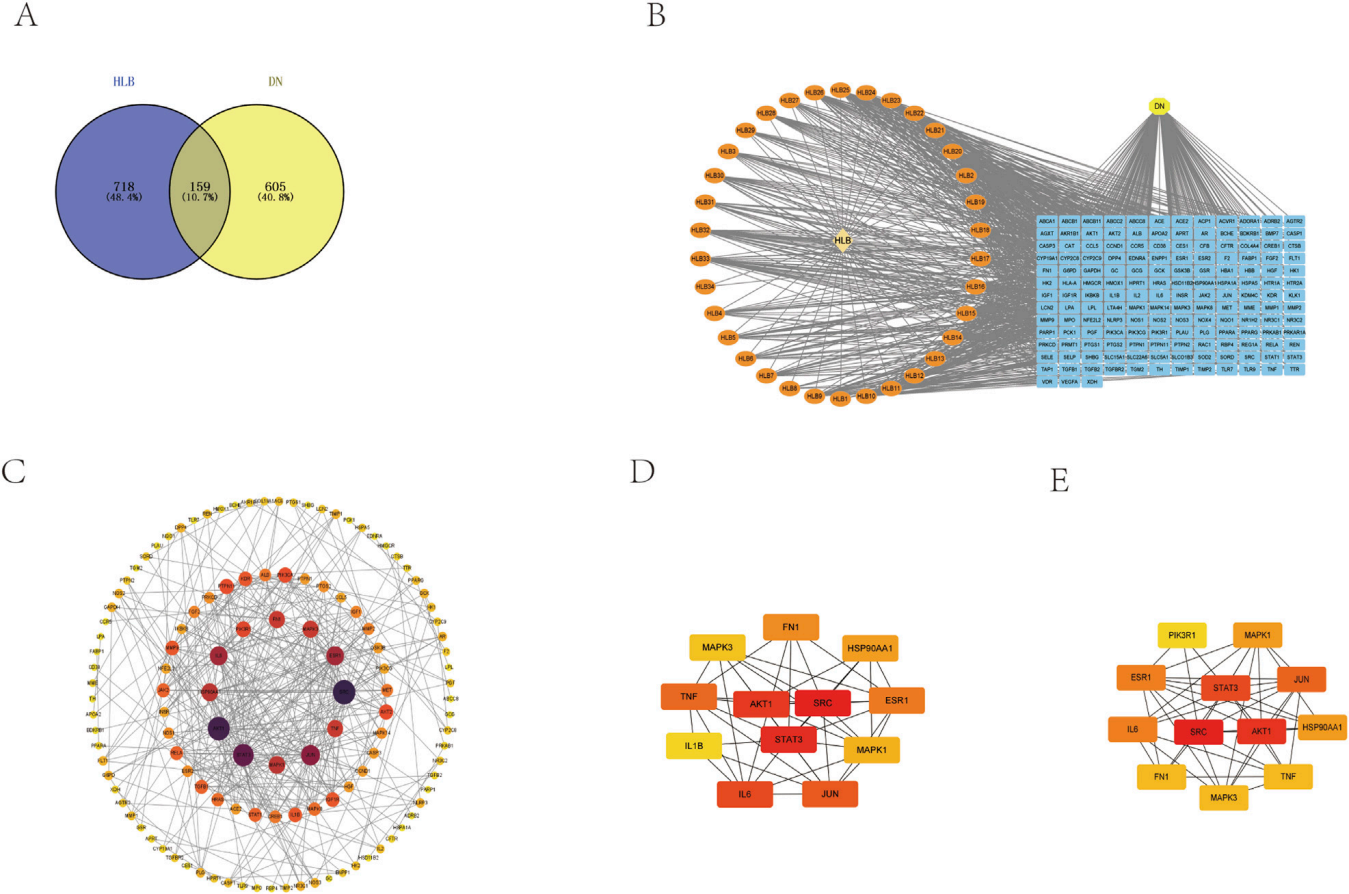
**(A)** Drug-disease target Venny diagram intersection. **(B)** HLB chemical composition-common target-disease CTD diagram. **(C)** PPI network construction. **(D)** Screening of core genes based on closeness. **(E)** Screening of core genes based on degree.

UHPLC-HRMS analysis of these compounds, followed by target prediction through the TCMSP, ETCM, Pharm Mapper, and Swiss Target Prediction databases, identified 877 unique targets after removing duplicates. Screening of disease-related databases revealed 764 targets associated with DN. The intersection of drug and disease targets yielded 159 common targets (Figure 5A).

#### 3.3.2 Construction and analysis of CTD interaction networks

These drug-disease target intersections were analyzed using Cytoscape to construct the CTD network (Figure 5B).

#### 3.3.3 PPI network construction and core gene screening

The 159 intersecting targets were further examined using the STRING database to build a PPI network (Figure 5C), comprising 159 target proteins and 395 interaction edges. The network displayed an average target degree of 4.97 and a local clustering coefficient of 0.514, indicating a highly significant PPI enrichment (p-value <1.0e-16). The resulting PPI network, visualized using Cytoscape 3.9.1 based on the Degree parameter, highlighted 12 key genes—MAPK3, MAPK1, FN1, JUN, IL6, PIK3R1, TNF, SRC, HSP90AA1, ESR1, STAT3, and AKT1—as central to the network. These genes were prioritized through Cytohubba using degree and closeness parameters, with higher-scoring targets displayed in a network format (Figures 5D, E).

#### 3.3.4 GO and KEGG enrichment analyses

**FIGURE 6:**
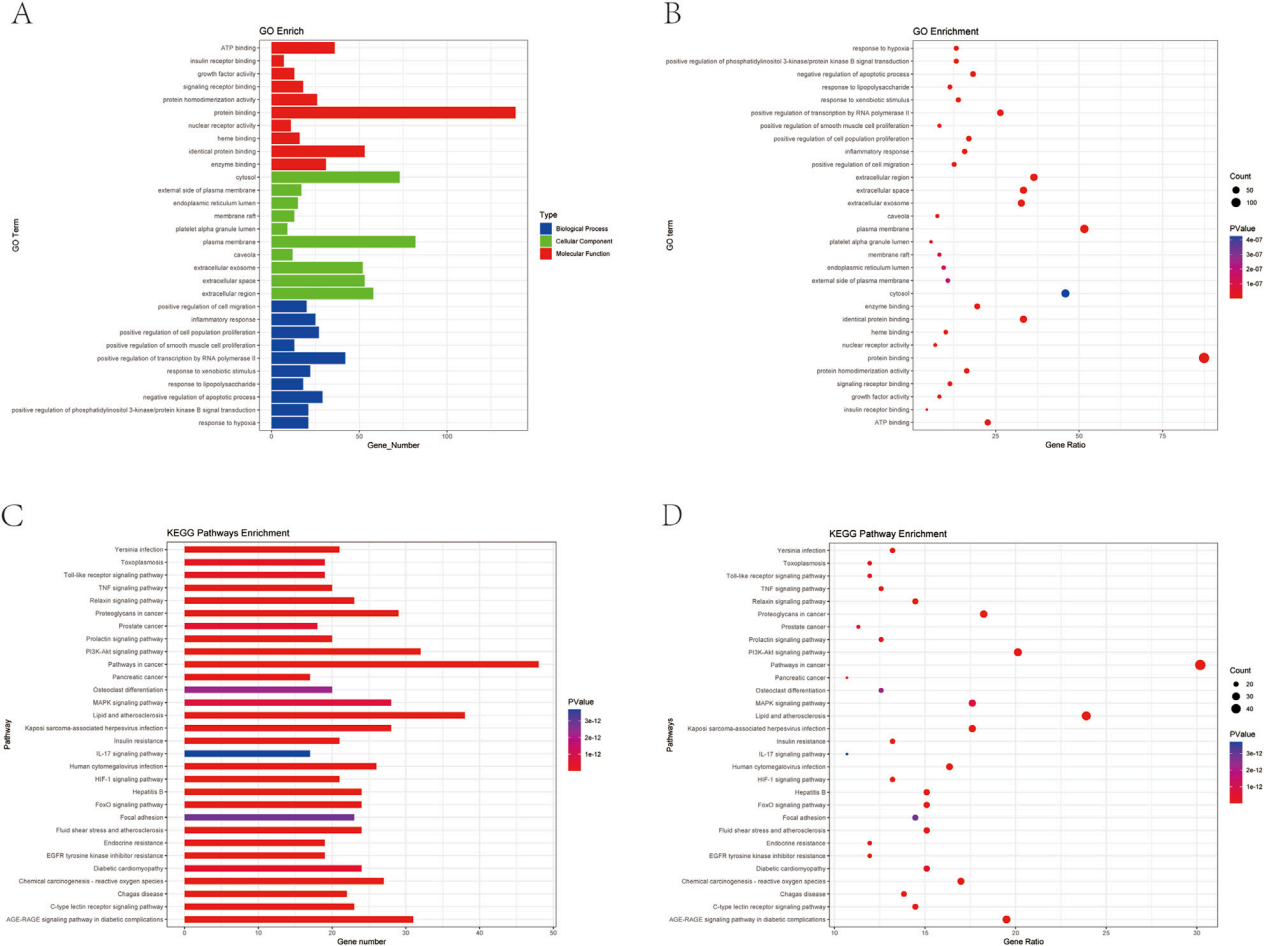
Plot of GO *versus* KEGG enrichment analysis **(A)** Target-enriched GO pathway histogram. **(B)** Target-enriched GO pathway bubble plot. **(C)** Target protein-enriched KEGG pathway histogram. **(D)** Target protein-enriched KEGG pathway bubble plot.

The 159 intersecting genes were input into the DAVID database (https://david.ncifcrf.gov/) for functional annotation. GO enrichment analysis revealed 708 terms for BP, 69 terms for CC, and 157 terms for MF. The top 10 enriched terms in each category are visualized in bubble plots (Figures 6A, B), with key terms including positive regulation of transcription by RNA polymerase II (BP), plasma membrane (CC), and protein binding (MF) as the most significant. Additionally, KEGG pathway analysis identified 174 relevant terms associated with the 159 intersecting genes. This suggests that HLB may exert its therapeutic effects on DN through the modulation of multiple signaling pathways. A selection of 30 important pathways is illustrated in the bar graph (Figures 6C, D). Among these, pathways such as Lipid and atherosclerosis (hsa05417), the PI3K-Akt signaling pathway (hsa04151), and the AGE-RAGE signaling pathway in diabetic complications (hsa04933) were enriched by five key target genes (MAPK1, MAPK3, AKT1, PIK3R1, IL6). Notably, the PI3K-Akt signaling pathway, a critical pathway in diabetes, was enriched with additional key targets, including IL6, HSP90AA1, FN1, MAPK1, AKT1, PIK3R1, and MAPK3. These diabetes-related pathways and their associated targets were selected for further validation.

### 3.4 Untargeted metabolomic results

#### 3.4.1 Screening for potential differential metabolites

**FIGURE 7:**
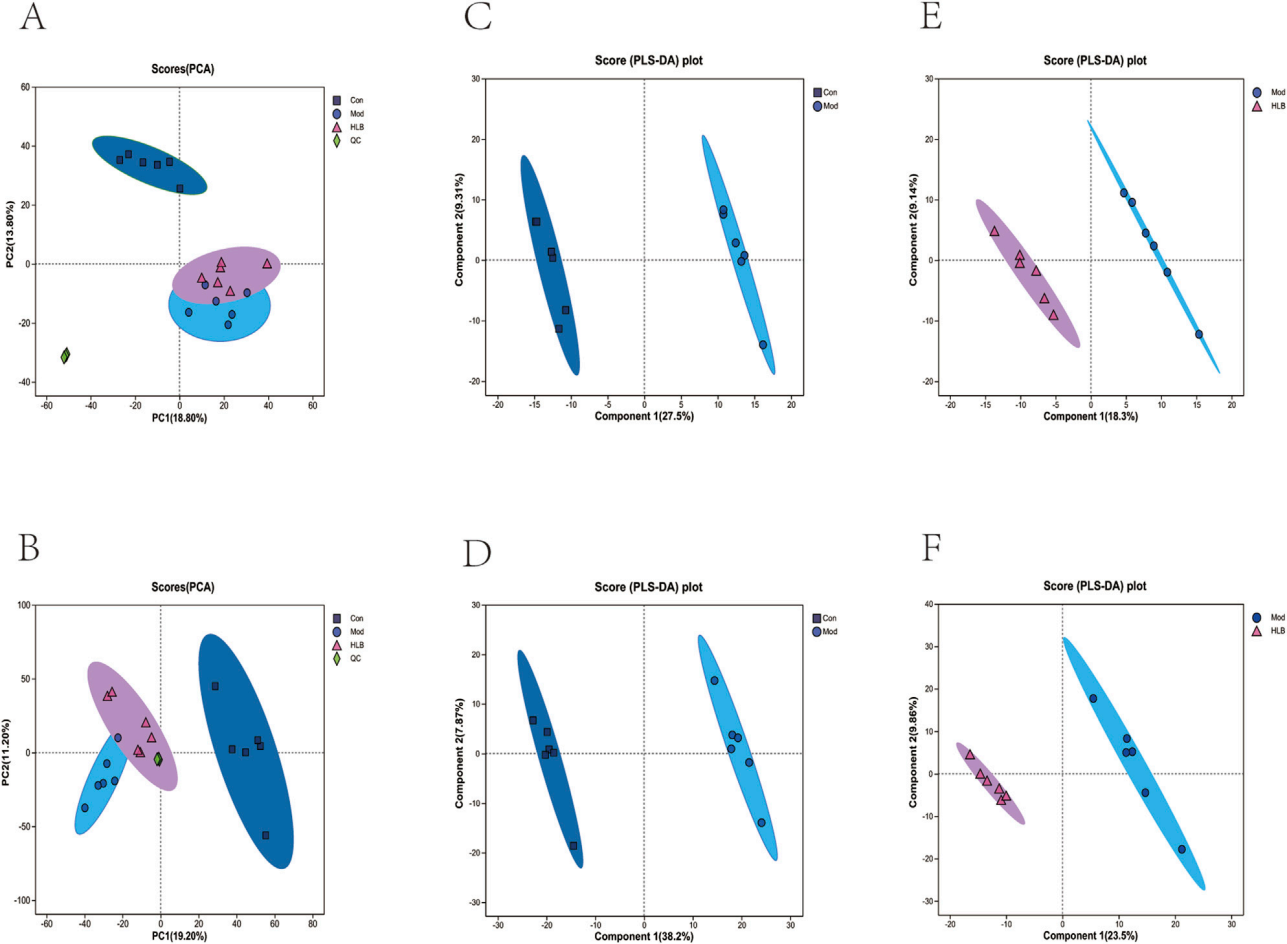
Effects of HLB on metabolic changes in DN mice **(A)** Plot of PCA scores of different metabolites in positive ion mode in each group. **(B)** Plot of PCA scores of different metabolites in negative ion mode in each group. **(C)** Plot of PLS-DA scores of different metabolites in positive ion mode in the Con and Mod groups. **(D)** Plot of PLS-DA scores of different metabolites in negative ion mode in the Con and Mod groups. **(E)** Plot of PLS-DA scores of metabolites in positive ion mode in the Mod group and the HLB group DA scoring plot. **(F)** PLS-DA scoring plot of metabolites in negative ion mode in Mod group vs. HLB group.

**FIGURE 8:**
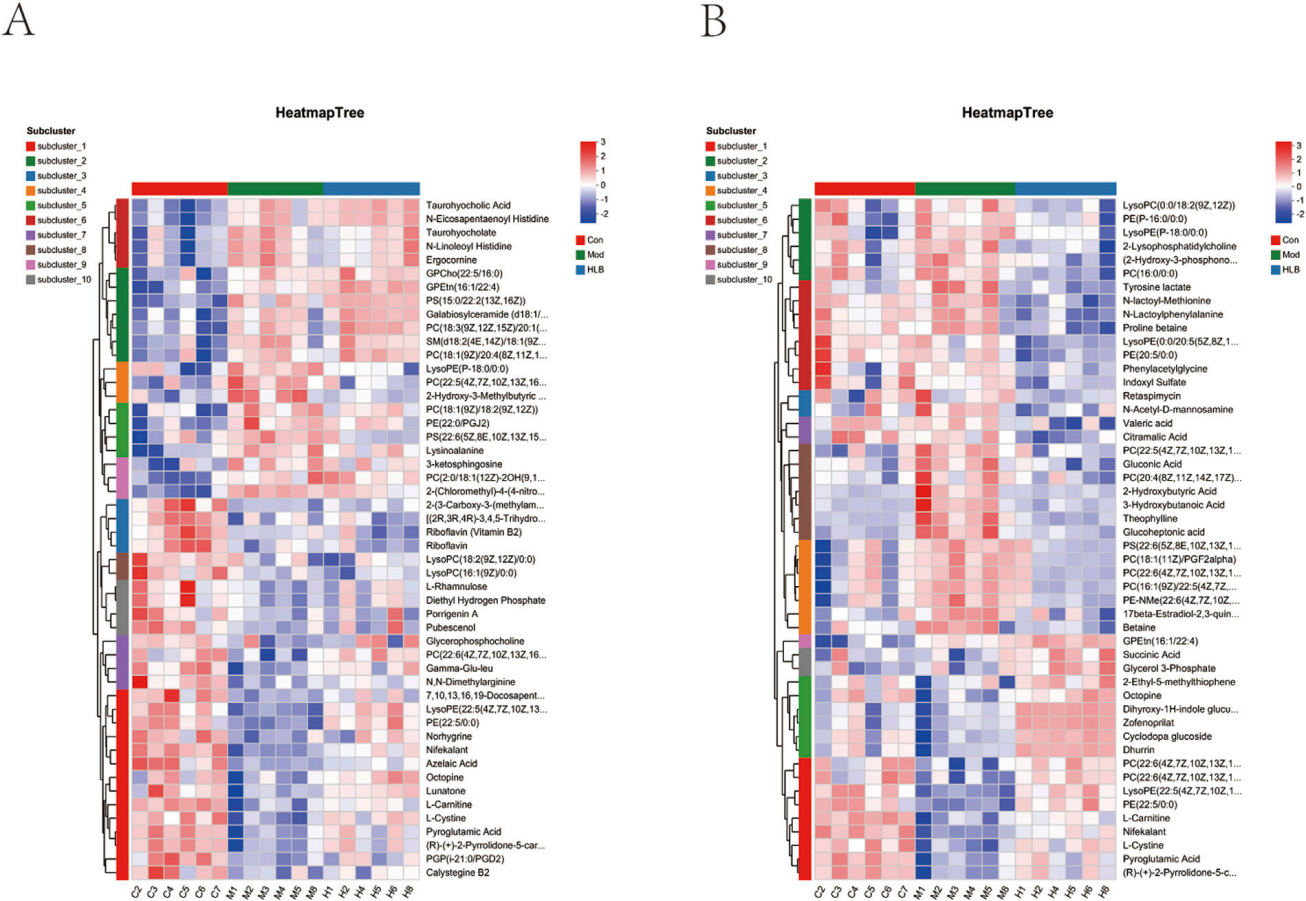
Differential metabolite heatmap **(A)** differential metabolite heatmap between con and mod groups. **(B)** Differential metabolite heatmap between mod and HLB groups.

**FIGURE 9:**
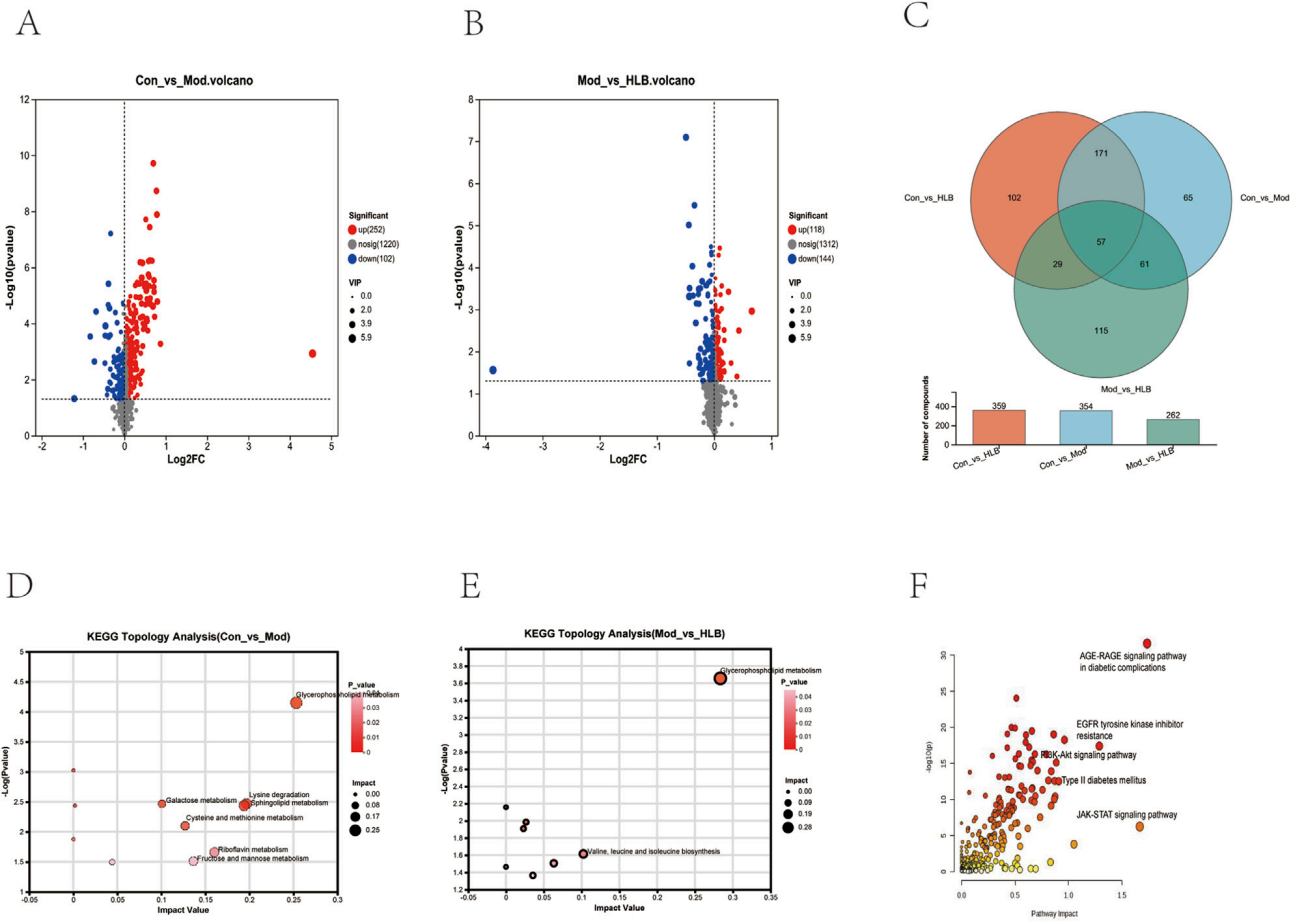
**(A)** Volcano plot of Con_vs._Mod differential metabolites. **(B)** Volcano plot of Mod_ vs._HLB differential metabolites. **(C)** Venn diagram for overlap of different groups of metabolites. **(D)** Con_vs._Mod metabolic pathway analysis results. **(E)** Mod_vs._HLB metabolic pathway analysis results. **(F)** Metabolic pathway integration results.

In this study, the metabolic profiles of the Con, Mod, and HLB groups were analyzed using the Majorbio cloud platform. As depicted in the unsupervised PCA score plot (Figures 7A, B), the QC samples were tightly clustered, indicating high equipment stability and reinforcing the reliability of both the experimental methodology and the data obtained. The PCA results demonstrated clear separation between the Con and Mod groups, suggesting that this analytical approach can effectively distinguish HLB-treated DN mice from untreated counterparts. PLS-DA analysis revealed significant differences in the metabolic profiles of the Con and Mod groups, confirming the successful replication of the DN mice model and the induction of notable metabolic alterations. Similarly, significant metabolic differences between the Mod and HLB groups were observed, indicating that 4 weeks of HLB treatment profoundly impacted the metabolic profiles of DN mice. Differential metabolites were screened using p < 0.05 and VIP >1.0 criteria. Under both positive and negative ion modes, 175 and 179 differential metabolites were identified in the Con and Mod groups, respectively. In comparison, 126 and 136 differential metabolites were detected in the Mod and HLB groups. To visualize the trends of differential metabolites across groups, metabolite clustering analysis was performed, resulting in a clustering heat map. As shown in Figures 8A, B, each column represents a sample, and each row corresponds to a metabolite, with color intensity reflecting the relative expression levels of metabolites within each sample group. The dendrograms on the left and right indicate metabolite clustering, while those at the top and bottom represent sample clustering. The results revealed that differential metabolite clusters within the same group exhibited consistent expression patterns. Cluster analysis further confirmed that the metabolites in each group were distinctly separated, with a clear differentiation trend observed between the groups. The volcano plots in dual-ion mode (Figures 9A, B) visually represent the upregulation and downregulation of differential metabolites, where each dot corresponds to a specific metabolite, and the size of the dot indicates the VIP value. Red dots represent significantly upregulated metabolites, blue dots represent significantly downregulated metabolites, and grey dots denote metabolites with no significant differentiation. A total of 354 differential metabolites were detected in the Mod_vs._Con comparison (Supplementary Table S4), with 252 upregulated and 102 downregulated. Additionally, 262 differential metabolites were identified in the HLB_vs._Mod comparison (Supplementary Table S5), of which 118 were upregulated and 144 downregulated. To facilitate a more visual comparison of the metabolite changes at each experimental stage, Venn diagrams were used to illustrate the shared and unique metabolites between the different groups (Figure 9C). Based on the analysis of the 262 metabolites differing between the Mod and HLB groups, 61 metabolites (Supplementary Table S6) were identified as significantly altered in Mod mice but not in Con mice, including D-Galactosamine, Galactosylglycerol, L-Acetylcarnitine, and other monosaccharides, amino acids, and phospholipids. These metabolites may serve as potential biomarkers for DN improvement following HLB treatment.

#### 3.4.2 Metabolic pathway analysis

The identified renal differential metabolites were subjected to metabolic pathway topology analysis, with pathways selected based on the criteria of p-value <0.05 and Impact value >0.1. As shown in Figures 9D, E, the Mod group significantly affected seven pathways, including Glycerophospholipid metabolism, Lysine degradation, Fructose and mannose metabolism, and Galactose metabolism, compared to the Con group. In contrast, the HLB group significantly influenced two pathways, including Glycerophospholipid metabolism and Valine, leucine, and isoleucine biosynthesis, when compared to the Mod group.

### 3.5 Target and signaling pathway validation

Metabolic pathway correlation analysis, conducted using the MetaboAnalyst 6.0 combined pathway analysis module, was performed on the predicted 159 targets and 61 differential metabolites (Figure 9F). The screening criteria were p < 0.05 and Impact >0.1, which revealed that the AGE-RAGE signaling pathway in diabetic complications, Type II diabetes mellitus, Insulin signaling pathway, and PI3K-Akt signaling pathway were significantly elevated. The key protein targets MAPK1, MAPK3, AKT1, PI3K, and ERK were identified as playing important roles across multiple pathways, suggesting that the PI3K-Akt-ERK signaling pathway plays a pivotal role in the improvement of DN by HLB.

#### 3.5.1 Validation of lipid metabolism indicators

**FIGURE 10:**
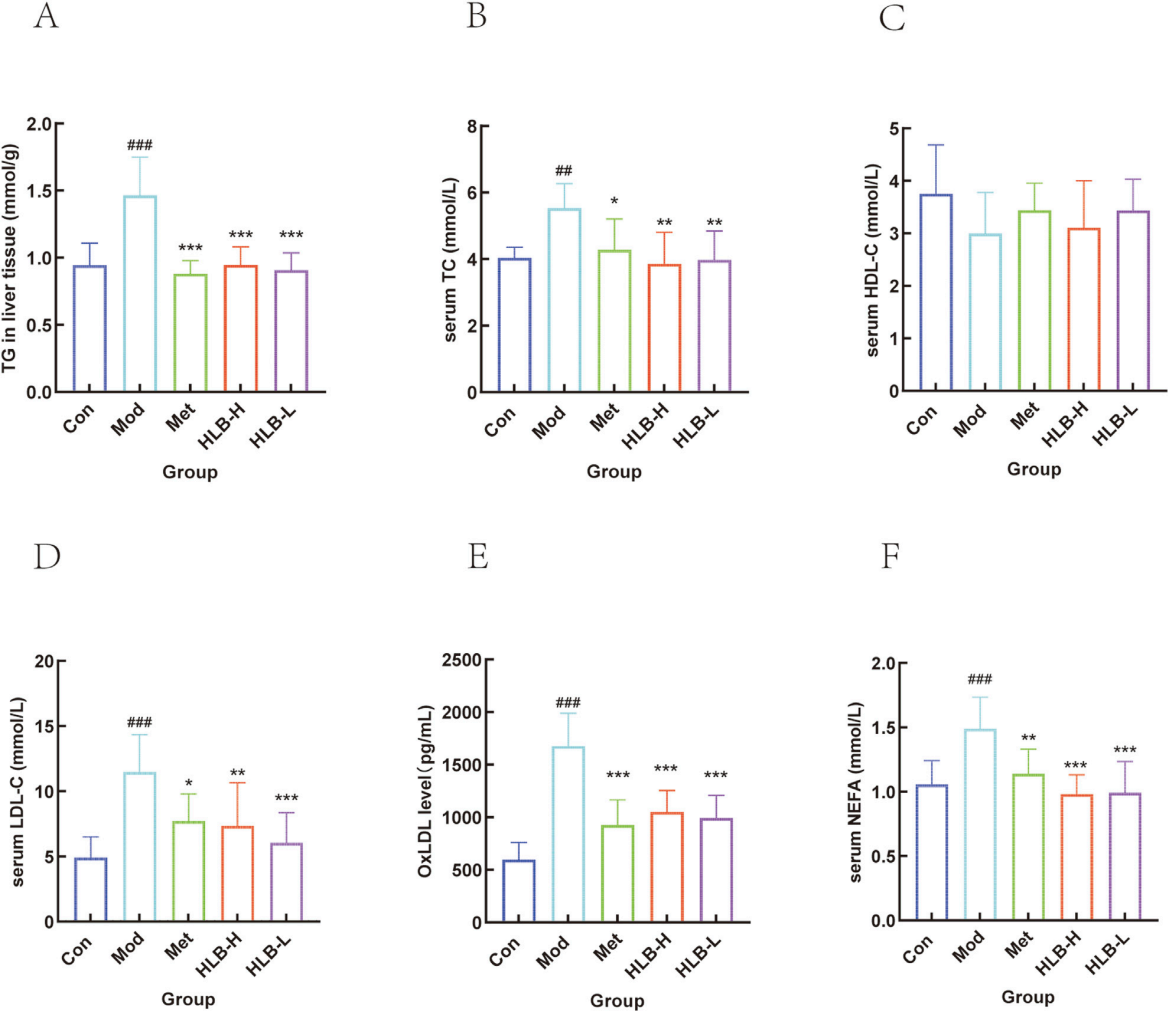
Effect of HLB on lipid metabolism related indexes in mice **(A)** Level of TG. **(B)** Level of TC. **(C)** Level of HDL-C. **(D)** Level of LDL-C. **(E)** Level of OxLDL. **(F)** Level of NEFA.

KEGG enrichment analysis further supported the finding that HLB may improve DN through lipid and atherosclerotic pathways. To investigate this, lipid-related indices (TG, TC, LDL-C, HDL-C, OxLDL, and NEFA) were measured to evaluate lipid metabolism. As shown in Figures 10A-F, the results showed that in the Mod group, TG, LDL-C, OxLDL, and NEFA levels were significantly elevated compared to the Con group (p < 0.001), while TC was also significantly higher (p < 0.01), and HDL-C levels were reduced without significant differences. These results indicated that the DN mice model used in this study exhibited significant lipid metabolism abnormalities. Following 4 weeks of drug intervention, the serum levels of TG, OxLDL (p < 0.001), TC, LDL-C (p < 0.05), and NEFA (p < 0.01) in the Met group were significantly reduced, with HDL-C levels elevated but without significant differences. In comparison to the Mod group, the HLB-H and HLB-L groups showed significantly lower levels of TG, OxLDL, and NEFA (p < 0.001), significantly lower levels of TC (p < 0.01), and LDL-C (p < 0.01; p < 0.001), while HDL-C levels were elevated but did not significantly differ. These results suggest that HLB has a beneficial effect on lipid metabolism disorders.

#### 3.5.2 QRT-PCR to detect mRNA expression

To further investigate whether HLB administration could delay the progression of renal impairment, the mRNA levels of key genes such as *MAPK1*, *MAPK3*, *AKT1*, *PI3K*, *HSP90AA1*, and *FN1* were assessed. QRT-PCR amplification was used to quantitatively assess the mRNA levels of these genes after drug intervention.

**FIGURE 11:**
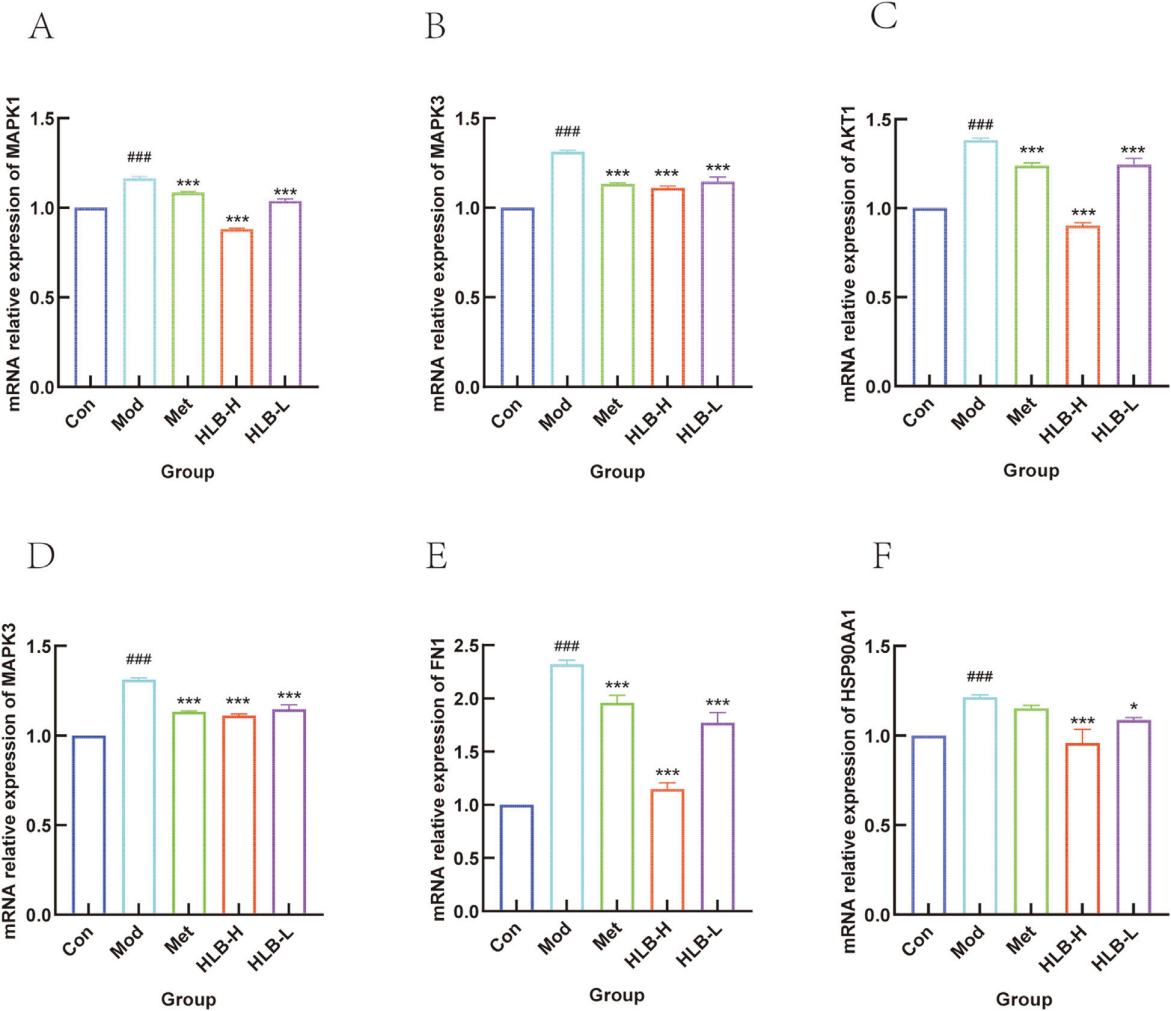
**(A)** *MAPK1* mRNA level. **(B)** *MAPK3* mRNA level. **(C)** *AKT1* mRNA level. **(D)** *PI3K* mRNA level. **(E)** *HSP90AA1* mRNA level. **(F)** *FN1* mRNA level.

As shown in Figures 11A–F, the mRNA levels of *MAPK1*, *MAPK3*, *AKT1*, *PI3K*, *HSP90AA1*, and *FN1* were significantly elevated in the kidneys of the Mod group compared to the Con group (p < 0.001). However, after drug administration, there was a significant decreasing trend in these gene expression levels in the HLB-H and Met group (p < 0.001, except for *HSP90AA1* intervened by metformin, which did not show significance.), and the HLB-L group also showed significant reductions in *MAPK1*, *MAPK3*, *AKT1*, *PI3K*, *HSP90AA1*, and *FN1* mRNA levels (p < 0.001, p < 0.001, p < 0.001, p < 0.01, p < 0.001, p < 0.05, respectively). The experimental results showed that, after the administration of HLB, the mRNA levels of *MAPK1*, *MAPK3*, *AKT1*, *PI3K*, *HSP90AA1* and *FN1* regulated by HLB might be superior to those regulated by metformin to a certain extent. This result also provides more basic research for further exploring the potential value of HLB.

#### 3.5.3 WB detection of protein expression

**FIGURE 12:**
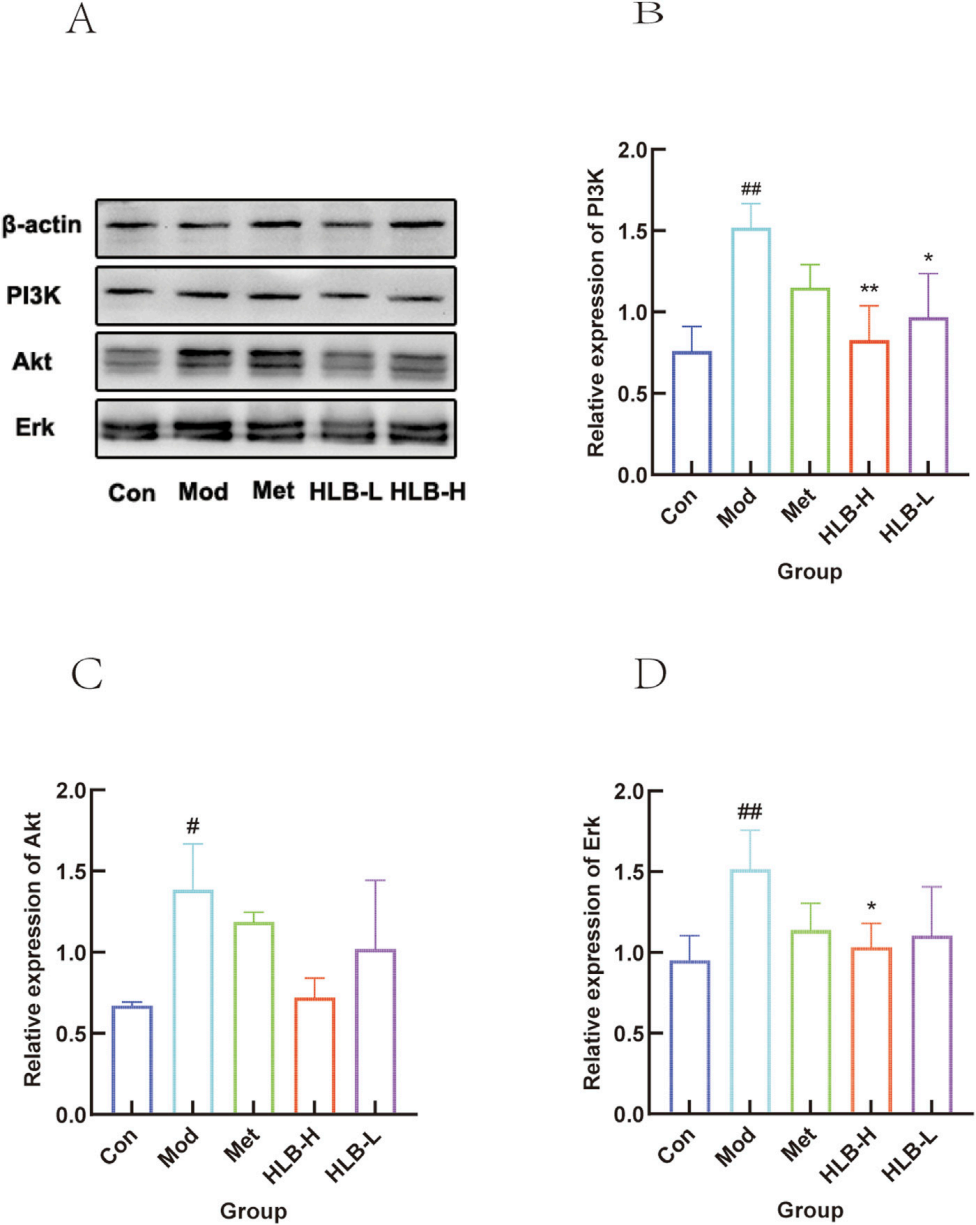
WB detection of HLB regulation of central gene protein expression in DN mice **(A)** Validation of HLB treatment for central gene DN. **(B)** Histogram of PI3K protein expression. **(C)** Histogram of Akt protein expression. **(D)** Histogram of Erk protein expression.

WB analysis was performed to assess the impact of HLB treatment on the expression profiles of PI3K, AKT, ERK in renal tissue (Figure 12). The analysis revealed a significant increase in the expression levels in the Mod group compared to the Con group (p < 0.01, p < 0.05, p < 0.01), while HLB-H group treatment resulted in a decrease in the expression of these targets (p < 0.01, p < 0.01).

## 4 Discussion

DN, a major complication of diabetes, significantly impairs patients’ quality of life. As the prevalence of DN has risen in recent years (Wan et al., 2015), the identification of safe and effective therapeutic agents has become a critical focus of research. Notably, TCM has garnered attention for its potential in treating this condition. As a natural herbal medicine, HLB exhibits several pharmacological effects, including blood - glucose - lowering (Kim et al., 2023; van Dijk et al., 2009; Singh et al., 2010), blood - lipid - regulating (Gaba et al., 2011), anti - tumor (Gong et al., 2016; Allaoui et al., 2019; Alrumaihi et al., 2021), and organ - protecting (Almalki and Naguib, 2021), has been recognized for its safe and effective properties in diabetes management (Gupta et al., 2024; Hota et al., 2024; Kim et al., 2023; Pickering et al., 2022). In renal disease, HLB has been shown to reduce aluminum toxicity associated with renal failure in rats (Belaid-Nouira et al., 2013) and to exhibit therapeutic effects in DN treatment (Ghule et al., 2012; Sayed et al., 2012; Xue et al., 2011). Compared with commonly used drugs that have relatively single targets of action and significant adverse reactions (Sanchez-Rangel and Inzucchi, 2017; Dholariya et al., 2023), such as metformin and SGLT2 inhibitors, research has found that HLB can, through various components like trigonelline and polysaccharides, improve DN in multiple ways, including reducing blood sugar, regulating lipid metabolism, and countering oxidative stress, thus providing more treatment options for DN. Our experimental results have also confirmed this. Therefore, it is of great importance to conduct in - depth research on the potential mechanisms of HLB’s action on DN.

The high-fat diet combined with low-dose STZ injection is a widely adopted animal model for studying T2DM (Gonzalez et al., 2024; Singh et al., 2024). In this study, this model was induced using high-fat chow and STZ, and serum glucose levels, renal function markers, and histopathological alterations were assessed to confirm the presence of renal injury. Following a 4-week intervention with aqueous HLB extracts, the results indicated a significant reduction in blood glucose levels, improvement in renal function markers, and reversal of renal morphological damage. These findings demonstrate HLB’s capacity to substantially alleviate the symptoms of diabetes, including elevated glucose levels and insulin resistance. Furthermore, HLB was shown to significantly improve DN, which is characterized by elevated blood glucose, insulin resistance, increased renal volume, elevated glomerular filtration rate, and abnormal urinary albumin excretion in diabetic mice (KIDNEY DISEASE and IMPROVING GLOBAL OUTCOMES DIABETES WORK, 2022). HLB can counteract oxidative stress by improving indicators such as SOD, MDA, and GSH. These animal model results suggest that HLB exerts a therapeutic effect on DN.

High-performance liquid chromatography-mass spectrometry (HPLC-MS) is a standard technique for profiling the chemical constituents of traditional Chinese medicines (Li et al., 2022; Wei et al., 2021; Wu et al., 2022). In previous studies, it has been reported that HLB contains components such as polysaccharides (Prajapati et al., 2013), alkaloids (Liang et al., 2023), steroidal saponins (Wang et al., 2017a), and flavonoids (Banerjee et al., 2019; Singh et al., 2020). In this study, LC-MS was employed to identify the constituents of HLB aqueous extracts. A variety of compounds, including fenugreek alkaloids, protodiosgenin, isoprenoids, isoorientin, L-tryptophan, and adenosine, were detected in both positive and negative ion modes. A total of 34 major constituents, classified into flavonoids, carboxylic acids, steroids and derivatives, phenyls, purine nucleosides, isoprenoids, lipids, and indoles, were identified. Related studies (Bakhtiar et al., 2024; Singh et al., 2020) confirmed the presence of key components such as huperzine and isoprenoids, validating the reliability of the LC-MS methodology. These results provide foundational insights into the composition of HLB aqueous extracts, forming the basis for further investigations. However, due to the numerous components in HLB, there is currently limited research on the specific components or groups of components within HLB that exert effects on DN. In the future, in - depth studies on components such as polysaccharides, alkaloids, and flavonoids are still needed.

Network pharmacology has become a key approach for systematically unraveling the pharmacological mechanisms underlying the therapeutic effects of TCM in treating complex diseases (Chang et al., 2024; Han et al., 2022; Zhang et al., 2023). Several studies have integrated liquid-liquid coupling technology with network pharmacology to elucidate drug mechanisms of action (Darwish et al., 2022). In this study, 877 chemical constituent targets and 764 disease-related targets were identified through the combination of liquid-liquid coupling technology and network pharmacology, resulting in 159 intersecting targets. Among the 34 ingredients screened *via* the CTD network, key active compounds such as tretinoin, protofibrillar diosgenin, pseudomagnesoside II, robinoside BII, adenosine, guanosine, isoorientin, and orientin were identified as potential contributors to HLB’s effects on DN. Previous studies have highlighted the roles of tretinoin (Nie et al., 2020; Tang et al., 2023; Zhan et al., 2018), Zhimai saponin BII (Yuan et al., 2015), orientin (Kong et al., 2020), and isorhizobin (Kong et al., 2023) in DN treatment. The validation of these components’ roles through subsequent animal and cellular experiments remains a key focus for future research.

The integration of network pharmacology and metabolomics correlation analysis has been widely adopted in TCM research, offering a multi-faceted perspective on the material basis and mechanisms of action. This methodology has been applied in various contemporary studies to investigate the mechanisms by which drugs influence DN (Gao et al., 2024; Wang et al., 2021a). Among the top 50 signaling pathways identified through GO and KEGG enrichment analysis, the core targets strongly correlated with experimental findings primarily involved pathways related to lipid metabolism and atherosclerosis, the AGE-RAGE signaling pathway in diabetic complications, and the PI3K-Akt signaling pathway. Key targets implicated in these pathways included IL6, HSP90AA1, FN1, MAPK1, AKT1, PIK3R1, and MAPK3. Furthermore, metabolomics analysis identified 61 endogenous metabolites in mice kidney tissues, which were involved in pathways such as arginine and proline metabolism, galactose metabolism, amino sugar and nucleotide glucose metabolism, insulin resistance, and glutathione metabolism. Existing research has underscored the significance of galactose metabolism (Liu et al., 2021; Shen et al., 2023) and insulin resistance (Wang et al., 2021b; Wei et al., 2018) in DN treatment. This study, through the combined application of network pharmacology and untargeted metabolomics, revealed the AGE-RAGE and PI3K-Akt pathways as significantly altered in response to HLB treatment, leading to the identification of the PI3K-Akt-ERK signaling pathway as a potential mechanism underlying HLB’s therapeutic effects in DN.

Phosphatidylinositol-3-kinase (PI3K) is an intracellular lipid kinase with serine/threonine kinase activity (Xu et al., 2014) that plays a pivotal role in insulin signaling as a downstream target of insulin receptor substrates (IRS) (Feng et al., 2024). Protein kinase B (Akt), a serine/threonine kinase, is a primary effector in the PI3K signaling cascade (Huang et al., 2018b), mediating most of the metabolic effects of insulin. The PI3K/Akt signaling pathway is integral to insulin signal transduction (Li et al., 2017) and is closely implicated in the pathogenesis of T2DM (Lin et al., 2022). A significant body of research has highlighted the critical role of this pathway in the treatment of DN (Huang et al., 2018a; Li and Xu, 2022; Liu et al., 2024). The extracellular signal-regulated kinase (ERK) pathway, an essential component of the mitogen-activated protein kinase (MAPK) signaling network (Friedman and Perrimon, 2006), has also emerged as a key signaling axis in the investigation of DN mechanisms (Yu et al., 2023). In the present study, HLB treatment was shown to downregulate the expression of PI3K, Akt, and ERK proteins, implicating the PI3K-Akt-ERK pathway in the therapeutic action of HLB in DN. Numerous studies have explored the involvement of the PI3K/Akt, ERK, and PPARγ signaling pathways in DN therapy. Wang Z et al. (Wang et al., 2023) established a diabetic kidney disease (DKD) rat model and administered the Chinese herbal compound Qiqi Burdock and Kidney Benefit Soup (QBF). Their findings revealed that QBF treatment significantly downregulated the expression of PI3K, AKT, and ERK1/2 proteins in the kidney tissues of DKD rats, thus confirming that HLB administration similarly reduces the expression of these proteins. These results underscore the potential role of the PI3K-Akt-ERK signaling pathway in the therapeutic effects of DN treatment. QRT-PCR was performed to assess the mRNA levels of MAPK1, MAPK3, AKT1, PI3K, HSP90AA1, and FN1 following drug intervention, showing that HLB administration modulated the expression of these genes, which in turn delayed renal function deterioration. WB analysis further corroborated the impact of HLB on the expression patterns of renal PI3K, AKT and ERK proteins, demonstrating that HLB regulates key proteins in these signaling pathways. Collectively, these experiments validate that HLB may improve DN by modulating the PI3K-Akt-ERK signaling pathway.

This experiment was an early attempt to utilize techniques such as UHPLC - HRMS, network pharmacology, and metabolomics to investigate the mechanism of action of water - extracted HLB in the treatment of DN. The study found that the PI3K - Akt - ERK signaling pathway plays a crucial role in HLB’s improvement of DN. This pathway may alleviate renal function damage under hyperglycemic conditions by improving blood glucose levels and abnormal lipid metabolism. These research results support the potential of HLB as a natural medicine for the treatment or prevention of DN, providing a valuable basis for further research. However, it should be noted that further cell experiments and clinical studies are needed to verify our conclusions.

## 5 Conclusion

In conclusion, this study provides preliminary insights into the therapeutic effects and underlying mechanisms of HLB on DN, utilizing a multifaceted approach combining animal models, network pharmacology, untargeted metabolomics, and association analysis.

The findings suggest that HLB may exert its therapeutic effects through the coordinated action of multiple active ingredients targeting MAPK1, MAPK3, AKT1, and PI3K. This interaction influences the PI3K-Akt-ERK signaling pathway and lipid metabolism pathways, while also improving oxidative stress levels. These results offer a novel perspective for exploring the therapeutic targets and mechanisms of HLB in the treatment of DN.

## Data Availability

The original contributions presented in the study are included in the article/supplementary material, further inquiries can be directed to the corresponding authors.
