# Supplementary material for: Trigonella foenum-graecum L. protects against renal function decline in a mouse model of type 2 diabetic nephropathy by modulating the PI3K-Akt-ERK signaling pathway
Source: Front Pharmacol. 2025 Mar 18;16:1566723. doi: 10.3389/fphar.2025.1566723 (PMC11959092; doi:10.3389/fphar.2025.1566723)
Supplement: Supplementary file 1 [file DataSheet2.pdf]

Supplementary Table

Table 1. Information on 159 common targets of components and diseases

NO.	Gene	NO.	Gene	NO.	Gene	NO.	Gene
1	PTGS1	41	ABCC8	81	HSPA5	121	STAT1
2	PTGS2	42	ACVR1	82	MAPK1	122	REG1A
3	NFE2L2	43	ADORA1	83	HSPA1A	123	SORD
4	NQO1	44	AKT1	84	HPRT1	124	TGFB2
5	CASP3	45	CFTR	85	CD38	125	GSR
6	AR	46	CREB1	86	HK2	126	BMP7
7	RELA	47	ENPP1	87	TH	127	GCK
8	IKBKB	48	GAPDH	88	KDM4C	128	SOD2
9	TNF	49	GSK3B	89	SLC15A1	129	NOS2
10	NR3C1	50	HK1	90	SLC22A6	130	PLAU
11	VEGFA	51	IGF1R	91	HLA-A	131	APRT
12	CCND1	52	MAPK3	92	ACE2	132	PCK1
13	FLT1	53	PIK3CA	93	REN	133	JAK2
14	KDR	54	PRKAB1	94	HMGCR	134	IGF1
15	MMP9	55	PRKAR1A	95	EDNRA	135	PGF
16	AKR1B1	56	PRMT1	96	PRKCD	136	MET
17	MMP2	57	TLR7	97	KLK1	137	GC

18	TIMP1	58	HRAS	98	HTR1A	138	LCN2
19	TIMP2	59	RAC1	99	MPO	139	CYP2C8
20	MMP1	60	TGM2	100	PTPN2	140	CYP2C9
21	FN1	61	HBA1	101	CCR5	141	TGFBR2
22	TGFB1	62	HBB	102	BDKRB1	142	CAT
23	COL4A4	63	FGF2	103	CTSB	143	MAPK14
24	ADRB2	64	HSP90AA1	104	NOX4	144	HMOX1
25	DPP4	65	HTR2A	105	SLC5A1	145	TTR
26	LTA4H	66	STAT3	106	XDH	146	APOA2
27	SRC	67	IL2	107	SELP	147	ALB
28	BCHE	68	PTPN1	108	PPARA	148	PIK3R1
29	NOS1	69	AGTR2	109	NLRP3	149	PARP1
30	AGXT	70	ACE	110	PPARG	150	G6PD
31	IL1B	71	MME	111	NR1H2	151	AKT2
32	IL6	72	F2	112	ESR1	152	CFB
33	SLCO1B3	73	TLR9	113	ESR2	153	PLG
34	LPL	74	PTPN11	114	RBP4	154	INSR
35	VDR	75	CYP19A1	115	GCG	155	LPA
36	HSD11B2	76	ACP1	116	FABP1	156	HGF
37	ABCA1	77	SHBG	117	NOS3	157	CES1

38	ABCB1	78	NR3C2	118	CCL5	158	PIK3CG
39	ABCB11	79	JUN	119	SELE	159	MAPK8
40	ABCC2	80	CASP1	120	TAP1		

Table 2. Information on 10 GO-BP pathways

NO.	Category	ID	Term	PValue	Genes	FDR
1	GOTERM_BP_DIRECT	GO:0001666	response to hypoxia	1.06E-17	TGFB2, TGFB1, ACE, NOS2, MMP2, SOD2, TNF, PGF, HK2, VEGFA, DPP4, HSD11B2, EDNRA, TH, PLAU, CASP3, CAT, ADORA1, CD38, NOS1, PPARA	2.50E-14
2	GOTERM_BP_DIRECT	GO:0051897	positive regulation of phosphatidylinositol 3-kinase/protein kinase B signal	5.31E-17	TGFB2, TGFB1, FLT1, SRC, HGF, INSR, FN1,	6.26E-14

			transduction		IGF1, F2, FGF2, TNF, PIK3CG, IGF1R, VEGFA, SELP, PIK3CA, CCL5, CAT, KDR, NOX4, JAK2	
3	GOTERM_BP_DIRECT	GO:0043066	negative regulation of apoptotic process	3.83E-16	GSK3B, SRC, AKR1B1, PIK3R1, MPO, TNF, RELA, IGF1R, MAPK8, ADORA1, KDR, AKT1, CD38, TIMP1, TGM2, NQO1, HSPA5, HGF, GCG, IGF1, SOD2, MMP9, IL2, VEGFA, FABP1, IL6, CREB1, CAT, HSPA1A	3.01E-13

4	GOTERM_BP_DIRECT	GO:0032496	response to lipopolysaccharide	8.63E-16	NQO1, ACE, NOS2, NOS3, ABCC8, PTGS2, SOD2, SELE, MPO, SELP, TH, IL1B, CASP3, BDKRB1, REN, NOS1, JAK2, PCK1	4.96E-13
5	GOTERM_BP_DIRECT	GO:0009410	response to xenobiotic stimulus	1.05E-15	HSP90AA1, TGFB1, ACE, ABCB1, ABCC8, STAT1, SRC, MMP2, APOA2, HTR2A, PTGS2, SOD2, TNF, RELA, TGFBR2, HSD11B2, CREB1, CCND1, CASP3, CAT, CD38, PPARG	4.96E-13
6	GOTERM_BP_DIRECT	GO:0045944	positive regulation of transcription by	2.95E-15	ADRB2, PIK3R1, NR3C1,	1.16E-12

			RNA polymerase II		FGF2, TNF, RELA, IKBKB, AKT1, NLRP3, NOS1, JAK2, PCK1, HRAS, MAPK3, ACVR1, JUN, TGFB1, KDM4C, PARP1, HSPA5, STAT1, VDR, HGF, NR1H2, STAT3, IGF1, MAPK14, BMP7, ESR1, IL2, ESR2, VEGFA, AR, IL6, CREB1, IL1B, TLR9, TLR7, PPARG, PPARA, MET, NFE2L2	
7	GOTERM_BP_DIRECT	GO:0048661	positive regulation of smooth muscle cell proliferation	9.98E-15	TGFB1, STAT1, PIK3R1, IGF1, PTGS2, FGF2,	3.36E-12

					TNF, IGF1R, IL6, PIK3CA, CCL5, AKT1, HMOX1	
8	GOTERM_BP_DIRECT	GO:0008284	positive regulation of cell population proliferation	2.96E-14	FLT1, REG1A, HTR2A, FGF2, RELA, IGF1R, DPP4, KDR, TIMP1, JAK2, HRAS, TGFB2, TGFB1, KDM4C, PRMT1, INSR, HTR1A, FN1, IGF1, F2, IL2, PGF, TGFBR2, VEGFA, AR, IL6, IL1B	8.73E-12
9	GOTERM_BP_DIRECT	GO:0006954	inflammatory response	3.53E-14	TNF, RELA, HK2, PIK3CG, HK1, IKBKB, CCL5, ADORA1, AKT1, BDKRB1, NLRP3,	9.26E-12

					RAC1, CCR5, TGFB1, NOS2, STAT3, SELE, SELP, IL6, IL1B, TLR9, NOX4, TLR7, AGTR2, NFE2L2	
10	GOTERM_BP_DIRECT	GO:0030335	positive regulation of cell migration	1.39E-12	ACVR1, TGFB1, FLT1, HSPA5, HGF, MMP2, INSR, STAT3, IGF1, SOD2, IGF1R, VEGFA, PLAU, IL1B, CCL5, AKT2, KDR, AKT1, JAK2, HRAS	3.28E-10

Table 3. Information on 10 GO-CC pathways

NO.	Category	ID	Term	PValue	Genes	FDR
1	GOTERM_CC_DI	GO:00055	extracellular	9.77E-1	HBB, MPO,	2.48E-1

	RECT	76	region	9	FGF2, TNF, PLAU, TIMP2, KDR, ENPP1, TIMP1, TGM2, CTSB, HSP90AA 1, MMP1, HGF, MMP2, PRKCD, APOA2, HBA1, F2, MMP9, PGF, TGFBR2, APRT, ACE2, RBP4, COL4A4, IL1B, CAT, TLR9, LPA, MET, CFB, LPL, PLG, DPP4, TTR, CCL5, MAPK1, NLRP3, LTA4H, GC, BCHE, TGFB2, TGFB1, ACE, FN1, GCG, IGF1,	6

					MAPK14, BMP7, IL2, VEGFA, IL6, ALB, LCN2, REN, SHBG, HSPA1A	
2	GOTERM_CC_DIRECT	GO:0005615	extracellular space	8.86E-16	FLT1, REG1A, HBB, AKR1B1, MPO, FGF2, TNF, PLAU, TIMP2, ENPP1, TIMP1, CTSB, MMP1, HGF, MMP2, SORD, HLA-A, HBA1, F2, MMP9, PGF, TGFBR2, ACE2, RBP4, COL4A4, IL1B, CAT, LPA, CFB, LPL, PLG, TTR, CCL5, HMOX1, GC, XDH, BCHE, TGFB2,	1.12E-13

					TGFB1, ACE, FN1, GCG, IGF1, SELE, BMP7, IL2, VEGFA, SELP, IL6, ALB, LCN2, REN, HSPA1A	
3	GOTERM_CC_DIRECT	GO:0070062	extracellular exosome	2.50E-13	REG1A, HBB, AKR1B1, MPO, PLAU, CD38, TIMP1, RAC1, ACP1, TGM2, CTSB, G6PD, HSP90AA1, MME, PRKCD, APOA2, SORD, HLA-A, HBA1, F2, MMP9, APRT, ACE2, RBP4, CAT, HPRT1, GAPDH, CFB, SLC22A6, ABCB1,	2.12E-11

					KLK1, SRC, PLG, SLC5A1, ABCB11, PTGS1, DPP4, TTR, LTA4H, GC, PCK1, ACE, HSPA5, INSR, GSR, FN1, SOD2, FABP1, ALB, LCN2, SHBG, HSPA1A	
4	GOTERM_CC_DIRECT	GO:0005901	caveola	1.05E-11	SLC22A6, SRC, NOS3, INSR, MAPK1, HTR2A, JAK2, PTGS2, SELE, TGFBR2, IGF1R, MAPK3	6.70E-10
5	GOTERM_CC_DIRECT	GO:0005886	plasma membrane	4.63E-11	GSK3B, FLT1, HTR2A, TNF, PIK3CG, IGF1R, EDNRA, PLAU, AKT2, ADORA1,	2.35E-09

CASP1,
KDR,
ENPP1,
CD38,
AKT1,
BDKRB1,
NOS1,
RAC1,
JAK2,
CCR5,
HRAS,
TGM2,
ACVR1,
SLC15A1,
HSP90AA
1, ABCC2,
MME,
ABCC8,
MMP2,
PRKCD,
TAP1,
HLA-A,
F2,
TGFBR2,
ACE2,
AR,
PIK3CA,
PRKAR1
A, TLR9,
TLR7,
AGTR2,
MET,
GAPDH,
CFTR,
CFB,
SLC22A6,
ABCB1,
SRC, LPL,
PLG,
ADRB2,
PIK3R1,
SLC5A1,
ABCB11,
DPP4,

					MAPK8, SLCO1B3, MAPK1, MAPK3, ABCA1, PTPN1, BCHE, TGFB1, ACE, NOS2, HSPA5, NOS3, INSR, STAT3, HTR1A, FN1, GCG, SELE, ESR1, SELP, CYP2C9, CYP2C8, NOX4, REN, PTPN2, NFE2L2, HSPA1A	
6	GOTERM_CC_DIRECT	GO:0031093	platelet alpha granule lumen	4.51E-08	TGFB2, TGFB1, HGF, ALB, FN1, PLG, TIMP1, IGF1, VEGFA	1.91E-06
7	GOTERM_CC_DIRECT	GO:0045121	membrane raft	7.49E-08	ABCA1, MME, SRC, SELE, TNF, TGFBR2, HK1,	2.72E-06

					DPP4, IKBKB, ACE2, KDR, NOS1, JAK2	
8	GOTERM_CC_DIRECT	GO:0005788	endoplasmic reticulum lumen	1.10E-07	BCHE, HSPA5, APOA2, FN1, GCG, F2, PTGS2, ACE2, IL6, COL4A4, ALB, MAPK1, TIMP1, CES1, MAPK3	3.49E-06
9	GOTERM_CC_DIRECT	GO:0009897	external side of plasma membrane	1.74E-07	ABCA1, ACE, INSR, GSR, PLG, HLA-A, F2, SELE, TNF, TGFBR2, SELP, PLAU, KDR, BDKRB1, AGTR2, CCR5, CTSB	4.91E-06
10	GOTERM_CC_DIRECT	GO:0005829	cytosol	4.24E-07	GSK3B, HBB, AKR1B1, NR3C1, HK2, PIK3CG,	1.08E-05

HK1,
NR3C2,
IKBKB,
CCND1,
CASP3,
AKT2,
CASP1,
AKT1,
NOS1,
RAC1,
JAK2,
ACP1,
HRAS,
TGM2,
G6PD,
HSP90AA
1, PARP1,
PRMT1,
PRKCD,
APOA2,
SORD,
HBA1,
PRKAB1,
TGFBR2,
APRT,
AR,
PIK3CA,
PRKAR1
A, IL1B,
CAT,
PPARG,
HPRT1,
AGXT,
GAPDH,
CFTR,
CES1,
SRC,
PIK3R1,
RELA,
MAPK8,
HMOX1,
MAPK1,
NLRP3,
LTA4H,

GC, PCK1, XDH, MAPK3, PTPN1, NQO1, NOS2, HSPA5, NOS3, STAT1, VDR, NR1H2, STAT3, GSR, PTPN11, MAPK14, ESR1, GCK, FABP1, TH, PTPN2, NFE2L2, HSPA1A

Table 4. Information on 10 GO-MF pathways

NO.	Category	ID	Term	PValue	Genes	FDR
1	GOTERM_MF_DIRECT	GO:0019899	enzyme binding	1.97E-21	SRC, PLG, PTGS2, RELA, MAPK8, CCND1, AKT1, HMOX1, RAC1, PTPN1, BCHE, JUN, TGFB1, KDM4C	1.21722605130708E-18

					, PARP1, HSPA5, PRMT1, STAT1, PRKCD, APOA2, MAPK14, SOD2, ESR1, ESR2, AR, CREB1, TH, CAT, PPARG, CFTR, HSPA1A	
2	GOTERM_MF_DIRECT	GO:0042802	identical protein binding	2.42E-17	HTR2A, NR3C1, FGF2, TNF, PIK3CG, IGF1R, IKBKB, CASP1, KDR, CD38, AKT1, JAK2, CCR5, G6PD, HSP90AA1, PARP1, PRMT1, HGF, SORD, MMP9, ACE2, CREB1, CAT,	7.48695008956875E-15

					PPARG, HPRT1, AGXT, MET, GAPDH, SLC22A6, ADRB2, RELA, DPP4, TTR, CCL5, HMOX1, MAPK1, NLRP3, MAPK3, NQO1, BCHE, JUN, TGFB1, STAT1, INSR, STAT3, FN1, GCG, SOD2, ESR1, VEGFA, TH, ALB, LCN2	
3	GOTERM_MF_DIRECT	GO:0020037	heme binding	2.57E-12	NOS2, NOS3, SRC, HBB, HBA1, PTGS2, MPO, CYP19A1, PTGS1,	0.0000000005290711697

					CYP2C9, CYP2C8, CAT, NOX4, HMOX1, NOS1, JAK2	
4	GOTERM_MF_DIRECT	GO:0004879	nuclear receptor activity	1.78E-11	AR, VDR, NR1H2, STAT3, PPARG, PIK3R1, PPARA, NR3C1, ESR1, ESR2, NR3C2	2.74827238712355E-09
5	GOTERM_MF_DIRECT	GO:0005515	protein binding	7.87E-10	NR3C1, MPO, FGF2, TNF, IGF1R, NR3C2, EDNRA, CCND1, PLAU, AKT2, ADORA1, KDR, ENPP1, AKT1, TGM2, ACVR1, G6PD, HGF, PRKCD, TAP1, HLA-A, PRKAB1	9.72188295646529E-08

, PGF,
AR,
ACE2,
RBP4,
PRKAR
1A,
HPRT1,
AGTR2,
LPA,
CFTR,
CFB,
SLC22A
6,
ABCB1,
LPL,
SLC5A1,
ADRB2,
PIK3R1,
DPP4,
HMOX1
,
NLRP3,
LTA4H,
ABCA1,
TGFB2,
JUN,
TGFB1,
HSPA5,
VDR,
INSR,
HTR1A,
FN1,
IGF1,
SELE,
ESR1,
BMP7,
ESR2,
IL2,
SELP,
CYP2C8
, IL6,
TH,
ALB,
LCN2,

NOX4,
REN,
SHBG,
NFE2L2,
GSK3B,
FLT1,
HBB,
AKR1B1
,
HTR2A,
HK2,
PIK3CG,
HK1,
IKBKB,
CASP3,
CASP1,
TIMP2,
BDKRB1
, TIMP1,
NOS1,
RAC1,
JAK2,
CCR5,
ACP1,
HRAS,
CTSB,
HSP90A
A1,
ABCC2,
PARP1,
MME,
PRMT1,
MMP2,
APOA2,
HBA1,
F2,
MMP9,
APRT,
TGFBR2
, CREB1,
PIK3CA
, IL1B,
TLR7,
PPARG,

					PPARA, AGXT, GAPDH, MET, SRC, PLG, HMGCR, PTGS2, ABCB11, RELA, PTGS1, MAPK8, TTR, CCL5, MAPK1, XDH, MAPK3, BCHE, PTPN1, NQO1, NOS2, STAT1, NOS3, NR1H2, STAT3, GCG, PTPN11, MAPK14, SOD2, GCK, VEGFA, FABP1, PTPN2, HSPA1A	
6	GOTERM_MF_DIRECT	GO:0042803	protein homodimerization activity	2.22E-09	LPL, ADRB2, PTGS2, RELA, DPP4, IKBKB,	2.28462185256925E-07

					CCL5, AKT1, HMOX1, ENPP1, XDH, ACVR1, TGFB2, G6PD, HSP90AA1, PARP1, MME, NOS2, STAT1, STAT3, APOA2, TAP1, VEGFA, CAT, TLR9, AGXT	
7	GOTERM_MF_DIRECT	GO:0005102	signaling receptor binding	9.97E-09	ABCA1, TGFB2, SRC, HGF, STAT3, APOA2, FN1, LPL, GCG, PLG, HLA-A, F2, DPP4, AR, REN, JAK2, LPA, HSPA1A	8.80041347100815E-07

8	GOTERM_MF_DIRECT	GO:0008083	growth factor activity	1.14E-08	TGFB2, TGFB1, HGF, REG1A, IGF1, F2, FGF2, BMP7, IL2, PGF, VEGFA, IL6, TIMP1	8.81482743692162E-07
9	GOTERM_MF_DIRECT	GO:0005158	insulin receptor binding	1.83E-08	PTPN1, SRC, ENPP1, PTPN11, IGF1, PIK3R1, IGF1R	1.25661478205808E-06
10	GOTERM_MF_DIRECT	GO:0005524	ATP binding	2.37E-08	GSK3B, FLT1, ABCB1, SRC, ABCB11, HK2, PIK3CG, IGF1R, HK1, IKBKB, MAPK8, AKT2, KDR, AKT1, MAPK1, ENPP1, NLRP3, JAK2, TGM2, MAPK3, ABCA1, ACVR1,	1.46506156146916E-06

					HSP90AA1, ABCC2, HSPA5, ABCC8, INSR, PRKCD, TAP1, MAPK14, GCK, TGFBR2, PIK3CA, MET, CFTR, HSPA1A	

Table 5. Information on 30 KEGG pathways

NO.	Category	ID	Term	PValue	Genes	FDR
1	KEGG_PATHWAY	hsa04933	AGE-RAGE signaling pathway in diabetic complications	3.51E-14	IL6, JUN, STAT3, FN1, MAPK1, AKT1, PIK3R1, TNF, MAPK3	2.18E-12
2	KEGG_PATHWAY	hsa05417	Lipid and atherosclerosis	1.39E-13	IL6, HSP90AA1, JUN, SRC, STAT3, MAPK1, AKT1, PIK3R1, TNF,	4.30E-12

					MAPK3	
3	KEGG_PATHWAY	hsa05135	Yersinia infection	4.56E-13	IL6, JUN, SRC, FN1, MAPK1, AKT1, PIK3R1, TNF, MAPK3	9.43E-12
4	KEGG_PATHWAY	hsa05161	Hepatitis B	1.77E-12	IL6, JUN, SRC, STAT3, MAPK1, AKT1, PIK3R1, TNF, MAPK3	2.75E-11
5	KEGG_PATHWAY	hsa04625	C-type lectin receptor signaling pathway	8.65E-12	IL6, JUN, SRC, MAPK1, AKT1, PIK3R1, TNF, MAPK3	1.07E-10
6	KEGG_PATHWAY	hsa05205	Proteoglycans in cancer	1.09E-11	SRC, STAT3, FN1, MAPK1, AKT1, PIK3R1, ESR1, TNF, MAPK3	1.13E-10
7	KEGG_PATHWAY	hsa05207	Chemical carcinogenesis - receptor activation	1.67E-11	HSP90AA1, JUN, SRC, STAT3, MAPK1, AKT1, PIK3R1, ESR1,	1.48E-10

					MAPK3	
8	KEGG_PATHW AY	hsa0491 5	Estrogen signaling pathway	6.40E-1 1	HSP90AA1 , JUN, SRC, MAPK1, AKT1, PIK3R1, ESR1, MAPK3	4.96E-1 0
9	KEGG_PATHW AY	hsa0491 7	Prolactin signaling pathway	9.69E-1 1	SRC, STAT3, MAPK1, AKT1, PIK3R1, ESR1, MAPK3	6.67E-1 0
10	KEGG_PATHW AY	hsa0152 1	EGFR tyrosine kinase inhibitor resistance	2.02E-1 0	IL6, SRC, STAT3, MAPK1, AKT1, PIK3R1, MAPK3	1.25E-0 9
11	KEGG_PATHW AY	hsa0520 0	Pathways in cancer	4.87E-1 0	IL6, HSP90AA1 , JUN, STAT3, FN1, MAPK1, AKT1, PIK3R1, ESR1, MAPK3	2.74E-0 9
12	KEGG_PATHW AY	hsa0516 7	Kaposi sarcoma-associate d herpesvirus infection	7.25E-1 0	IL6, JUN, SRC, STAT3, MAPK1, AKT1, PIK3R1, MAPK3	3.57E-0 9

13	KEGG_PATHWAY	hsa01522	Endocrine resistance	7.48E-10	JUN, SRC, MAPK1, AKT1, PIK3R1, ESR1, MAPK3	3.57E-09
14	KEGG_PATHWAY	hsa05142	Chagas disease	9.52E-10	IL6, JUN, MAPK1, AKT1, PIK3R1, TNF, MAPK3	4.22E-09
15	KEGG_PATHWAY	hsa04620	Toll-like receptor signaling pathway	1.34E-09	IL6, JUN, MAPK1, AKT1, PIK3R1, TNF, MAPK3	5.56E-09
16	KEGG_PATHWAY	hsa05163	Human cytomegalovirus infection	1.97E-09	IL6, SRC, STAT3, MAPK1, AKT1, PIK3R1, TNF, MAPK3	7.63E-09
17	KEGG_PATHWAY	hsa04668	TNF signaling pathway	2.29E-09	IL6, JUN, MAPK1, AKT1, PIK3R1, TNF, MAPK3	8.36E-09
18	KEGG_PATHWAY	hsa04012	ErbB signaling pathway	3.42E-08	JUN, SRC, MAPK1, AKT1, PIK3R1, MAPK3	1.18E-07

19	KEGG_PATHWAY	hsa05235	PD-L1 expression and PD-1 checkpoint pathway in cancer	4.31E-08	JUN, STAT3, MAPK1, AKT1, PIK3R1, MAPK3	1.40E-07
20	KEGG_PATHWAY	hsa04657	IL-17 signaling pathway	5.66E-08	IL6, HSP90AA1, JUN, MAPK1, TNF, MAPK3	1.69E-07
21	KEGG_PATHWAY	hsa04510	Focal adhesion	5.72E-08	JUN, SRC, FN1, MAPK1, AKT1, PIK3R1, MAPK3	1.69E-07
22	KEGG_PATHWAY	hsa05166	Human T-cell leukemia virus 1 infection	1.00E-07	IL6, JUN, MAPK1, AKT1, PIK3R1, TNF, MAPK3	2.82E-07
23	KEGG_PATHWAY	hsa04659	Th17 cell differentiation	1.08E-07	IL6, HSP90AA1, JUN, STAT3, MAPK1, MAPK3	2.91E-07
24	KEGG_PATHWAY	hsa04066	HIF-1 signaling pathway	1.13E-07	IL6, STAT3, MAPK1, AKT1, PIK3R1, MAPK3	2.92E-07

25	KEGG_PATHWAY	hsa05171	Coronavirus disease - COVID-19	1.48E-07	IL6, JUN, STAT3, MAPK1, PIK3R1, TNF, MAPK3	3.66E-07
26	KEGG_PATHWAY	hsa05131	Shigellosis	1.93E-07	JUN, SRC, MAPK1, AKT1, PIK3R1, TNF, MAPK3	4.33E-07
27	KEGG_PATHWAY	hsa04660	T cell receptor signaling pathway	1.99E-07	JUN, MAPK1, AKT1, PIK3R1, TNF, MAPK3	4.33E-07
28	KEGG_PATHWAY	hsa04919	Thyroid hormone signaling pathway	1.99E-07	SRC, MAPK1, AKT1, PIK3R1, ESR1, MAPK3	4.33E-07
29	KEGG_PATHWAY	hsa05132	Salmonella infection	2.02E-07	IL6, HSP90AA1, JUN, MAPK1, AKT1, TNF, MAPK3	4.33E-07
30	KEGG_PATHWAY	hsa04151	PI3K-Akt signaling pathway	1.76E-06	IL6, HSP90AA1, FN1, MAPK1, AKT1, PIK3R1,	2.33E-06

MAPK3
