# Supplementary material for: Trigonella foenum-graecum L. protects against renal function decline in a mouse model of type 2 diabetic nephropathy by modulating the PI3K-Akt-ERK signaling pathway
Source: Front Pharmacol. 2025 Mar 18;16:1566723. doi: 10.3389/fphar.2025.1566723 (PMC11959092; doi:10.3389/fphar.2025.1566723)
Supplement: Supplementary file 2 [file DataSheet1.pdf]

Supplementary Material

Supplementary Table S1

Table S1. Mass spectrometry parameter settings

No.	Description	parameters
1	Scan type（m/z）	70-1050
2	Sheath gas flow rate （arb）	50
3	Aux gas flow rate （arb）	13
4	Heater temp（℃）	425
5	Capillary temp（℃）	325
6	Spray voltage（+）（V）	3500
7	Spray voltage（-）（V）	-3500
8	S-Lens RF Level	50
9	Normalized collision energy（eV）	20，40，60
10	Resolution（Full MS）	60000
11	Resolution（MS^2）	7500

Supplementary Table S2

Table S2. Sequence of QRT-PCR primers for kidney tissue-related indicators

No.	Primer name	primer sequence

1	*Fn1*	upstream primer ATGTGGACCCCTCCTGATAGT (forward)
2		downstream primer GCCCAGTGATTTCAGCAAAGG (reverse)
3	*Akt1*	upstream primer ATGAACGACGTAGCCATTGTG (forward)
4		downstream primer TTGTAGCCAATAAAGGTGCCAT (reverse)
5	*Mapk3*	upstream primer TCCGCCATGAGAATGTTATAGGC (forward)
6		downstream primer GGTGGTGTTGATAAGCAGATTGG (reverse)
7	*Mapk1*	upstream primer GGTTGTTCCCAAATGCTGACT (forward)
8		downstream primer CAACTTCAATCCTCTTGTGAGGG (reverse)
9	*Hsp90aa1*	upstream primer AATTGCCCAGTTAATGTCCTTGA (forward)
10		downstream primer CGTCCGATGAATTGGAGATGAG (reverse)
11	*Pik3r1*	upstream primer ACACCACGGTTTGGACTATGG (forward)<br>downstream primer GGCTACAGTAGTGGGCTTGG (reverse)

Supplementary Table S3

Table S3. Identification information of 34 compounds in HLB

No.	m/z	RT/ min	ppm	compound name	adduct	score	Class	PubCHEM CID
1	138.0546	0.94	2.1	Trigonelline	[M+H]+	0.9998	unknown	Cid_5570
2	268.1044	2.82	1.4	Adenosine	[M+H]+	0.9997	Purine_nucleosides	Cid_447270
3	284.0986	2.93	1.1	Guanosine	[M+H]+	0.9997	Purine_nucleosides	Cid_135398635
4	166.0859	3.48	17	Phenylalanine	[M+H]+	0.9997	Carboxylic_acids_and_derivatives	Cid_6140
5	311.1234	3.61	1.4	gamma-Glutamyltyrosine	[M+H]+	0.9477	Carboxylic_acids_and_derivatives	Cid_94340
6	252.0725	3.68	1.1	Adenylsuccinic acid	[M+H-C5H9O7P]+	0.9488	Purine_nucleotides	Cid_447145
7	205.0969	4.13	1.2	L-Tryptophan	[M+H]+	0.9992	Indoles_and_derivatives	Cid_6305
8	295.1287	4.45	0.9	gamma-Glutamylphenylalanine	[M+H]+	0.9779	Carboxylic_acids_and_derivatives	Cid_111299
9	595.1653	4.63	0.1	Vicenin-2	[M+H]+	0.9603	Flavonoids	Cid_442664

10	565.1545	4.84	1.9	Neoschaftoside	[M+H]+	0.9267	Flavonoids	Cid_3550102
11	565.1545	4.98	1.5	Schaftoside	[M+H]+	0.8074	Flavonoids	Cid_182689
12	449.1076	5.03	0.3	Isoorientin	[M+H]+	0.9982	Flavonoids	Cid_114776
13	449.1074	5.18	1	Orientin	[M+H]+	0.9875	Flavonoids	Cid_5281675
14	433.1127	5.50	1.2	Isovitexin	[M+H]+	0.9783	Flavonoids	Cid_162350
15	889.4778	6.45	1.8	NCGC00385394-01	[M-H2O+H]+	0.8521	Steroids_and_steroid_derivatives	Cid_133053868
16	903.4928	6.61	2.5	Trigoneoside Xb	[M-H2O+H]+	0.857	Steroids_and_steroid_derivatives	Cid_75528901
17	595.1444	7.06	0.6	Tiliroside	[M+H]+	0.809	Flavonoids	Cid_5320686
18	1031.5419	7.43	0.3	Pseudoprotodioscin	[M+H]+	0.9747	Steroids_and_steroid_derivatives	Cid_122130315
19	885.4831	7.66	1.4	Gracillin	[M+H]+	0.9079	Steroids_and_steroid_derivatives	Cid_159861
20	520.3393	13.43	0.6	1-Linoleoyl-sn-glycero-3-phosphorylcholine	[M+H]+	0.9518	Glycerophospholipids	Cid_11988421
21	117.0180	2.15	12.4	Succinic acid	[M-H]-	0.9994	Carboxylic_acids_and_derivatives	Cid_1110
22	134.0460	2.90	9.5	Decoyinine	[M-H-	0.9995	Imidazopyrimidines	Cid_121578

					C6H8O4]-			
3	282.0838	3.06	2.6	Guanosine	[M-H]-	0.9821	Purine_nucleosides	Cid_135398635
5	309.1087	3.66	2.2	gamma-Glutamyltyrosine	[M-H]-	0.9842	Carboxylic_acids_and_derivatives	Cid_94340
23	180.0291	4.15	5.9	5-Aminoisophthalic acid	[M-H]-	0.9592	Benzene_and_substituted_derivatives	Cid_66833
8	293.1139	4.50	2.3	gamma-Glutamylphenylalanine	[M-H]-	0.9786	Carboxylic_acids_and_derivatives	Cid_111299
9	593.1500	4.66	2	Vicenin-2	[M-H]-	0.9684	Flavonoids	Cid_442664
24	563.1394	4.87	2.5	Isoschaftoside	[M-H]-	0.9225	Flavonoids	Cid_3084995
11	563.1393	5.02	2	Schaftoside	[M-H]-	0.9534	Flavonoids	Cid_182689
12	447.0929	5.07	1.8	Isoorientin	[M-H]-	0.9867	Flavonoids	Cid_114776
13	447.0922	5.22	0	Orientin	[M-H]-	0.9987	Flavonoids	Cid_5281675
14	431.0974	5.54	3	Isovitexin	[M-H]-	0.9719	Flavonoids	Cid_162350
25	547.1447	5.93	1.7	5,7-dihydroxy-2-phenyl-6-[3,4,5-trihydroxy-6-(hydroxymethyl)oxan-2-yl]-8-(3,4,5-trihydroxyoxan-2-yl)-	[M-H]-	0.7797	Flavonoids	Cid_44715843

				4H-chromen-4-one				
26	963.4782	6.65	15.6	Bacopaside II	[M+Cl]-	0.7704	Prenol_lipids	Cid_9876264
27	557.1289	6.76	21.7	Cidofovir	[2M-H]-	0.812	Diazines	Cid_60613
28	919.4894	7.14	0.5	Timosaponin b ii	[M-H]-	0.9267	Steroids_and_steroid_derivatives	Cid_53486384
29	1063.5308	7.38	0.9	Protogracillin	[M-H]-	0.7681	Steroids_and_steroid_derivatives	Cid_441892
30	1047.5355	7.49	1.4	Protodioscin	[M-H]-	0.8584	Steroids_and_steroid_derivatives	Cid_441891
31	947.4831	7.71	3.4	(3S)-5-((1S,2R,4S,4aR,8aR)-4-((4-O-(6-O-Acetyl-.beta.-D-glucopyranosyl)-6-deoxy-.alpha.-L-mannopyranosyl)oxy)-1,2,4a,5-tetramethyl-1,2,3,4,4a,7,8,8a-octahydronaphthalen-1-yl)-3-methylpent-1-en-3-yl 6-deoxy-4-O-(6-deoxy-.alpha.-L-mannopyranosyl)-.beta.-D-galactopyranoside	[M-H]-	0.8182	Prenol_lipids	Cid_10724564

32	449.2747	8.43	11	Celastrol	[M-H]-	0.9188	Prenol_lipids	Cid_122724
33	941.5089	11.08	2.9	MCULE-7407861812	[M-H]-	0.9694	Prenol_lipids	Cid_44715452
34	295.2272	15.44	2.8	12(13)-EpOME	[M-H]-	0.9892	Fatty_Acyls	Cid_5356421

Supplementary Table S4

Table S4. 354 differential metabolites detected by Mod_vs_Con.

No.	Metabolite	RT/min	KEGG Compound ID	Mode	M/Z	RSD	VIP	P_value
1	Hydroxyprolyl-Valine	1.4374	-	pos	213.1237882	0.002452136	5.8869	0.001184
2	Imidazolone A	1.4534	-	pos	301.1513055	0.029822993	4.2341	1.914E-5
3	5-(2'-Carboxyethyl)-4,6-Dihydroxypicolinate	1.5172	C05655	pos	245.0773643	0.006904777	4.214	2.411E-5
4	PERILLIC ACID	6.408	C11924	pos	167.1069348	0.007771743	4.1893	0.04785
5	TIAZOFURIN	2.0175	C22196	pos	261.0545457	0.027450686	4.1469	0.0001215
6	Pro-Pro-Pro	2.0333	-	pos	310.1769838	0.005335343	4.0258	8.086E-5
7	Omega-hydroxyfinasteride	2.387	-	pos	397.2459653	0.049968554	3.9778	2.352E-6
8	LysoPE(22:5(4Z,7Z,10Z,13Z,16Z)/0:0)	6.6297	-	pos	550.2924378	0.022672949	3.8815	6.647E-6
9	Glucose pyruvate acetate	2.538	-	pos	307.0657252	0.009280368	3.7186	3.949E-6
10	SM(d18:2(4E,14Z)/18:1(9Z)-O(12,13))	6.6137	-	pos	804.5552393	0.054226213	3.6751	1.298E-8
11	7-Aminomethyl-7-carbaguanine	2.7291	C16675	pos	180.0884	0.010978507	3.6734	1.651E-5
12	Gentiopicroside	2.7291	C09782	pos	398.1431097	0.040342164	3.6703	6.933E-7

13	Xanthylic acid	2.7894	C00655	pos	382.078125	0.016415662	3.6028	3.775E-6
14	Amastatin	2.8133	C01552	pos	439.2568695	0.051636375	3.5727	1.864E-9
15	Withanolide B	2.8213	C00828	pos	499.2417952	0.091361641	3.5441	0.0001266
16	Lactacystin	2.8293	-	pos	394.1654068	0.029225202	3.5421	1.939E-10
17	Doxazosin	2.8363	C06970	pos	474.1751761	0.030028099	3.5274	3.835E-6
18	Campesterol glucoside	2.8843	-	pos	304.200768	0.03573893	3.4932	6.575E-7
19	2-(3-Carboxy-3-(methylammonio)propyl)-L-histidine	2.8992	C04692	pos	304.1765147	0.048218526	3.4582	2.833E-5
20	Levamisole	2.9097	C07070	pos	472.1594102	0.019464579	3.446	1.809E-6
21	LysoPE(P-18:0/0:0)	6.9792	-	pos	488.3128517	0.007550336	3.4424	0.002264
22	S-Butylcysteine sulfoxide	3.0518	-	pos	176.07449	0.010672736	3.3719	3.752E-5
23	Gly Phe	3.0759	-	pos	223.1083982	0.048057269	3.3635	2.875E-6
24	5-hydroxyindole thiazolidine carboxylate	3.0839	-	pos	279.0805868	0.00131669	3.3375	3.651E-8
25	Ethyl crotonate	3.2513	-	pos	246.1705784	0.043434825	3.3342	4.952E-6
26	Asp Pro Ile	3.3652	-	pos	344.1826446	0.030207123	3.3296	7.412E-6
27	PC(18:1(9Z)/18:2(9Z,12Z))	6.7968	C00157	pos	806.570532	0.186948573	3.3171	0.0002908
28	Taurohyocholate	6.4002	C15516	pos	480.2797086	0.005120765	3.3041	5.706E-5
29	1-Nitro-7-hydroxy-8-glutathionyl-7,8-dihydronaphthalene	4.1085	C14803	pos	497.1356731	0.051310671	3.2713	0.0005294
30	(S)-Oleuropeic acid	4.4137	-	pos	226.1443319	0.022500419	3.2661	1.955E-5
31	Indole-3-Carbinol	4.4519	-	pos	130.0654844	0.051322207	3.2089	5.971E-7
32	Bile acid	6.3842	C01558	pos	373.2746794	0.027654453	3.1771	3.734E-6

33	7-Ketolithocholic Acid	6.2657	-	pos	373.275023	0.043403303	3.1723	5.8E-7
34	Hexenoylcarnitine	4.7603	-	pos	240.1599588	0.109404837	3.1505	5.743E-5
35	PGP(i-21:0/PGD2)	4.8837	-	pos	933.5274411	0.023007425	3.1437	5.571E-7
36	Isovalerylcarnitine	6.2103	C20826	pos	246.1705936	0.010731279	3.0828	0.02151
37	Geranic Acid	5.0627	C16461	pos	186.1492498	0.021801351	3.0501	6.72E-5
38	N-Acetyltyrosine	5.3411	C01657	pos	206.0816376	0.022866343	3.0159	1.343E-5
39	Myosmine	5.7612	C10160	pos	310.2020963	0.053054423	2.9435	0.0004634
40	Anatabine	6.016	C10126	pos	338.2335071	0.060501209	2.9412	0.0001424
41	Prednimustine	6.064	C19512	pos	628.2530801	0.013698684	2.9146	0.0006364
42	N-Acetyl-D-glucosaminyldiphosphodolichol	6.0705	C04500	pos	570.2208439	0.047875859	2.9005	4.634E-6
43	Dihydrozeatin-O-glucoside	6.118	C16448	pos	425.2161242	0.022484113	2.899	2.188E-6
44	3beta-hydroxy-4beta-methyl-5alpha-cholest-7-ene-4alpha-carbaldehyde	6.1642	C11509	pos	470.3970333	0.029054668	2.8906	4.926E-6
45	(4Z,7Z,10Z,13Z,16E,18E)-20-Hydroxydocosa-4,7,10,13,16,18-hexaenoylcarnitine	6.2342	-	pos	470.3278823	0.062282839	2.8906	6.256E-5
46	Sphingosine	6.2657	C00319	pos	300.2904968	0.003670877	2.8896	1.252E-5
47	Sphinganine	6.2974	C00836	pos	302.3060689	0.073366316	2.8471	0.000179
48	(-)-Dihydrocarveol	6.3133	C11396	pos	309.2795037	0.04963212	2.8466	0.0008778
49	Oxandrolone	6.3842	C07346	pos	339.2516243	0.060412637	2.838	0.0002873
50	(R)-6'-O-(4-Geranyloxy-2-hydroxycinnamoyl)-marmin	6.5109	-	pos	595.3016616	0.12666056	2.8352	2.175E-5
51	P,P-Dioctyldiphenylamine	6.5347	-	pos	394.348002	0.002520408	2.8253	2.115E-5
52	GPCho(22:5/16:0)	6.6217	C00157	pos	830.5707489	0.167550954	2.7594	0.0002761

53	PC(22:6(4Z,7Z,10Z,13Z,16Z,19Z)/P-18:0)	6.7489	C00157	pos	818.6102335	0.184339654	2.7388	0.0002602
54	3-Dehydroteasterone	6.7888	C15792	pos	510.3575653	0.010528747	2.7156	1.944E-8
55	PC(P-16:0/3:0)	6.7968	-	pos	558.3552602	0.069478775	2.7066	0.0002699
56	PC(18:1(9Z)e/2:0)	6.8841	C04598	pos	550.3885838	0.045874748	2.6931	0.00436
57	(S)-Laudanosine	6.9238	-	pos	375.2280262	0.038027372	2.6855	1.153E-5
58	Galabiosylceramide (d18:1/16:0)	7.13	C06126	pos	900.5744581	0.005530616	2.6735	0.0003055
59	PE(22:0/PGJ2)	7.2652	-	pos	876.5655334	0.016489027	2.6472	0.002341
60	Rolipram	7.7193	-	pos	568.3418093	0.089323591	2.6358	0.002469
61	855527-92-3 (R-Isomer)	7.6076	-	pos	623.2360665	0.013637105	2.6293	0.003007
62	PS(22:6(5Z,8E,10Z,13Z,15E,19Z)-2OH(7S, 17S)/22:2(13Z,16Z))	7.5916	-	pos	964.5309814	0.006435925	2.6103	1.191E-5
63	PS(15:0/22:2(13Z,16Z))	7.2652	C02737	pos	824.5425047	0.02223058	2.6053	0.002591
64	PC(18:3(9Z,12Z,15Z)/20:1(11Z))	7.13	C00157	pos	810.6041333	0.063685694	2.578	4.367E-6
65	N-Oleoyl Cysteine	6.8841	-	pos	368.2629622	0.063145038	2.5737	1.126E-5
66	Benzoylnorecgonine	6.8525	-	pos	592.2671293	0.001223035	2.5709	9.987E-5
67	LysoPE(18:1(11Z)/0:0)	6.8206	-	pos	462.2968822	0.074231364	2.5654	0.001094
68	LysoPE(0:0/22:4(7Z,10Z,13Z,16Z))	6.6773	-	pos	530.3246963	0.010563476	2.558	0.0005637
69	(3beta,17alpha,23S)-17,23-Epoxy-3,29-dihydroxy-27-norlanosta-7,9(11)-diene-15,24-dione	6.6217	-	pos	509.2661914	0.01802967	2.5214	9.368E-5
70	CAY10444	6.6217	-	pos	288.199756	0.012502556	2.5186	6.182E-8
71	PC(18:1(9Z)/20:4(8Z,11Z,14Z,17Z))	6.6057	C00157	pos	808.5885976	0.251761157	2.5161	3.442E-6
72	Mycobactins	6.5977	-	pos	614.2468378	0.010025833	2.4989	0.003451

73	LysoPC(18:2(9Z,12Z)/0:0)	6.5817	-	pos	1039.67623	0.012359342	2.4962	4.047E-5
74	PC(2:0/18:1(12Z)-2OH(9,10))	6.5347	-	pos	634.3117724	0.018069367	2.4664	0.0001613
75	Australine	2.625	C10132	pos	190.1077679	0.021173301	2.4517	0.0002137
76	Benzoylmesaconine	6.408	-	pos	628.2530386	0.007116451	2.4505	0.0008228
77	N-Linoleoyl Histidine	6.4002	-	pos	462.2689603	0.0071444	2.4472	0.001601
78	Sebacic Acid	6.3922	C08277	pos	203.1281429	0.011821192	2.4245	4.131E-5
79	Cervonoyl ethanolamide	6.3765	C13828	pos	355.2643097	0.003844348	2.3951	0.01273
80	Cerebronic acid	6.337	C17873	pos	402.3955914	0.039572389	2.3641	0.008899
81	Oleylcarnitine	6.329	-	pos	394.3693072	0.069296868	2.3558	0.001991
82	PA(i-24:0/i-13:0)	6.3133	C00416	pos	782.5616347	0.12609812	2.3438	0.001152
83	22-Hydroxydocosanoic acid	6.2737	C19623	pos	374.3641181	0.013719413	2.3227	0.0004731
84	Oleamide	6.242	C19670	pos	314.3060836	0.009657914	2.3194	0.02338
85	7a,17-dimethyl-5b-Androstane-3a,17b-diol	6.2103	-	pos	338.3063479	0.014608354	2.319	0.009361
86	Cyclohexaneundecanoic acid	6.1707	C12100	pos	286.27482	0.081341918	2.283	3.322E-5
87	Calcidiol	6.0943	C01561	pos	442.3656538	0.026209908	2.2739	0.0123
88	Taurohyocholic Acid	6.0705	C15516	pos	480.2799238	0.010700885	2.2728	0.004238
89	N-Eicosapentaenoyl Histidine	6.0705	-	pos	462.2689993	0.007892253	2.2648	0.001259
90	Dibutyryl adenosine	6.048	-	pos	425.2160874	0.166216712	2.2581	0.02438
91	Ubiquinone-2	5.9441	C00399	pos	336.2179307	0.049417291	2.2543	9.04E-5
92	Tenuazonic acid	4.9362	C08511	pos	230.1393828	0.033947997	2.2372	0.002021
93	Swainsonine	4.9028	C10173	pos	174.1128532	0.042565715	2.201	5.91E-5

94	Bremelanotide	4.8682	-	pos	513.268987	0.064688565	2.1982	0.000667
95	Loliolide	4.6299	-	pos	238.1443122	0.044749198	2.1949	0.0004116
96	Beta-Thujaplicin	4.595	C09904	pos	165.0913345	0.027872683	2.1924	0.0008145
97	2,4(1H,3H)-Pyrimidinedione, 5-fluoro-1-(tetrahydro-2-furanyl)-, (R)-	4.4519	C12673	pos	233.0926113	0.015660401	2.1803	0.004955
98	(3Z)-2-Propylpent-3-enoic acid	4.4137	C16654	pos	160.1335177	0.022723826	2.1748	0.01959
99	6-Hydroxynon-4-enoylcarnitine	4.3439	-	pos	316.2129395	0.029277702	2.1737	0.000122
100	NNAL-N-glucuronide	4.2602	C19606	pos	428.1909975	0.057428012	2.1502	2.277E-5
101	N-butanoyl-lhomoserine lactone	4.1441	-	pos	172.0971545	0.011720422	2.139	0.04018
102	Glutamyltryptophan	3.999	-	pos	334.1409367	0.038666022	2.1161	0.00862
103	Gamma-Aminobutyryllysine	3.999	-	pos	276.1272071	0.029071795	2.1147	0.0003944
104	S-Prenyl-L-cysteine	3.7408	C06751	pos	190.0903097	0.045269428	2.1006	0.02103
105	L-Dopa	3.7153	C00355	pos	180.0658893	0.016559496	2.0848	0.00576
106	Dithizone	3.6775	-	pos	274.1115343	0.037647786	2.079	0.004046
107	Glycerophosphocholine	0.611	C00670	pos	258.1106737	0.010463198	2.0789	0.0007317
108	Metazachlor	3.5967	C10948	pos	295.1296969	0.019890637	2.0751	1.666E-5
109	Gamma-Glu-leu	3.4766	-	pos	261.1451949	0.012758091	2.0646	0.0001557
110	(2E,7E)-Nona-2,7-dienedioylcarnitine	3.4428	-	pos	360.2027257	0.086013742	2.062	0.0168
111	Xanthurenic Acid	3.3652	C02470	pos	206.0453222	0.010976976	2.0521	0.01465
112	Acetyl-L-tyrosine	3.2659	C01657	pos	224.0923565	0.037807441	2.0477	0.006361
113	Riboflavin (Vitamin B2)	3.8737	-	pos	377.1470854	0.010951503	2.0456	0.0007638

114	6-Hydroxyoct-3-enedioylcarnitine	2.749	-	pos	332.1716343	0.024764815	2.0399	0.006887
115	Ile Glu Glu	2.7291	-	pos	390.1883427	0.016004787	2.0391	0.01355
116	Caryophyllen-beta	2.682	-	pos	290.1605673	0.013804345	2.0379	0.005965
117	Coumaryl acetate	2.6741	C20465	pos	210.1130254	0.041737726	2.0325	0.0001852
118	P-Coumaraldehyde	2.6012	C05608	pos	190.0866753	0.066760315	2.0307	0.007387
119	4-Benzofuranacetamide, N-methyl-N-(7-(1-pyrrolidinyl)-1-oxaspiro(4.5)dec-8-yl)-	2.387	-	pos	199.1264556	0.029553082	2.0193	0.001518
120	3-Hydroxyisovalerylcarnitine	2.0175	-	pos	262.1655515	0.005144065	2.0082	0.00733
121	N-Acetyl-a-neuraminic acid	0.6734	C19909	pos	310.1140605	0.030008482	1.9931	0.0007537
122	Timonacic	1.6286	-	pos	134.0272319	0.021525982	1.9896	0.01255
123	(2S,3R)-3-hydroxy-2-methylpentanedioylcarnitine	1.5651	-	pos	306.1556231	0.059910568	1.9884	0.001529
124	2,4-Thiazolidinedicarboxylic acid, 2-methyl-	0.96	-	pos	192.0328739	0.012390468	1.9804	0.0004671
125	Pyroglutamic Acid	0.8882	C01879	pos	130.0502689	0.002935899	1.9776	0.003143
126	N-(2-Hydroxy-3,3,3-trifluoropropyl)-2-(2-nitro-1-imidazolyl)acetamide	0.7923	-	pos	247.0430279	0.017096563	1.9694	0.02156
127	Niflumic Acid	0.6971	C13698	pos	327.0323604	0.018926675	1.9561	7.858E-5
128	Diaminopimelic acid	0.6892	C00666	pos	173.0924762	0.02681507	1.9511	0.004797
129	Homocysteine thiolactone	0.6892	-	pos	100.0220851	0.009523986	1.9495	0.0005783
130	Asp-Glu	0.6812	-	pos	263.0879517	0.01376797	1.9448	0.00236
131	2-Aminoadipic acid	0.6734	C00956	pos	144.0658576	0.006729482	1.9366	0.01019
132	Octopine	0.6656	C04137	pos	247.1404892	0.010735557	1.9335	0.001189
133	(E,E)-Trichostachine	0.6419	C10174	pos	335.1358226	0.00463336	1.9267	0.04688

134	N,N-Dimethylarginine	0.6419	C03626	pos	203.1507266	0.002230314	1.9171	0.007383
135	2-Amino-4-oxopentanoic acid	0.6187	C03341	pos	132.0658766	0.031468344	1.9162	4.467E-5
136	Calystegine B2	0.6031	C10851	pos	389.1332221	0.007322942	1.8944	0.03509
137	D-Galactosamine	0.5878	C02262	pos	162.0763931	0.163647334	1.885	0.009587
138	Lysinoalanine	0.5222	-	pos	234.145325	0.000463118	1.8749	0.00138
139	4-Amino-1-butanol	0.4983	-	pos	72.08130533	0.004076993	1.8656	0.004477
140	3-Hydroxy-C10-Homoserine Lactone	6.3053	-	pos	272.1863077	0.085827301	1.8638	0.002608
141	LysoPE(16:1(9Z)/0:0)	6.5739	-	pos	452.2786727	0.019629361	1.8492	0.00157
142	PC(22:5(4Z,7Z,10Z,13Z,16Z)/18:3(9Z,12Z,15Z))	7.3605	C00157	pos	852.5559926	0.016224518	1.8488	0.008273
143	4-(2,6,6-Trimethyl-1-cyclohexen-1-yl)-2-butanone	6.8841	C03527	pos	212.201262	0.012209705	1.843	0.0002967
144	GPEtn(16:1/22:4)	6.7091	C00350	pos	788.5234653	0.019124358	1.8355	9.559E-5
145	LysoPC(16:1(9Z)/0:0)	6.5502	C04230	pos	494.325818	0.00497859	1.8234	0.004501
146	N-Glycolylneuraminic acid	0.6419	C03410	pos	290.087667	0.024451219	1.8212	0.001557
147	3-ketosphingosine	6.2183	C06121	pos	280.2642301	0.013591493	1.8154	0.004534
148	2-Hydroxy-4-[(1R)-1-hydroxy-8-methyl-6-oxononyl]-3-methyl-2H-furan-5-one	6.1944	-	pos	307.1522845	0.0112384	1.8105	4.174E-5
149	Glutamylglutamic acid	0.6892	C01425	pos	277.1037615	0.009526557	1.8099	0.0009432
150	Lunatone	1.0079	-	pos	371.1140931	0.007753729	1.807	0.002787
151	[(2R,3R,4R)-3,4,5-Trihydroxy-1-oxopentan-2-yl] (2R)-2-amino-3-sulfanylpropanoate	0.6734	-	pos	254.0697877	0.006166521	1.8066	0.01826
152	3,4-Dimethyl-1,2-cyclopentanedione	0.5222	-	pos	144.1022782	0.018551251	1.7926	0.001639
153	Saccharopine	0.5381	C00449	pos	309.1663634	0.045576268	1.7848	0.03692

154	Epsilon-(Carboxymethyl)lysine	0.5958	-	pos	205.1187254	0.022714139	1.782	0.01156
155	L-Carnitine	0.6187	C00318	pos	162.1127674	0.004041915	1.7673	0.000459
156	D-Mannose 6-Phosphate	0.6341	C00275	pos	261.0375686	0.027595667	1.7586	4.534E-5
157	L-Cysteine	0.6419	C00097	pos	122.0274089	0.028189638	1.7503	0.0003598
158	G-Nitro-l-arginine methyl ester	0.6419	-	pos	198.0976632	0.067343947	1.7389	0.02733
159	L-2-Amino-3-oxobutanoic acid	0.6498	C03508	pos	235.0928606	0.030914605	1.7205	0.002719
160	4-Hydroxy-L-Proline	0.6578	C01157	pos	114.0554998	0.015664817	1.7148	0.008159
161	5-Methyldeoxycytidine	0.6578	C03592	pos	259.140623	0.008309869	1.7069	0.005866
162	Gamma-Glutamyl-beta-(isoxazolin-5-on-2-yl)alanine	0.6578	-	pos	319.1256187	0.031606383	1.7063	0.005496
163	Linatine	0.6971	C05939	pos	301.1513267	0.067358036	1.7062	0.0001797
164	Thiodiacetic acid	0.7208	C14872	pos	192.0328836	0.00457587	1.7007	1.064E-5
165	TWS119	0.96	-	pos	382.1277469	0.048043907	1.7002	0.03549
166	Thr Ala Lys	0.968	-	pos	319.1985729	0.00731062	1.6869	0.004521
167	(R)-(+)-2-Pyrrolidone-5-carboxylic acid	1.254	-	pos	130.0502942	0.00293041	1.6845	0.0003229
168	Leucyl-Aspartate	1.1188	-	pos	247.1294015	0.006243942	1.6751	0.0004279
169	Acetylcysteine	0.6892	C06809	pos	146.0272972	0.011964592	1.6721	0.005333
170	Allysine	0.5301	C04076	pos	163.1079609	0.006311599	1.667	0.0005573
171	Benzoic Acid	3.7153	C00261	pos	105.0339898	0.054839332	1.6588	4.189E-5
172	Dulcitol	0.6419	C01697	pos	183.0866447	0.034216771	1.6555	0.0002002
173	L-Cystine	0.5958	C00491	pos	241.0315596	0.002600978	1.6542	0.0008127
174	Sulfolithocholylglycine	6.0705	C11301	pos	536.2677409	0.008830167	1.6466	8.043E-5

175	Trans-Cinnamic Acid	5.3411	C00423	pos	131.0495262	0.030462869	1.6448	0.0004858
176	Acetamidopropanal	1.0198	C18170	neg	275.1253114	0.026150436	1.6354	0.02632
177	2-(Chloromethyl)-4-(4-nitrophenyl)-1,3-thiazole	1.0494	-	neg	234.9758839	0.00579403	1.6299	0.0004277
178	Aspartyl-Leucine	1.1279	-	neg	245.1144188	0.023166288	1.6289	0.002273
179	N-Formyl-L-glutamic acid	1.2421	C01045	neg	196.0223605	0.004322785	1.6277	3.122E-5
180	2-Amino-3-phosphonopropionic acid	1.689	C05672	neg	205.9628806	0.01002227	1.6068	0.01872
181	Isopropylmaleic acid	2.1163	C02631	neg	203.0558173	0.073126839	1.6059	0.001003
182	N-Acetylgalactosamine	2.1581	C01074	neg	202.071808	0.047583175	1.603	0.007237
183	Galactosylglycerol	2.607	C05401	neg	235.0823588	0.012435447	1.6028	0.02286
184	Pirprofen	2.6244	-	neg	288.0184457	0.008664151	1.6002	0.0006658
185	4-Methoxyindoxyl sulfate	2.7336	-	neg	244.0286285	0.059417665	1.5996	0.009074
186	(2E,6E,8E)-Dodeca-2,6,8-trienedioylcarnitine	2.7336	-	neg	388.1733495	0.011874049	1.5958	0.03036
187	N-Formyl-L-Methionine	2.7734	C03145	neg	176.0382327	0.01307354	1.5953	0.02851
188	Suberylglycine	2.799	-	neg	230.1033433	0.022245411	1.5936	0.0004464
189	1-O-Feruloyl-beta-D-glucose	0.6597	C17759	neg	377.0858643	0.012528613	1.5923	0.04929
190	10-Formyldihydrofolate	2.9068	C03204	neg	470.1441021	0.024710272	1.5837	0.0008424
191	3,4-Methyleneazelaic acid	3.0999	-	neg	483.2219599	0.024795246	1.5833	6.549E-5
192	N-Methylglutamic acid	0.6753	C01046	neg	160.0609694	0.004576623	1.5767	0.00493
193	Glutamic acid diethyl ester	3.1482	-	neg	202.1082502	0.011380675	1.5747	0.008162
194	Fe(II)-nicotianamine	3.2188	-	neg	302.136099	0.010337825	1.5704	0.04947
195	2-Hydroxy-3-Methylbutyric Acid	3.2288	-	neg	117.0550488	0.017135345	1.5701	0.003144

196	N-Acetyl-L-Tyrosine	3.2595	C01657	neg	222.0771095	0.00840976	1.5616	0.02282
197	Glycyl-D-proline	0.6907	-	neg	217.0828254	0.00821479	1.5584	0.0003583
198	4,6-Dihydroxy-2-quinolinecarboxylic acid	3.3581	C08480	neg	204.0299719	0.011356809	1.5562	0.005454
199	L-Acetylcarnitine	3.4119	C02571	neg	202.1082843	0.015662529	1.5536	0.02147
200	Phenyl glucuronide	3.4909	-	neg	269.0670706	0.003416956	1.5524	0.0007102
201	3,4,5,6-Tetrahydrohippuric acid	3.5396	-	neg	228.0876939	0.025523332	1.5431	0.001357
202	Glutamine-glutamate	0.6907	-	neg	290.0996661	0.016911827	1.5273	0.0003464
203	Hippuric Acid	3.7184	C01586	neg	178.0505062	0.002582239	1.5238	0.001721
204	Trifluoroacetyl-l-lysyl-l-alaninanilide	4.2593	-	neg	423.1454013	0.041394177	1.5166	0.001694
205	Glycocholenate sulfate	4.2628	-	neg	524.2359823	0.035424866	1.5127	0.0195
206	Nifekalant	4.4146	-	neg	404.1934922	0.01231268	1.5111	2.267E-5
207	6-Hydroxyhexanoic acid	4.5601	C06103	neg	131.0707297	0.00441802	1.5047	0.04492
208	2-Hydroxy-2,6,6-trimethylcyclohexanone	4.6013	-	neg	201.112929	0.018759598	1.5034	0.006602
209	Ac-Tyr-OEt	4.8341	C01657	neg	250.1087309	0.021591245	1.4991	0.0001792
210	Gamma-D-Glutamylglycine	0.6597	-	neg	203.067031	0.005801443	1.4984	0.004549
211	ACEXAMIC ACID	4.899	-	neg	172.0974167	0.003009362	1.4964	0.01043
212	Norhygrine	5.083	-	neg	172.0974546	0.008311979	1.4948	0.004917
213	Dihydroferulic acid 4-O-sulfate	5.083	-	neg	275.0212455	0.002404362	1.4943	0.0005514
214	1-Octen-3-yl glucoside	5.1828	-	neg	335.1717326	0.013932333	1.4925	0.01712
215	Indolelactic acid	5.3381	C02043	neg	204.0663498	0.006404523	1.4868	0.005237
216	Sepiapterin	5.3381	C00835	neg	272.0543759	0.007443844	1.4608	0.01123

217	Disoxaril	5.4556	C06496	neg	363.1666736	0.047695895	1.4586	0.002633
218	2-Amino-3-cyclohexylpropanoic acid	5.6425	-	neg	216.1239848	0.01589033	1.4584	0.02254
219	11,13-Dihydrotaraxinic acid glucosyl ester	5.758	-	neg	425.1826266	0.062247271	1.4559	0.0414
220	(Z)-4',6-Dihydroxyaurone	5.8944	C08644	neg	299.0564155	0.035609847	1.449	0.03293
221	(R)-Pelletierine	5.9125	-	neg	186.1131293	0.029553313	1.4481	0.0003136
222	4-Heptenoic acid	5.9444	-	neg	255.1603691	0.020706614	1.4463	0.006976
223	Tsangane L 3-glucoside	5.9652	-	neg	373.2236605	0.017142841	1.445	0.01992
224	4-O,6-O-Benzylidene-alpha-D-glucopyranose	5.9821	-	neg	289.067302	0.026928688	1.436	0.005074
225	4-oxo-Retinoic acid	6.0755	C16678	neg	359.1867266	0.011559255	1.4351	0.02321
226	Lovastatin acid	6.0755	C21130	neg	421.2604899	0.010745039	1.4291	0.01256
227	19(S)-HETE	6.0834	C14749	neg	365.2336144	0.010823968	1.4256	0.0004041
228	(3S,7E,9S)-9-Hydroxy-4,7-megastigmadien-3-one 9-glucoside	6.0907	-	neg	415.1982656	0.018076795	1.4232	0.02895
229	Jasmolone	6.1129	-	neg	225.1131221	0.007595469	1.4183	0.002139
230	Arginylglycine	6.1297	-	neg	266.1012657	0.034711911	1.4139	0.001247
231	L-Menthyl acetoacetate	6.1536	-	neg	285.171028	0.046495893	1.4102	0.01941
232	10,11-dihydro-20-dihydroxy-LTB4	6.1668	-	neg	415.234502	0.009844629	1.4096	0.0487
233	18-HEPE	6.1836	C18177	neg	363.2182716	0.014169567	1.4091	0.001989
234	MG(0:0/20:4(5Z,8Z,11Z,14Z)/0:0)	6.2112	C13856	neg	423.2762138	0.016230646	1.4062	0.002019
235	3a,6b,7a,12a-Tetrahydroxy-5b-cholanoic acid	6.2458	C01094	neg	405.2653505	0.008813143	1.3994	0.01822
236	Sulfocholic Acid	6.2512	-	neg	487.2381376	0.037967265	1.3991	0.0231

237	Goshuyic acid	6.3361	-	neg	269.1761406	0.050474594	1.3861	0.02472
238	3-Hydroxy-6,8-dimethoxy-7(11)-eremophilen-12,8-olide	6.3361	-	neg	291.1586366	0.030792102	1.3851	0.0006964
239	Valproic acid	6.3677	C07185	neg	287.2231418	0.059256945	1.3768	0.02569
240	Cannabidiol	6.3677	C07578	neg	359.2232469	0.014602987	1.3719	0.0001408
241	Cholic Acid	6.3776	C00695	neg	407.2810182	0.016412202	1.3707	0.04922
242	Ergocornine	6.4021	C09162	neg	582.2734189	0.006995465	1.3694	0.0005194
243	6-Hydroxypentadecanedioic acid	6.4668	-	neg	309.1687238	0.046977734	1.359	0.01556
244	10-hydroxy-11S,12S-epoxy-5Z,8Z,14Z-eicosatrienoic acid	6.4781	C14810	neg	317.2124394	0.00948387	1.3559	0.0005253
245	Deoxycholic Acid	6.5393	C04483	neg	391.2857611	0.050609312	1.3502	0.01064
246	PE(16:1/0:0)	6.5745	-	neg	450.2632552	0.015154649	1.3486	0.0001458
247	Scyphostatin	6.6303	-	neg	466.2967243	0.060818168	1.3462	0.003602
248	Leu-Arg-Asn-Arg	6.6303	-	neg	594.2830552	0.033745386	1.3446	0.01952
249	Cholesterol glutamate	6.6738	-	neg	550.3530215	0.039261015	1.3362	0.024
250	12,13-EpOME	6.7351	C14826	neg	295.2280694	0.025302038	1.3333	0.04718
251	Spiroxamine	6.7746	C11124	neg	342.2653254	0.004525356	1.3326	0.004914
252	1-(4-O-beta-D-glucopyranosyl-3-methoxyphenyl)-3,5-dihydroxydecane	6.2656	-	neg	453.2867801	0.006324493	1.332	0.02121
253	(6S,8Z)-6-Hydroxy-3-oxotetradecenoic acid	6.2946	-	neg	255.1603602	0.002758438	1.326	0.01673
254	4,11,13,15-Tetrahydroridentin B	6.3103	-	neg	267.1604831	0.01119706	1.3084	0.003558
255	Corchoroside B	7.735	-	neg	553.2566448	0.030699564	1.3078	0.002024
256	Porrigenin A	7.735	-	neg	485.2686854	0.02912621	1.3003	0.001185

257	Pubescenol	7.6073	-	neg	509.2684748	0.008540529	1.3003	0.0003166
258	7,10,13,16,19-Docosapentaenoic acid	6.9169	C16513	neg	329.24901	0.023694081	1.2802	0.0003452
259	N-Palmitoyl Glycine	6.8063	-	neg	312.2548243	0.051855091	1.2657	0.01862
260	Linoleoyl ethanolamide	6.7944	C13828	neg	368.2810536	0.043242171	1.2635	0.01717
261	1-Heptadecanoylglycerophosphoethanolamine	6.7885	-	neg	466.2942465	0.159347163	1.2609	0.01663
262	LysoPC(17:0/0:0)	6.7831	C04230	neg	554.3476611	0.018529705	1.2567	0.04024
263	N-Docosahexaenoyl Methionine	6.701	-	neg	458.2744183	0.005753107	1.2547	0.007471
264	PE(22:5/0:0)	6.6303	-	neg	526.2951223	0.006350853	1.253	0.04631
265	Dihydrocortisol	6.4781	C05471	neg	385.2000968	0.03348979	1.2488	0.0006132
266	2-Isopropyl-5-methylphenol acetate	6.4085	C09909	neg	429.2265819	0.020749602	1.2404	0.02474
267	Physangulide	6.3776	-	neg	543.2564713	0.01666479	1.2397	0.04045
268	2,4-dimethylhexanedioylcarnitine	6.3025	-	neg	338.1590164	0.014897691	1.2392	0.02747
269	3-Dehydrocholic Acid	6.2862	-	neg	451.2711488	0.014794744	1.2247	0.04843
270	Dihydrotestosterone diglucuronide	6.2656	-	neg	679.2319948	0.017104642	1.2147	0.004628
271	Leupeptin	6.2656	C01591	neg	407.2810865	0.014310192	1.2132	0.03387
272	4-Heptyloxyphenol	6.2656	C14236	neg	253.1446689	0.015576297	1.2052	0.01323
273	L-Histidine trimethylbetaine	6.194	C05575	neg	393.2290389	0.012228508	1.2034	0.001416
274	4-Hydroxyretinoic acid	6.107	C16677	neg	361.2026171	0.018308586	1.2028	0.01051
275	2-Octynoic acid	6.0907	-	neg	325.163741	0.010571152	1.1995	0.01403
276	4-(3-Hydroxybutyl)-3,3,5-trimethylcyclohexanone	6.0907	-	neg	257.1760259	0.016123533	1.1925	0.001841
277	(3S)-3-hydroxycyclocitral	6.0755	C19731	neg	213.112954	0.015295816	1.1915	0.03827

278	1-Arachidonoylglycerol	6.0633	C13857	neg	423.2760279	0.031754376	1.1906	0.002372
279	Dhelwangin	6.0578	-	neg	507.2242507	0.020628158	1.1882	0.0119
280	Haloperidol decanoate	5.9766	-	neg	528.2650598	0.007845473	1.1864	0.03563
281	Captopril-cysteine disulfide	5.9766	-	neg	357.0548213	0.011315392	1.185	0.005014
282	1-Ethenylhexyl butanoate	5.9295	-	neg	197.1543213	0.0455757	1.1835	7.196E-5
283	4-[(2,4-Dihydroxy-3,3-dimethylbutanoyl)amino]butanoic acid	5.9125	-	neg	254.1012363	0.018168129	1.1827	0.004787
284	Liquiritigenin	5.8754	C09762	neg	301.0720364	0.038481813	1.1822	0.0002305
285	O-Aminoazotoluene	5.7841	-	neg	260.0964925	0.05163119	1.1784	0.02812
286	Helenalin	5.7753	C09473	neg	261.1134599	0.074341993	1.1735	0.02853
287	Abscisic acid	5.7131	C06082	neg	263.1291472	0.060519002	1.1685	0.0116
288	5'-O-beta-D-Glucosylpyridoxine	5.6425	C03996	neg	352.0994382	0.089676832	1.1676	0.02706
289	Hymenoxon	5.6425	C09482	neg	263.1291644	0.013390696	1.1659	0.004074
290	Glucosyl (E)-2,6-Dimethyl-2,5-heptadienoate	5.1009	-	neg	361.1510795	0.048869248	1.165	0.00475
291	Tetraphyllin B	5.083	-	neg	308.0732439	0.030613993	1.1626	0.04611
292	R-limonene	5.0661	C06099	neg	229.1080777	0.02431478	1.1604	0.009773
293	Leu-Pro-Ile	4.92	-	neg	378.1775369	0.021233895	1.16	0.0003275
294	3-[[(2S)-2,4-Dihydroxy-3,3-dimethylbutanoyl]amino]propanoic acid	4.899	-	neg	240.0854366	0.015772683	1.1589	0.00223
295	N-Acetyl-D-phenylalanine	4.86	C05620	neg	206.0820408	0.01419813	1.1582	0.01511
296	1H-Imidazole, 2-(2-benzofuranyl)-4,5-dihydro-	4.4538	-	neg	231.0775372	0.015477336	1.1561	0.002584
297	Lupinic acid	4.3513	C01513	neg	228.1241141	0.030756056	1.156	0.008432

298	N-Lactoylvaline	4.1532	-	neg	170.0818496	0.005273142	1.1515	0.001474
299	2-n-Propyl-4-oxopentanoic acid	3.9948	C16655	neg	203.0921982	0.023815333	1.1497	0.0001175
300	Adipate semialdehyde	3.958	C06102	neg	319.1402125	0.002229479	1.1483	0.01815
301	4-Ipomeanol	3.8041	-	neg	213.0766746	0.105911464	1.1451	0.02536
302	Vanilloylglycine	3.7184	-	neg	246.0384948	0.042235474	1.1357	0.02922
303	Cyclohexanecarboxylic acid	3.5942	C09822	neg	173.0814417	0.005330372	1.1347	0.04038
304	3-[4-(sulfooxy)phenyl]propanoic acid	3.4598	-	neg	245.0126954	0.003856504	1.1282	0.00373
305	Epinephrine	3.2555	C00788	neg	228.0877238	0.016353953	1.1251	0.0006821
306	(2R,3R,4R,5R)-2-Amino-4,5,6-trihydroxy-3-[(2R)-1-oxopropan-2-yl]oxyhexanal	3.2065	-	neg	256.0804626	0.007749763	1.1243	0.01257
307	L-Aspartyl-4-phosphate	3.0765	C03082	neg	247.9737557	0.012548164	1.1229	0.0129
308	Agar	3.0624	C08815	neg	335.1354118	0.014177953	1.1197	0.0005999
309	1-Cyano-2-hydroxy-3-butene	2.9103	-	neg	253.1197308	0.062027105	1.1112	0.03582
310	Pimelylcarnitine	2.8914	-	neg	302.1613958	0.01533409	1.1086	0.005717
311	Gamma-Glutamylfelinylglycine	2.83	-	neg	392.1502916	0.069032722	1.1062	0.006432
312	Butyric Acid	2.7819	C00246	neg	87.04427608	0.011868646	1.1053	0.006004
313	3-Methylcrotonylglycine	2.7685	C20828	neg	202.0718594	0.043510097	1.1001	0.0004446
314	N-Acetylmannosamine	2.5852	C00645	neg	202.0717793	0.022547835	1.0971	0.001164
315	Indican	2.4821	C08481	neg	340.1042197	0.016271689	1.097	0.01082
316	Glutamylleucine	2.0177	-	neg	259.130058	0.073675027	1.0942	0.01185
317	Voglibosa	2.0177	-	neg	248.1139494	0.061399444	1.0934	0.01984

318	H-D-Asp(OtBu)-OH	3.2026	-	neg	188.092403	0.020397268	1.0924	0.0149
319	2,2-Dihydroxy-2-phenylacetic acid	1.3956	-	neg	395.096255	0.030447985	1.0894	0.02172
320	5-Hydroxymethyl-2'-deoxyuridine	1.2632	-	neg	279.0601702	0.011185914	1.0787	0.009936
321	Glucose lactate lactate	1.0274	-	neg	337.0784089	0.081823712	1.0715	0.002653
322	L-Rhamnulose	0.9708	C00861	neg	199.0374213	0.003560689	1.0715	0.004201
323	Diethyl Hydrogen Phosphate	0.6907	C06608	neg	199.03739	0.004459832	1.068	0.01443
324	Stachyose	0.6753	C01613	neg	665.216898	0.008471457	1.064	0.01883
325	(R)-beta-Aminoisobutyric acid	0.6674	C01205	neg	84.04457039	0.03766797	1.0617	0.002647
326	Hydantoin-5-propionic acid	0.6519	C05565	neg	343.0916357	0.007789139	1.0606	0.02876
327	Aspartyl-Gamma-glutamate	0.6519	-	neg	260.0890819	0.031692269	1.0603	0.02257
328	N-(2-hydroxymethyl-3-chloro-4-hydroxyphenyl)anthranilic acid	0.6287	-	neg	314.0192444	0.052814235	1.0583	0.04566
329	Aminofructose 6-phosphate	0.621	C12214	neg	258.0386711	0.008646747	1.0565	0.02111
330	(S)-3-Sulfonatolactate	0.621	C11499	neg	168.9806823	0.016016814	1.0565	0.01097
331	1-[Amino-(3,4-dichloroanilino)methylidene]-2-propan-2-ylguanidine	0.6132	-	neg	286.0605295	0.015021123	1.0563	2.123E-5
332	Glutaminylproline	0.6055	-	neg	288.1204483	0.003244064	1.056	0.0001374
333	N-carboxymethyllysine	0.5821	-	neg	203.10343	0.024480515	1.0497	0.009535
334	(2R)-2-Amino-6-[[(3S,4R,5R)-3,4,5,6-tetrahydroxy-2-oxohexyl]amino]hexanoic acid	0.531	-	neg	307.1515029	0.068064589	1.0493	0.004367
335	Seryllysine	0.5215	-	neg	232.1302433	0.019262655	1.0455	0.0002567
336	2-Hydroxydecanedioic acid	5.2812	-	neg	217.1079858	0.017575524	1.0411	0.01062
337	Azelaic Acid	5.836	C08261	neg	187.0971517	0.013366124	1.0406	0.009816

338	2-Hydroxycampholonic acid	5.9821	-	neg	199.0972477	0.010470279	1.0373	1.794E-5
339	Decanedioic acid	6.043	C08277	neg	201.1129064	0.018251359	1.035	0.001507
340	1-Carboxycyclohexaneacetic Acid	5.5774	-	neg	185.0814948	0.010783409	1.0336	0.005186
341	15-Keto-prostaglandin E2	6.0966	C04707	neg	349.1986337	0.0253405	1.031	0.01784
342	Traumatic Acid	6.1536	C16308	neg	227.1288008	0.031208226	1.0286	0.02752
343	Undecanedioic acid	6.1481	-	neg	215.1286583	0.022284672	1.0239	0.02264
344	DG(2:0/0:0/PGD1)	6.2112	-	neg	469.2816609	0.037835217	1.0231	0.002882
345	H-Hyp-gly-OH	0.6441	-	neg	233.0778454	0.058156938	1.023	0.006487
346	(S)-5-Amino-3-oxohexanoate	0.6441	C03656	neg	289.1409022	0.002680854	1.0221	0.03852
347	2-Amino-3-oxoadipate	0.8274	C05520	neg	196.0224798	0.010419132	1.0209	0.004263
348	Alanine lactate pyruvate	0.8769	-	neg	284.0391847	0.013042032	1.0197	0.0002115
349	8-Hydroxyguanine	0.9515	C20155	neg	166.0364965	0.006244442	1.0182	0.04021
350	1-deoxy-L-glycero-tetrulose 4-phosphate	0.9708	C15556	neg	183.0060183	0.127033524	1.0171	0.008742
351	Artecanin	5.8239	C09302	neg	259.0977599	0.040215478	1.0145	0.000196
352	Methylnoradrenaline	3.4864	C17925;C11768	neg	228.0876862	0.019985103	1.0016	0.003244
353	Phenol sulphate	3.1836	C00850;C02180	neg	172.9908916	0.01032956	1.0011	0.04884
354	Riboflavin	3.8775	C00061	neg	421.1376428	0.014334539	1.0002	0.02716

Supplementary Table S5

Table S5. 262 differential metabolites screened by HLB_vs_Mod.

No.	Metabolite	RT/min	KEGG Compound ID	Mode	M/Z	RSD	VIP	P value
1	Hydroxyprolyl-Valine	1.4374	-	pos	213.1237882	0.002452136	5.9165	0.02758
2	3-Hydroxybutyrylcarnitine	1.4614	-	pos	248.1498233	0.002896189	4.7485	0.0004895
3	Salicyluric acid	2.418	C07588	pos	237.0875253	0.022334163	4.5115	0.001094
4	LysoPE(22:5(4Z,7Z,10Z,13Z,16Z)/0:0)	6.6297	-	pos	550.2924378	0.022672949	4.2894	8.056E-8
5	PE(P-16:0/0:0)	6.7888	-	pos	460.2813747	0.016933309	4.2304	0.0003276
6	Xanthylic acid	2.7894	C00655	pos	382.078125	0.016415662	3.8934	0.002092
7	Lactacystin	2.8293	-	pos	394.1654068	0.029225202	3.8382	9.774E-6
8	2-Lysophosphatidylcholine	6.8841	C04230	pos	546.3554017	0.003626884	3.7415	0.0002134
9	Campesterol glucoside	2.8843	-	pos	304.200768	0.03573893	3.6835	9.347E-5
10	LysoPE(P-18:0/0:0)	6.9792	-	pos	488.3128517	0.007550336	3.6363	0.000312
11	S-Butylcysteine sulfoxide	3.0518	-	pos	176.07449	0.010672736	3.5499	3.327E-6
12	2-Methylhippuric acid	4.0765	C01586	pos	238.0456908	0.024261427	3.4429	0.00317
13	(S)-Oleuropeic acid	4.4137	-	pos	226.1443319	0.022500419	3.3644	0.0003794
14	5-(3E-Pentenyl)tetrahydro-2-oxo-3-furancarboxylic acid	4.5549	-	pos	216.1235169	0.038458679	3.2618	0.008621
15	Hexenoylcarnitine	4.7603	-	pos	240.1599588	0.109404837	3.2038	0.0007197
16	PS(22:6(4Z,7Z,10Z,13Z,16Z,19Z)/22:5(4Z,7Z,10Z,13Z,19Z)-O(16,17))	4.9556	-	pos	934.466149	0.192712255	3.1976	0.0004696
17	Caproic acid	5.0001	C01585	pos	158.1179	0.022236601	3.1309	0.0007334

18	Isovalerylcarnitine	6.2103	C20826	pos	246.1705936	0.010731279	3.0834	0.01911
19	PGP(a-21:0/PGJ2)	5.0918	-	pos	992.5561706	0.027336832	3.0551	0.02425
20	Isopenicillin N	5.1752	C05557	pos	377.1469185	0.020298753	3.0319	0.01403
21	3-methyl-2-Quinoxalinone	5.2186	-	pos	161.0712585	0.067331643	3.0136	0.0293
22	Myosmine	5.7612	C10160	pos	310.2020963	0.053054423	2.9975	0.0003125
23	Umbelliferone	5.9696	C09315	pos	163.0392574	0.014813442	2.9882	0.01908
24	(4Z,7Z,10Z,13Z,16E,18E)-20-Hydroxydocosa-4,7,10,13,16,18-hexaenoylcarnitine	6.2342	-	pos	470.3278823	0.062282839	2.9256	0.02715
25	16,17-Dihydro-16alpha,17-dihydroxygibberellin A7 17-glucoside	6.4794	-	pos	544.2433441	0.045293869	2.9126	0.0004332
26	Retaspimycin	6.5977	-	pos	610.3119238	0.002096369	2.8169	0.004491
27	17beta-Estradiol-3,4-quinone	6.6217	-	pos	636.3276801	0.024793138	2.7768	0.003067
28	PC(22:6(4Z,7Z,10Z,13Z,16Z,19Z)/P-18:0)	6.7489	C00157	pos	818.6102335	0.184339654	2.7551	0.0002678
29	(S)-Laudanosine	6.9238	-	pos	375.2280262	0.038027372	2.7396	0.03913
30	PC(22:6(4Z,7Z,10Z,13Z,16Z,19Z)/P-18:1(9Z))	7.5596	C00157	pos	838.5754633	0.013487279	2.7384	0.04915
31	PC(16:1(9Z)/22:5(4Z,7Z,10Z,13Z,16Z))	7.5916	C00157	pos	806.573452	0.011237462	2.7211	0.005128
32	PC(18:1(11Z)/PGF2alpha)	7.5916	-	pos	896.5432547	0.007922542	2.6997	8.695E-5
33	1-Palmitoylphosphatidylcholine	7.7035	C04230	pos	496.3417892	0.013140005	2.6293	0.01073
34	PS(22:6(5Z,8E,10Z,13Z,15E,19Z)-2OH(7S, 17S)/22:2(13Z,16Z))	7.5916	-	pos	964.5309814	0.006435925	2.6051	0.01226
35	PC(22:6(4Z,7Z,10Z,13Z,16Z,19Z)/16:0)	7.5916	C00157	pos	828.5555526	0.011970175	2.5939	0.0002126

36	N-Alpha-Methylhistamine	7.5676	-	pos	126.1030074	0.056469035	2.5904	0.0303
37	N-Acetyl-D-mannosamine	0.6812	C00645	pos	204.0871215	0.008315363	2.5492	0.009047
38	17beta-Estradiol-2,3-quinone	6.8921	-	pos	614.3429356	0.025906849	2.5464	0.0002697
39	LysoPE(18:1(11Z)/0:0)	6.8206	-	pos	462.2968822	0.074231364	2.5428	0.01877
40	LysoPC(0:0/18:2(9Z,12Z))	6.7968	-	pos	502.3285536	0.026465954	2.5053	0.02217
41	(2-Hydroxy-3-phosphonooxypropyl) octadec-9-enoate	6.6854	-	pos	459.2495923	0.006423583	2.462	0.0002339
42	12(S)-Leukotriene B4	6.6694	C04853	pos	359.2174921	0.009400625	2.4535	0.0009607
43	Dimethylsphingosine	6.4002	-	pos	338.2676263	0.019979334	2.4451	0.0004715
44	PA(i-24:0/i-13:0)	6.3133	C00416	pos	782.5616347	0.12609812	2.4439	0.01092
45	1-[5-(propanoylamino)pyridin-2-yl]-N-propylimidazole-4-carboxamide	2.7291	-	pos	302.1608746	0.017994315	2.4261	0.00554
46	P-Toluenesulfonamide	5.993	C14412	pos	136.0218667	0.021976167	2.3931	0.001666
47	PC(16:0/0:0)	6.6854	C04230	pos	518.3240666	0.002386893	2.3411	0.005409
48	Aspartame	5.2186	C11045	pos	277.1190381	0.061456667	2.3168	0.006026
49	2-Amino-3-(4-hydroxyphenyl)propanal	5.1707	-	pos	166.0866115	0.024150334	2.2946	0.007805
50	Hydrocinnamic acid	5.1676	C05629	pos	192.1022878	0.03985582	2.2945	0.01791
51	Swainsonine	4.9028	C10173	pos	174.1128532	0.042565715	2.2858	0.01571
52	4-Piperidinone, 1-hydroxy-2,2,6,6-tetramethyl-	4.6136	-	pos	172.1335554	0.010128497	2.2811	0.03532
53	Beta-Thujaplicin	4.595	C09904	pos	165.0913345	0.027872683	2.2784	0.03529
54	(3Z)-2-Propylpent-3-enoic acid	4.4137	C16654	pos	160.1335177	0.022723826	2.2643	0.008974
55	6-Hydroxynon-4-enoylcarnitine	4.3439	-	pos	316.2129395	0.029277702	2.2498	0.0004283

56	NNAL-N-glucuronide	4.2602	C19606	pos	428.1909975	0.057428012	2.2435	0.0002754
57	N-lactoyl-Tyrosine	3.5561	-	pos	254.1028997	0.014681897	2.2424	0.02693
58	N-butanoyl-lhomoserine lactone	4.1441	-	pos	172.0971545	0.011720422	2.2337	0.01638
59	Glutamyltryptophan	3.999	-	pos	334.1409367	0.038666022	2.2315	0.01638
60	S-Prenyl-L-cysteine	3.7408	C06751	pos	190.0903097	0.045269428	2.2225	0.004258
61	Gamma-Glu-leu	3.4766	-	pos	261.1451949	0.012758091	2.2002	0.03301
62	4-Hydroxyindole	3.4566	C02040	pos	134.0603619	0.025891253	2.1993	0.01537
63	(2E,7E)-Nona-2,7-dienedioylcarnitine	3.4428	-	pos	360.2027257	0.086013742	2.1919	0.01893
64	Xanthurenic Acid	3.3652	C02470	pos	206.0453222	0.010976976	2.1882	0.02765
65	2-Ethyl-5-methylthiophene	3.0839	-	pos	144.0844108	0.017531553	2.1735	0.02835
66	L-Allysine Ethylene Acetal	3.8857	-	pos	190.1078168	0.001765401	2.1654	0.03981
67	Betaine	0.6264	C00719	pos	118.0867313	0.001469859	2.1539	4.406E-5
68	Secologanate	3.999	C01957	pos	375.131235	0.043494091	2.1155	0.01942
69	Phenylacetylglycine	4.0765	C05598	pos	194.0817639	0.032297048	2.1066	0.01539
70	Tyrosyl-Proline	2.7714	-	pos	279.1347315	0.019351574	2.0952	0.0206
71	Adipic acid	2.757	C06104	pos	188.0922097	0.014724992	2.0707	3.471E-5
72	MG(5-iso PGF2VI/0:0/0:0)	2.682	-	pos	212.1217689	0.021912511	2.07	0.01333
73	P-Coumaraldehyde	2.6012	C05608	pos	190.0866753	0.066760315	2.063	0.001923
74	7-Methylguanosine	2.5619	-	pos	298.1154744	0.025418757	2.043	0.01002
75	1,2-Epithiopropane	2.538	-	pos	166.0727633	0.026690493	2.0419	0.0004444
76	2-Hydroxyquinoline	2.418	C06338	pos	146.0601838	0.017755853	2.0392	0.007103

77	1-Hydroxyisoquinoline	2.0175	C06324	pos	146.0602004	0.020869547	2.0377	0.00151
78	4-Acetylbutyrate	1.7949	C02129	pos	172.0971307	0.023279811	2.0268	0.00635
79	Timonacic	1.6286	-	pos	134.0272319	0.021525982	1.9922	0.03336
80	6-Lactoyltetrahydropterin	1.4374	C04244	pos	204.0871588	0.023055612	1.9873	0.0209
81	2-Mercapto-3-furan-2-ylpropenoic acid	1.4054	-	pos	188.0379953	0.035746483	1.9855	0.04557
82	D-Octopine	1.0238	C04137	pos	247.1405441	0.038768842	1.9611	0.03114
83	(+)-Zeylenol	0.6892	-	pos	385.1300543	0.014155719	1.9591	0.01811
84	Gamma-Glutamylglutamic acid	0.952	C05282	pos	277.1037852	0.062339736	1.9577	0.03032
85	Threonylproline	0.9041	-	pos	249.1450349	0.017724086	1.9458	0.001099
86	Pyroglutamic Acid	0.8882	C01879	pos	130.0502689	0.002935899	1.9424	0.005301
87	Niflumic Acid	0.6971	C13698	pos	327.0323604	0.018926675	1.9326	0.00271
88	Miglitol	0.6892	C07708	pos	249.14511	0.005895578	1.9313	5.113E-5
89	1-Pyrrolidineethanol	0.6892	-	pos	116.1074187	0.024044389	1.9256	0.03412
90	O-Phospho-4-hydroxy-L-threonine	0.6812	C06055	pos	431.043958	0.016536972	1.9062	0.004602
91	Octopine	0.6656	C04137	pos	247.1404892	0.010735557	1.8862	0.03083
92	L-prolinamide	0.6578	C19781	pos	159.0515115	0.014697164	1.8814	0.03451
93	(E,E)-Trichostachine	0.6419	C10174	pos	335.1358226	0.00463336	1.8702	0.02483
94	Valeric acid	0.6419	C00803	pos	120.1023607	0.001930157	1.8641	0.04826
95	D-Galactosamine	0.5878	C02262	pos	162.0763931	0.163647334	1.8607	0.0476
96	Clinprost	0.291	-	pos	329.2485503	0.02612575	1.8573	0.009077
97	3-Hydroxy-C10-Homoserine Lactone	6.3053	-	pos	272.1863077	0.085827301	1.8271	0.02411

98	Cinnamaldehyde	6.321	C00903	pos	133.0651214	0.048404585	1.8186	0.03164
99	LysoPE(0:0/20:5(5Z,8Z,11Z,14Z,17Z))	6.4873	-	pos	500.2790727	0.003266672	1.8164	0.02063
100	PC(20:4(8Z,11Z,14Z,17Z)/16:0)	7.5836	C00157	pos	782.571261	0.0196232	1.8143	0.01766
101	PC(22:5(4Z,7Z,10Z,13Z,16Z)/18:3(9Z,12Z,15Z))	7.3605	C00157	pos	852.5559926	0.016224518	1.807	0.01923
102	PC(22:6(4Z,7Z,10Z,13Z,16Z,19Z)/18:1(11Z))	6.7968	C00157	pos	814.5763449	0.054853393	1.7987	0.004802
103	GPEtn(16:1/22:4)	6.7091	C00350	pos	788.5234653	0.019124358	1.793	0.001872
104	Buprenorphine	6.5267	C08007	pos	468.3100899	0.014682566	1.7801	0.0218
105	Dihyroxy-1H-indole glucuronide I	0.6892	-	pos	358.1144542	0.005068504	1.7785	0.04016
106	Glutamylglutamic acid	0.6892	C01425	pos	277.1037615	0.009526557	1.7779	0.03936
107	Zofenoprilat	1.0079	C21576	pos	358.1145209	0.002107875	1.7776	0.02529
108	Lunatone	1.0079	-	pos	371.1140931	0.007753729	1.7771	0.02796
109	S-Adenosylhomocysteine	1.724	C00021	pos	193.0685242	0.003204864	1.7742	0.01372
110	Norzolmitripan	0.6892	-	pos	282.1202773	0.015556575	1.7524	0.02472
111	Epsilon-(Carboxymethyl)lysine	0.5958	-	pos	205.1187254	0.022714139	1.7454	0.007669
112	N-Lactoylleucine	5.0046	-	pos	204.1235944	0.040571284	1.7426	0.03967
113	L-Carnitine	0.6187	C00318	pos	162.1127674	0.004041915	1.7352	0.005611
114	N-lactoyl-phenylalanine	5.1676	-	pos	238.107988	0.018437856	1.7266	0.04311
115	D-Mannose 6-Phosphate	0.6341	C00275	pos	261.0375686	0.027595667	1.7203	0.002103
116	L-2-Amino-3-oxobutanoic acid	0.6498	C03508	pos	235.0928606	0.030914605	1.7162	0.006552
117	4-Hydroxy-L-Proline	0.6578	C01157	pos	114.0554998	0.015664817	1.7086	3.215E-5

118	Thr Ala Lys	0.968	-	pos	319.1985729	0.00731062	1.706	0.001864
119	(R)-(+)-2-Pyrrolidone-5-carboxylic acid	1.254	-	pos	130.0502942	0.00293041	1.6984	0.03302
120	1-Methyladenosine	1.1347	C02494	pos	282.1202911	0.003178492	1.6893	0.009164
121	4-Aminobutyraldehyde	0.5301	C00555	pos	70.06567138	0.053284516	1.6889	0.01819
122	7-Methylguanine	1.1507	C02242	pos	166.0726091	0.006497708	1.6823	5.045E-5
123	Acetylcysteine	0.6892	C06809	pos	146.0272972	0.011964592	1.6676	0.003742
124	Dodecanoic acid	5.6189	C02679	pos	218.212024	0.013438791	1.6653	0.006181
125	L-Cystine	0.5958	C00491	pos	241.0315596	0.002600978	1.6607	0.005334
126	Salicylic Acid	2.8843	C00805	pos	121.0289933	0.042440607	1.6587	0.002549
127	Nitrobenzodiazepine	0.9936	-	neg	224.0233914	0.014241217	1.65	0.004447
128	N-Formyl-L-glutamic acid	1.2421	C01045	neg	196.0223605	0.004322785	1.641	0.01307
129	5'-Deoxy-5-fluorouridine	1.3878	C12739	neg	267.0375168	0.019411217	1.6379	0.02065
130	Benzo[b]thiophene-2-carboxylic acid	1.5249	-	neg	355.0111761	0.005313113	1.637	0.02143
131	2-(3,4-Dihydroxybenzoyloxy)-4,6-dihydroxybenzoate	1.5313	C04524	neg	305.0318078	0.005646904	1.6328	0.01055
132	Triacetic acid	1.5705	C01757	neg	189.0400729	0.005476649	1.6187	0.02366
133	2-Amino-3-phosphonopropionic acid	1.689	C05672	neg	205.9628806	0.01002227	1.6187	0.02281
134	2-Hydroxybutyric Acid	1.6984	C05984	neg	103.039358	0.030772095	1.6181	0.02611
135	Isopropylmaleic acid	2.1163	C02631	neg	203.0558173	0.073126839	1.6157	0.0341
136	Galactosylglycerol	2.607	C05401	neg	235.0823588	0.012435447	1.6102	0.01342
137	1-O-Feruloyl-beta-D-glucose	0.6597	C17759	neg	377.0858643	0.012528613	1.5984	0.01116

138	4-methoxy-3-(sulfooxy)benzoic acid	2.8548	-	neg	246.9919838	0.018423436	1.5971	0.02064
139	3-Methyladipic Acid	2.8959	-	neg	159.0657509	0.018139885	1.597	0.04634
140	8-Hydroxyquinoline-5-sulfonic acid	3.0009	-	neg	270.0081758	0.01626417	1.5916	0.002159
141	3,4-Methyleneazelaic acid	3.0999	-	neg	483.2219599	0.024795246	1.5861	0.04369
142	2-Hydroxy-3-Methylbutyric Acid	3.2288	-	neg	117.0550488	0.017135345	1.5804	0.04774
143	Glycyl-D-proline	0.6907	-	neg	217.0828254	0.00821479	1.5648	0.04624
144	Indicaxanthin	3.3064	C08549	neg	307.0941016	0.013261879	1.5439	0.02695
145	3-hydroxy-3-(3-hydroxyphenyl)propanoic acid-O-sulphate	3.3503	-	neg	261.0076975	0.01071547	1.5423	0.01162
146	4,6-Dihydroxy-2-quinolinecarboxylic acid	3.3581	C08480	neg	204.0299719	0.011356809	1.5384	0.01964
147	L-Acetylcarnitine	3.4119	C02571	neg	202.1082843	0.015662529	1.5227	0.02314
148	N-lactoyl-Methionine	3.5222	-	neg	220.0648385	0.003730633	1.5217	0.0006656
149	Indoxylsulfuric acid	3.827	-	neg	212.0021013	0.035898325	1.5094	0.002579
150	Narciclasine	4.0815	C08533	neg	328.0419649	0.075271383	1.5084	0.002731
151	Trifluoroacetyl-l-lysyl-l-alaninanilide	4.2593	-	neg	423.1454013	0.041394177	1.5071	0.00237
152	Glycocholenate sulfate	4.2628	-	neg	524.2359823	0.035424866	1.4978	0.001075
153	Nifekalant	4.4146	-	neg	404.1934922	0.01231268	1.4962	0.01372
154	6-Hydroxyhexanoic acid	4.5601	C06103	neg	131.0707297	0.00441802	1.4924	0.0001501
155	Ac-Tyr-OEt	4.8341	C01657	neg	250.1087309	0.021591245	1.4919	0.01786
156	3-Hydroxysebacic Acid	4.8475	-	neg	217.1079788	0.02771317	1.4833	0.01362
157	Gamma-D-Glutamylglycine	0.6597	-	neg	203.067031	0.005801443	1.4829	0.00703

158	ACEXAMIC ACID	4.899	-	neg	172.0974167	0.003009362	1.4797	0.005825
159	1-Octen-3-yl glucoside	5.1828	-	neg	335.1717326	0.013932333	1.4785	0.04035
160	Disoxaril	5.4556	C06496	neg	363.1666736	0.047695895	1.4762	0.0003518
161	2-Amino-3-cyclohexylpropanoic acid	5.6425	-	neg	216.1239848	0.01589033	1.4758	0.001352
162	Cyclodopa glucoside	1.0198	C17751	neg	356.0998731	0.013032435	1.4755	0.0008051
163	(R)-Pelletierine	5.9125	-	neg	186.1131293	0.029553313	1.4707	0.0231
164	4-Heptenoic acid	5.9444	-	neg	255.1603691	0.020706614	1.4679	0.001075
165	Tsangane L 3-glucoside	5.9652	-	neg	373.2236605	0.017142841	1.4639	0.006078
166	4-O,6-O-Benzylidene-alpha-D-glucopyranose	5.9821	-	neg	289.067302	0.026928688	1.4596	0.0356
167	Arginylglycine	6.1297	-	neg	266.1012657	0.034711911	1.4584	0.002322
168	L-Menthyl acetoacetate	6.1536	-	neg	285.171028	0.046495893	1.4584	0.01186
169	Goshuyic acid	6.3361	-	neg	269.1761406	0.050474594	1.452	0.001029
170	PE(20:5/0:0)	6.4918	-	neg	498.2635769	0.006149121	1.451	0.002594
171	LysoPC(14:0/0:0)	6.522	C04230	neg	512.3001548	0.014939678	1.4508	0.01047
172	PE(16:1/0:0)	6.5745	-	neg	450.2632552	0.015154649	1.4463	0.03319
173	Succinic Acid	1.5209	C00042	neg	117.0186747	0.002686754	1.4429	0.006139
174	Leu-Arg-Asn-Arg	6.6303	-	neg	594.2830552	0.033745386	1.4367	0.001848
175	Cholesterol glutamate	6.6738	-	neg	550.3530215	0.039261015	1.4344	0.008711
176	Hydroxybuprenorphine	6.7885	-	neg	504.2720764	0.024904679	1.4341	0.04112
177	PE-NMe(22:6(4Z,7Z,10Z,13Z,16Z,19Z)/18:0)	7.5886	C01241	neg	850.563597	0.014202405	1.4301	0.004826

178	4,11,13,15-Tetrahydroridentin B	6.3103	-	neg	267.1604831	0.01119706	1.4279	0.01696
179	(5Z,8Z,13E,15S)-11,12,15-Trihydroxyicosa-5,8,13-trienoylcarnitine	6.9761	-	neg	532.3027999	0.020815353	1.4101	0.02835
180	5-Hete	6.7538	C04805	neg	301.2176578	0.025834898	1.4101	0.01338
181	PE(20:3/0:0)	6.6381	-	neg	502.2953141	0.009093373	1.3889	0.01401
182	PE(22:5/0:0)	6.6303	-	neg	526.2951223	0.006350853	1.387	0.024
183	1-Stearoylglycerophosphoglycerol	6.5621	-	neg	533.2894866	0.025501697	1.3812	0.008429
184	2-Isopropyl-5-methylphenol acetate	6.4085	C09909	neg	429.2265819	0.020749602	1.3737	0.003568
185	2,4-dimethylhexanedioylcarnitine	6.3025	-	neg	338.1590164	0.014897691	1.3673	0.03826
186	Diethyl Phthalate	6.2171	C14175	neg	221.081746	0.02377594	1.3605	0.03861
187	4-(3-Hydroxybutyl)-3,3,5-trimethylcyclohexanone	6.0907	-	neg	257.1760259	0.016123533	1.3466	0.000333
188	Captopril-cysteine disulfide	5.9766	-	neg	357.0548213	0.011315392	1.3368	0.02642
189	1-Ethenylhexyl butanoate	5.9295	-	neg	197.1543213	0.0455757	1.3358	0.03188
190	4-[(2,4-Dihydroxy-3,3-dimethylbutanoyl)amino]butanoic acid	5.9125	-	neg	254.1012363	0.018168129	1.3346	0.04289
191	5'-O-beta-D-Glucosylpyridoxine	5.6425	C03996	neg	352.0994382	0.089676832	1.3321	0.0008837
192	N-lactoyl-Tryptophan	5.2098	-	neg	275.1042094	0.004427243	1.3295	0.0123
193	7-Methylinosine	5.1669	C05276	neg	304.0807127	0.038022335	1.3214	0.001119
194	N-Lactoylphenylalanine	5.1669	-	neg	236.092927	0.012972368	1.3211	0.01537
195	Glucosyl (E)-2,6-Dimethyl-2,5-heptadienoate	5.1009	-	neg	361.1510795	0.048869248	1.3159	0.0004447
196	R-limonene	5.0661	C06099	neg	229.1080777	0.02431478	1.3054	0.02197

197	1-Deoxy-1-morpholino-D-fructose	5.0013	-	neg	270.0963416	0.017518707	1.3019	0.009046
198	Leu-Pro-Ile	4.92	-	neg	378.1775369	0.021233895	1.2963	0.01224
199	3-[[(2S)-2,4-Dihydroxy-3,3-dimethylbutanoyl]amino]propanoic acid	4.899	-	neg	240.0854366	0.015772683	1.295	0.007254
200	1,4-Cyclohexanedicarboxylic Acid	4.5601	-	neg	171.0657655	0.006228273	1.2937	0.02735
201	N-Lactoylvaline	4.1532	-	neg	170.0818496	0.005273142	1.288	0.03855
202	Aciclovir	4.0815	C06810	neg	260.0543759	0.006730919	1.2756	0.004846
203	2-n-Propyl-4-oxopentanoic acid	3.9948	C16655	neg	203.0921982	0.023815333	1.2653	0.028
204	Adipate semialdehyde	3.958	C06102	neg	319.1402125	0.002229479	1.2623	0.01532
205	Sarmentosin	3.8968	C08340	neg	256.0805472	0.026053144	1.2598	0.02831
206	Proline betaine	3.8968	C10172	neg	188.0924458	0.008321636	1.2595	0.01235
207	2-Isopropylmalic Acid	3.6873	C02504	neg	175.0607056	0.007377789	1.2584	0.01918
208	4-Vinylphenol	3.5789	C05627	neg	119.0495767	0.008895011	1.2543	0.0238
209	Tyrosine lactate	3.5585	-	neg	252.0880182	0.003801905	1.2467	0.0366
210	Indoxyl Sulfate	3.4549	-	neg	212.002153	0.001777996	1.2372	0.03835
211	3-Hydroxypropyl methacrylate	3.1527	-	neg	189.0764495	0.002478086	1.2345	0.0146
212	Agar	3.0624	C08815	neg	335.1354118	0.014177953	1.2312	0.03502
213	Dihydrocaffeic acid 3-sulfate	3.0624	-	neg	261.0077712	0.007501578	1.2238	0.02137
214	1-Cyano-2-hydroxy-3-butene	2.9103	-	neg	253.1197308	0.062027105	1.2151	0.006506
215	Gamma-Glutamylfelinylglycine	2.83	-	neg	392.1502916	0.069032722	1.2135	0.01584
216	2-[4-(sulfooxy)phenyl]acetic acid	2.9924	-	neg	230.9969381	0.008204372	1.2083	0.04736

217	N-Acetylmannosamine	2.5852	C00645	neg	202.0717793	0.022547835	1.2036	0.03232
218	Fructosyl-lysine	2.3837	C16488	neg	289.1409705	0.008437041	1.203	0.02024
219	Voglibosa	2.0177	-	neg	248.1139494	0.061399444	1.2017	0.0007733
220	(E)-indol-3-ylacetaldoxime	2.0177	C02937	neg	219.0772935	0.075478716	1.2015	0.03019
221	Trifluoroquinolone	2.0137	-	neg	443.0444174	0.035132401	1.197	0.002567
222	3-Oxoadipic acid	2.0137	C00846	neg	205.0350993	0.026193743	1.1936	0.03994
223	S-Adenosyl-L-homocysteine	1.7122	C00021	neg	383.1151434	0.00479503	1.1923	0.001662
224	D-Xylulose	1.6934	C00310	neg	171.0269265	0.001846634	1.1922	0.03959
225	Citramalic Acid	1.6061	C00815	neg	147.029324	0.002583483	1.1854	0.009116
226	Kyotorphin	1.5249	C02993	neg	374.1210425	0.013355862	1.1846	0.04888
227	5-Hydroxymethyl-2'-deoxyuridine	1.2632	-	neg	279.0601702	0.011185914	1.1838	0.04058
228	Ascorbic acid 2-sulfate	0.9289	-	neg	254.9819197	0.019627225	1.1819	0.03022
229	Alanylleucine	0.9055	-	neg	247.1301128	0.010950663	1.1586	0.0339
230	Leucyl-Alanine	0.6831	-	neg	247.1300779	0.000263732	1.1538	0.01068
231	Stachyose	0.6753	C01613	neg	665.216898	0.008471457	1.1526	0.00688
232	L-Sorbinose	0.6365	C08356	neg	179.0556129	0.015221436	1.1461	0.01054
233	N-(2-hydroxymethyl-3-chloro-4-hydroxyphenyl)anthranilic acid	0.6287	-	neg	314.0192444	0.052814235	1.1413	0.006112
234	Theophylline	0.6287	C07130	neg	215.0326555	0.007564239	1.1392	0.002413
235	1-[Amino-(3,4-dichloroanilino)methylidene]-2-propan-2-ylguanidine	0.6132	-	neg	286.0605295	0.015021123	1.1371	0.004645
236	4'-Hydroxydiclofenac	4.0815	-	neg	291.9922247	0.031955971	1.134	0.0149

237	Glutaminylproline	0.6055	-	neg	288.1204483	0.003244064	1.1319	0.03018
238	D-Galactaric acid	0.6055	C00879	neg	209.0299832	0.009693443	1.1125	0.02157
239	N-carboxymethyllysine	0.5821	-	neg	203.10343	0.024480515	1.108	0.01442
240	Gluconic Acid	0.6287	C00257	neg	195.050621	0.003252328	1.1057	0.02816
241	Glucoheptonic acid	0.6365	-	neg	225.0616489	0.008903303	1.1043	0.01044
242	2-Hydroxydecanedioic acid	5.2812	-	neg	217.1079858	0.017575524	1.1031	0.007488
243	Azelaic Acid	5.836	C08261	neg	187.0971517	0.013366124	1.0954	0.01035
244	2-Hydroxycampholonic acid	5.9821	-	neg	199.0972477	0.010470279	1.0924	0.006565
245	Decanedioic acid	6.043	C08277	neg	201.1129064	0.018251359	1.0827	0.01195
246	1-Carboxycyclohexaneacetic Acid	5.5774	-	neg	185.0814948	0.010783409	1.0759	0.04408
247	Traumatic Acid	6.1536	C16308	neg	227.1288008	0.031208226	1.0644	0.01571
248	Undecanedioic acid	6.1481	-	neg	215.1286583	0.022284672	1.0617	0.004874
249	Polyribosylribitolphosphate	0.621	-	neg	268.9836489	0.016128653	1.0609	0.02569
250	H-Hyp-gly-OH	0.6441	-	neg	233.0778454	0.058156938	1.0545	0.000181
251	(S)-5-Amino-3-oxohexanoate	0.6441	C03656	neg	289.1409022	0.002680854	1.0531	0.02776
252	Asperulosidic acid	0.6597	-	neg	453.1025987	0.053009163	1.0524	0.004413
253	Gamma-Glutamylglutamine	0.6674	C05283	neg	274.1048872	0.011024361	1.0382	0.01694
254	(2S,3S,4S,5R)-3,4,5-Trihydroxy-6-sulfooxyoxane-2-carboxylic acid	0.6907	-	neg	254.9818695	0.003113326	1.0263	0.008548
255	2-Amino-3-oxoadipate	0.8274	C05520	neg	196.0224798	0.010419132	1.0251	0.03777
256	8-Hydroxyguanine	0.9515	C20155	neg	166.0364965	0.006244442	1.0233	0.0369
257	1-deoxy-L-glycero-tetrulose 4-	0.9708	C15556	neg	183.0060183	0.127033524	1.0225	0.04218

	phosphate							
258	3-Hydroxybutanoic Acid	2.0137	C01089	neg	103.0393529	0.030163711	1.0191	0.01359
259	Ascorbic acid	0.9979	C00072	neg	351.0577308	0.027513249	1.0094	0.01646
260	DL-Glycerol 1-phosphate	0.9738	C00093	neg	171.0059484	0.020054806	1.0053	0.02605
261	Dhurrin	0.6907	C05143	neg	356.099343	0.004477511	1.0042	0.003402
262	Glycerol 3-Phosphate	0.6132	C00093	neg	171.005864	0.004395893	1.0038	0.0376

Supplementary Table S6

Table S6. 61 endogenous metabolites.

No.	Metabolite	RT/min	KEGG Compound ID	Mode	M/Z	CAS ID
1	Hydroxyprolyl-Valine	1.4374	-	pos	213.1237882	-
2	LysoPE(22:5(4Z,7Z,10Z,13Z,16Z)/0:0)	6.6297	-	pos	550.2924378	-
3	Lactacystin	2.8293	-	pos	394.1654068	-
4	Campesterol glucoside	2.8843	-	pos	304.200768	-
5	LysoPE(P-18:0/0:0)	6.9792	-	pos	488.3128517	174062-73-8
6	S-Butylcysteine sulfoxide	3.0518	-	pos	176.07449	-
7	Hexenoylcarnitine	4.7603	-	pos	240.1599588	-

8	Myosmine	5.7612	C10160	pos	310.2020963	532-12-7
9	(4Z,7Z,10Z,13Z,16E,18E)-20-Hydroxydocosa-4,7,10,13,16,18-hexaenoylcarnitine	6.2342	-	pos	470.3278823	-
10	PC(22:6(4Z,7Z,10Z,13Z,16Z,19Z)/P-18:0)	6.7489	C00157	pos	818.6102335	-
11	(S)-Laudanosine	6.9238	-	pos	375.2280262	2688-77-9
12	PS(22:6(5Z,8E,10Z,13Z,15E,19Z)-2OH(7S,17S)/22:2(13Z,16Z))	7.5916	-	pos	964.5309814	-
13	LysoPE(18:1(11Z)/0:0)	6.8206	-	pos	462.2968822	-
14	PA(i-24:0/i-13:0)	6.3133	C00416	pos	782.5616347	-
15	Beta-Thujaplicin	4.595	C09904	pos	165.0913345	499-44-5
16	N-butanoyl-lhomoserine lactone	4.1441	-	pos	172.0971545	-
17	Glutamyltryptophan	3.999	-	pos	334.1409367	38101-59-6
18	S-Prenyl-L-cysteine	3.7408	C06751	pos	190.0903097	5287-46-7
19	Gamma-Glu-leu	3.4766	-	pos	261.1451949	2566-39-4

20	P-Coumaraldehyde	2.6012	C05608	pos	190.0866753	2538-87-6;20711-53-9
21	Pyroglutamic Acid	0.8882	C01879	pos	130.0502689	98-79-3
22	Niflumic Acid	0.6971	C13698	pos	327.0323604	4394-00-7
23	Octopine	0.6656	C04137	pos	247.1404892	34522-32-2
24	D-Galactosamine	0.5878	C02262	pos	162.0763931	7535-00-4
25	3-Hydroxy-C10-Homoserine Lactone	6.3053	-	pos	272.1863077	-
26	PC(22:5(4Z,7Z,10Z,13Z,16Z)/18:3(9Z,12Z,15Z))	7.3605	C00157	pos	852.5559926	-
27	Glutamylglutamic acid	0.6892	C01425	pos	277.1037615	3929-61-1
28	Lunatone	1.0079	-	pos	371.1140931	-
29	L-2-Amino-3-oxobutanoic acid	0.6498	C03508	pos	235.0928606	-
30	4-Hydroxy-L-Proline	0.6578	C01157	pos	114.0554998	51-35-4
31	(R)-(+)-2-Pyrrolidone-5-carboxylic acid	1.254	-	pos	130.0502942	-

32	L-Cystine	0.5958	C00491	pos	241.0315596	56-89-3
33	N-Formyl-L-glutamic acid	1.2421	C01045	neg	196.0223605	1681-96-5
34	2-Amino-3-phosphonopropionic acid	1.689	C05672	neg	205.9628806	5652-28-8
35	Isopropylmaleic acid	2.1163	C02631	neg	203.0558173	44976-69-4
36	Galactosylglycerol	2.607	C05401	neg	235.0823588	-
37	1-O-Feruloyl-beta-D-glucose	0.6597	C17759	neg	377.0858643	-
38	2-Hydroxy-3-Methylbutyric Acid	3.2288	-	neg	117.0550488	4026-18-0
39	L-Acetylcarnitine	3.4119	C02571	neg	202.1082843	3040-38-8
40	6-Hydroxyhexanoic acid	4.5601	C06103	neg	131.0707297	1191-25-9
41	Gamma-D-Glutamylglycine	0.6597	-	neg	203.067031	-
42	2-Amino-3-cyclohexylpropanoic acid	5.6425	-	neg	216.1239848	-
43	Arginylglycine	6.1297	-	neg	266.1012657	2418-67-9
44	Leu-Arg-Asn-Arg	6.6303	-	neg	594.2830552	-

45	Cholesterol glutamate	6.6738	-	neg	550.3530215	-
46	PE(22:5/0:0)	6.6303	-	neg	526.2951223	-
47	5'-O-beta-D-Glucosylpyridoxine	5.6425	C03996	neg	352.0994382	-
48	Leu-Pro-Ile	4.92	-	neg	378.1775369	-
49	N-Lactoylvaline	4.1532	-	neg	170.0818496	21753-44-6
50	1-Cyano-2-hydroxy-3-butene	2.9103	-	neg	253.1197308	7451-85-6
51	Gamma-Glutamylfelinylglycine	2.83	-	neg	392.1502916	-
52	N-Acetylmannosamine	2.5852	C00645	neg	202.0717793	7772-94-3
53	Voglibosa	2.0177	-	neg	248.1139494	-
54	5-Hydroxymethyl-2'-deoxyuridine	1.2632	-	neg	279.0601702	-
55	N-(2-hydroxymethyl-3-chloro-4-hydroxyphenyl)anthranilic acid	0.6287	-	neg	314.0192444	-
56	1-[Amino-(3,4-dichloroanilino)methylidene]-2-propan-2-ylguanidine	0.6132	-	neg	286.0605295	-
57	H-Hyp-gly-OH	0.6441	-	neg	233.0778454	-
58	(S)-5-Amino-3-oxohexanoate	0.6441	C03656	neg	289.1409022	19355-

						90-9
59	2-Amino-3-oxoadipate	0.8274	C05520	neg	196.0224798	-
60	8-Hydroxyguanine	0.9515	C20155	neg	166.0364965	5614-64-2
61	1-deoxy-L-glycero-tetrulose 4-phosphate	0.9708	C15556	neg	183.0060183	-
